# Supplementary material for: C7-Substituted Quinolines as Potent Inhibitors of AdeG Efflux Pumps in Acinetobacter baumannii
Source: ACS Infect Dis. 2025 Feb 27;11(3):626–38. doi: 10.1021/acsinfecdis.4c00705 (PMC11915368; doi:10.1021/acsinfecdis.4c00705)
Supplement: Supplementary file 1 — id4c00705_si_001.pdf [file id4c00705_si_001.pdf]

Supporting Information

C7-substituted Quinolines as Potent Inhibitors of AdeG Efflux Pumps in *Acinetobacter baumannii*

Yiling Zhu[1,2], Charlotte K Hind[1], Taha Al-Adhami[2], Matthew E. Wand[1], Melanie Clifford[1], J. Mark Sutton[1,2]*, Khondaker Miraz Rahman[2]*

Addresses:

[1] UK Health Security Agency, Research and Development Institute, National Infection Service, Porton Down, Salisbury, Wiltshire, SP4 0JG, UK.

[2] Institute of Pharmaceutical Science, King's College London, London SE1 9NH, UK

***Corresponding authors:**

JMS: Tel.: +44 (0) 1980 612649; e-mail: mark.sutton@phe.gov.uk

KMR: Tel: +44 (0) 2078 481891; e-mail: k.miraz.rahman@kcl.ac.uk

Supplementary Tables

Table S1. The collection of bacterial strains that were used in this study.

Species	***Strain***	***Genotype/mutation***	***Strain name used in this study***	***Source / Reference***
A. baumannii	NCTC13421	AYE	AYE	NCTC
A. baumannii	AYEΔ*adeB*	Δ*adeB* mutant	AYEΔ*adeB*	Richmond et al 2016 [7]
A. baumannii	AYEΔ*adeRS*	Δ*adeRS* mutant	AYEΔ*adeRS*	Richmond et al 2016 [7]
A. bumannii	Ab5075-UW	Ab-5075 W/T	Ab5075-UW	Gallagher et al 2015 [47]
A. baumannii	Ab-5075 Cam	Ab-5075 chloramphenicol adapted mutant	Ab5075-CHL	This study
A. baumannii	ABUW_1975	*adeB* transposon mutant	Ab5075Δ*adeB*	Gallagher et al 2015; [47]
A. baumannii	ABUW_1973	*adeR* transposon mutant	Ab5075Δ*adeR*	Gallagher et al 2015; [47]
A. baumannii	ABUW_1972	*adeS* transposon mutant	Ab5075Δ*adeS*	Gallagher et al 2015; [47]
A. baumannii	ABUW_1335	*adeG* transposon mutant	Ab5075Δ*adeG*	Gallagher et al 2015; [47]
A. baumannii	ABUW_1338	*adeL* transposon mutant	Ab5075Δ*adeL*	Gallagher et al 2015; [47]
A. baumannii	ABUW_1336	*adeJ* transposon mutant	Ab5075Δ*adeJ*	Gallagher et al 2015; [47]
A. baumannii	ABUW_1731	*adeN* transposon mutant	Ab5075Δ*adeN*	Gallagher et al 2015; [47]

Table S2. The MICs of different antibiotics on AYE, Ab5075 and their mutants show effects of AdeABC and its regulator AdeRS on efficacy of gentamicin and ciprofloxacin.

**Antibiotic**	**MIC(μg/mL) for strain**											
	**AYE**	**AYE Δ*adeB***	**AYE Δ*adeRS***	**Ab5075-UW**	**Ab5075 Δ*adeB***	**Ab5075 Δ*adeR***	**Ab5075 Δ*adeS***	**Ab5075 Δ*adeG***	**Ab5075 Δ*adeL***	**Ab5075 Δ*adeJ***	**Ab5075 Δ*adeN***	**Ab5075-CHL-adapted**
**GENT**	≥1024	32-64	32-128	≥1024	32-64	32-64	128-256	≥1024	≥1024	≥1024	>1024	512
**RIF**	32	16	16-32	1-4	2-16	2	1	2	1	0.5-2	2	N/A
**CLR**	32-64	8-32	32	16-64	8-32	8-64	16	16	32	16-32	16	
**CHL**	256-512	128-256	256-512	64-128	64	128	128	128	128	128	128-256	512
**IME**	1-2	1	1	16-32	16	16	16	32	16	16	32	N/A
**MER**	1-2	1-2	1	32	32	32	32	64	32	32	32	
**CEFO**	>1024	1024	>1024	>1024	1024	>1024	>1024	>1024	>1024	>1024	>1024	
**CIP**	128	32	32	64	16	32	32	64	64	32	128	

GENT = gentamicin; RIF=rifampicin; CLR=clarithromycin; CHL= chloramphenicol; IME= imipenem; MER= Meropenem; CEFO= cefotaxime; CIP= ciprofloxacin. Numbers shown in red have a reduction in MIC of at least 4-fold for the mutants compared to the corresponding wildtype strain. N/A not tested.

Table S3 The expression levels of efflux pump and their regulator genes in Ab5075 chloramphenicol adapted strains

	adeB	adeR	adeS	adeG	adeL	adeJ	adeN
Ab5075-CHL-1	0.44	1.03	1.01	619.88	4.20	1.16	1.42
Ab5074-CHL-2	0.27	1.01	1.07	595.62	3.95	1.11	1.51

Numbers in red colour suggest the fold change is statistically significant (P value ≤ 0.05).

Table S4. The ∆G and ChemScore for compounds 1 and 3 in the best poses against AdeB and AdeG

	AdeB		AdeG	
**Compounds**	**Chem Score**	**ΔG kcal/mol**	**Chem Score**	**ΔG kcal/mol**
**1**	29.28	-32.95	28.76	-37.42
**3**	29.45	-30.73	30.05	-30.34

Table S5. The MICs and test concentration selected for all synthesised EPIs on AYE and Ab5075-UW based on less than 10% reduction in growth compared to control.

Compound Name	***Conc. tested (µg/mL)***	***MIC***	***Compound Name***	***Conc. tested (µg/mL)***	***MIC***
**1**	25	100	**7**	25	>200
**2**	25	>200	**8**	50	>200
**3**	50	>200	**9**	50	>200
**4**	25	200	**10**	25	200
**5**	12.5 (25 for Ab5075-UW)	100	**11**	50	>200
**6**	100	>200	**12**	100	>200
**1.1**	100	>200	**3.1**	100	>200
**1.2**	100	>200	**3.2**	6.25	12.5
**1.3**	25	200	**3.3**	25	50
**1.4**	25	50	**3.4**	25	50
**1.5**	25	50	**3.5**	25	25
**1.6**	12.5	50	**3.6**	12.5	25
**1.7**	100	200	**3.7**	100	200
**1.8**	25	50	**3.8**	25	50
**1.9**	100	>200	**3.9**	100	>200
**1.10**	50	200	**3.10**	50	>200
**1.11**	50	100	**3.11**	50	100
**1.12**	50	100	**3.12**	50	100
**PAβN**	25	25	**CCCP**	10	10

Table S6. The MICs of chloramphenicol but not gentamicin is reduced in the presence of compound **1** and **3** treatment in a strain overexpressing AdeFGH.

**Antibiotic**	**EPI**	**AYE**	**Ab5075-UW**	**Ab5075-CHL-adapted**	**Ab5075Δ*adeG***	**Ab5075Δ*adeL***
				MICs(μg/mL) for strains		
**GENT**	No EPI	>1024	≥1024	512	512	512
	**1**	≥1024	512			
	**3**	≥1024	512		N/A	
	**4**	≥1024	512			
	**PAβN**	≥1024	512	512	512	512
	**CCCP**	1024	512	512	512	512
**CHL**	No EPI	256-512	128	512	128	128
	**1**	256	64-128	128	128	128
	**3**	128	64-128	64	128	128
	**4**	256	64-128	256	128	128
	**PAβN**	128	64-128	128-256	64	64
	CCCP	256	128	256	128	128

All EPIs compounds were tested at conc. of 25μg/mL The number in red colour show a reduction in MIC of at least 4-fold, compared to the untreated control, due to the addition of EPI. . *N/A not tested.

Table S7. The MICs of rifampicin, clarithromycin and colistin for strain AYE in the presence of PAβN and CCCP and compared to three selected EPI compounds **1**,**3** and **4**.

Antibiotics	***EPIs***	***MIC (μg/mL) for AYE***
**RIF**	No EPI	32
	PAβN	0.125-0.25*
	CCCP	4
	**1**	4-8
	**3**	16
	**4**	8
**CLR**	No EPI	32-64
	PAβN	0.5*
	CCCP	16
	**1**	32-64
	**3**	16
	**4**	16
**CST**	No EPI	0.5
	PAβN	0.5
	CCCP	≤0.125*
	**1**	≤0.125*
	**3**	≤0.125*
	**4**	≤0.125*

The numbers in red colour show the MIC of reduced for at least 4-fold due to the addition of EPI.

Table S8. The primers and prime efficiency for each gene tested in RT-PCR

**Gene name**	**S primer**	**AS Primer**
adeB	GGATTATGGCGACTGAAGGA	AATACTGCCGCCAATACCAG
adeR	TGCACTAGAGCGAACCGTAG	CTATATCCCACGCCACGCAC
adeS	GAATTCACTCCGCCGAAATGT	AACTCATGTGCGATAGCTGC
adeG	AATCGCGACCGCTTGTTTATT	ACCAGATGGCGCGGTTAC
adeL	GGACAGCCCGTATTTTAGCG	CGTCCAATCGATACAGGCACA
adeJ	CATCGGCTGAAACAGTTGAA	GCCTGACCATTACCAGCACT
adeN	ATGCTGTCTCTCTTGACGACAT	ATCGCAGATTGCAGTAAATAAGCC

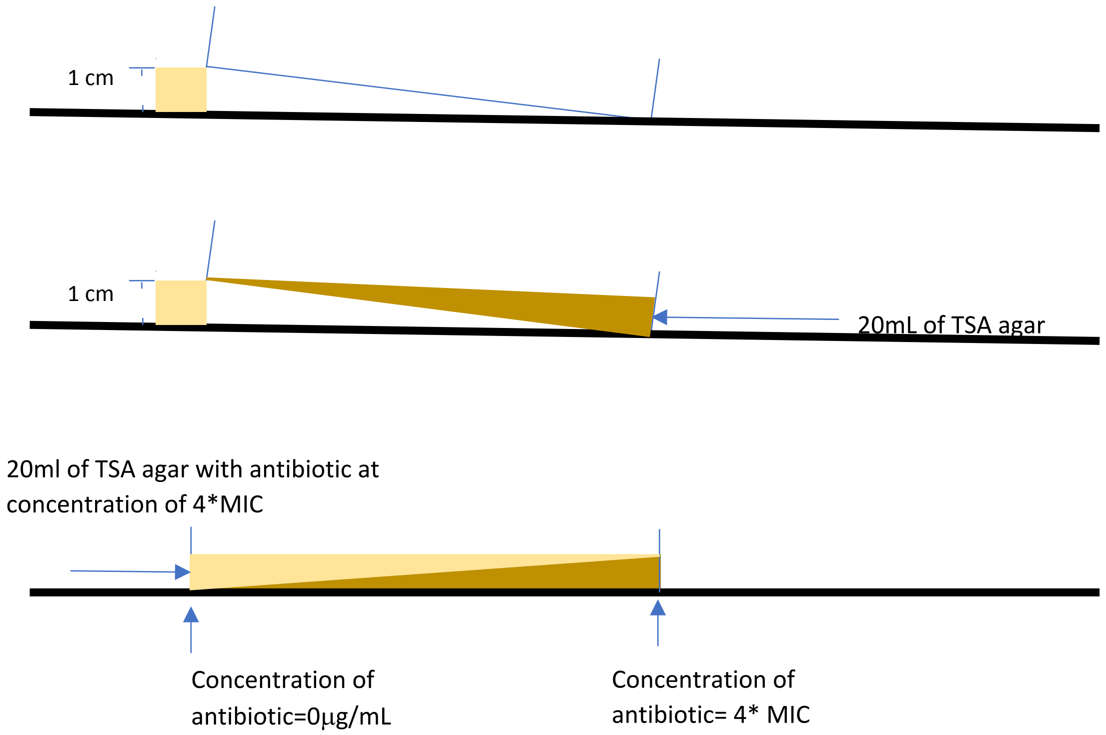

Figure S1. The method of making antibiotic gradient plate.

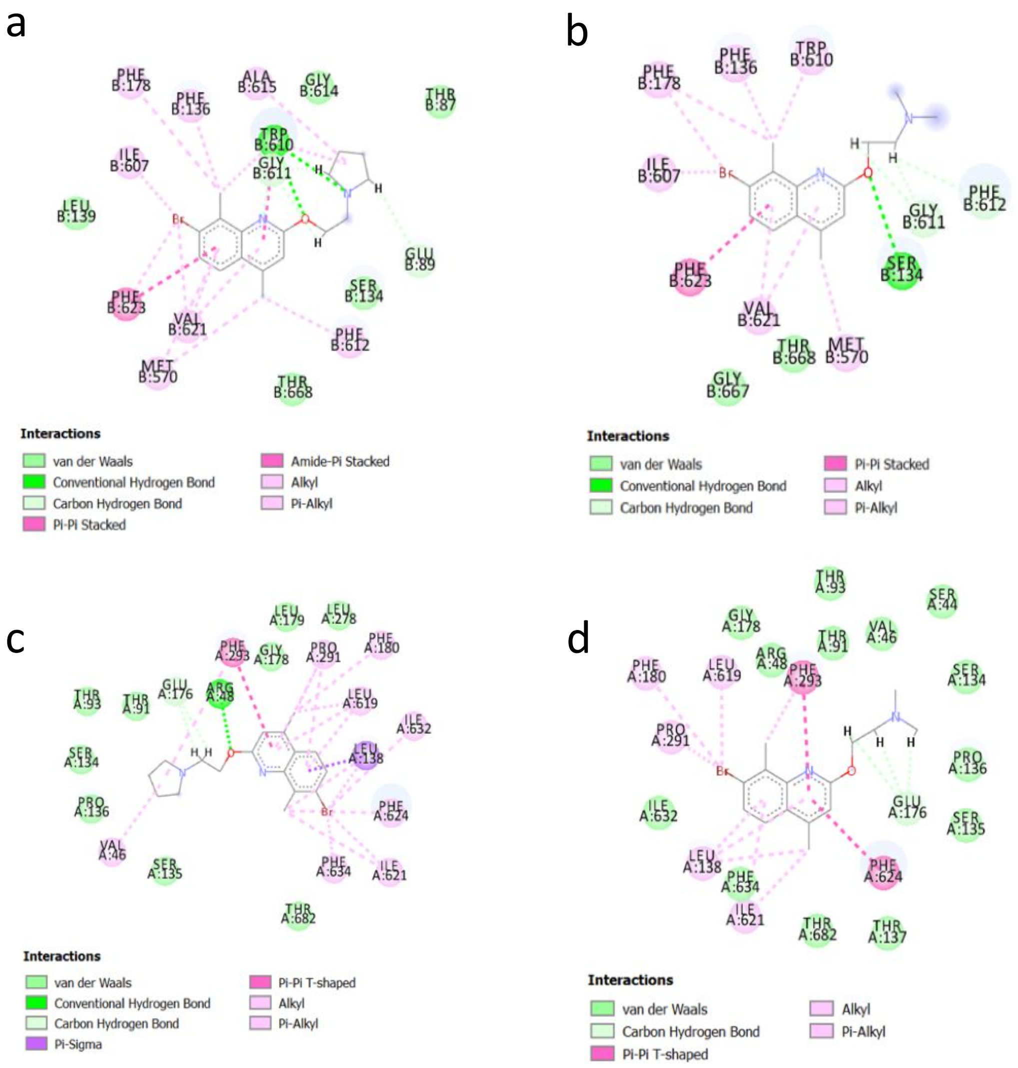

Figure S2. The interaction between compound **1** or **3** and key residues in the efflux complexes. (a)**1** and AdeB complexes; (b) **3** and AdeB complexes; (c) **1** and AdeG complexes; (d) **3** and AdeG complexes.

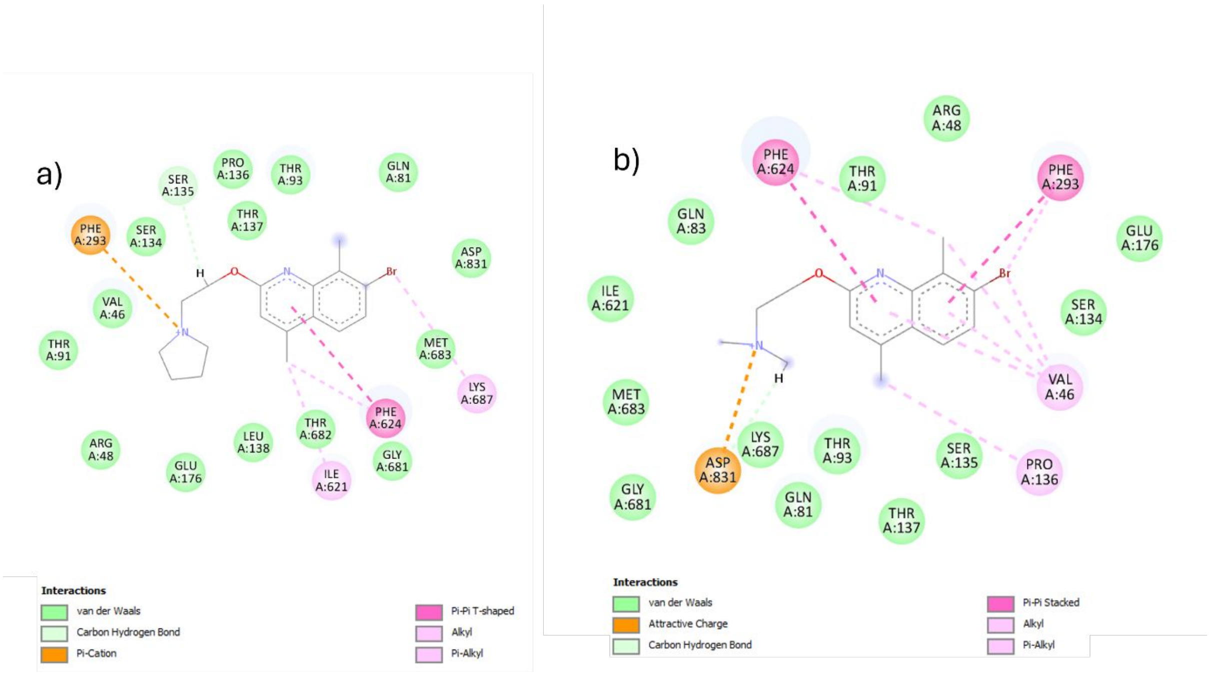

Figure S3: 2D interaction map of structurally optimised docked **1**:AdeG and **3**:AdeG complexes showed that the ligands remained within the hydrophobic region of the distal binding pocket, particularly interacting with the Phe loop of the AdeG efflux pump.

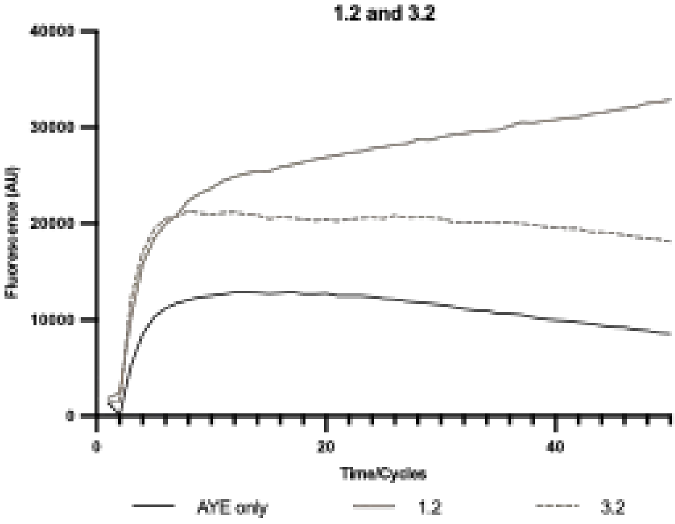

1.2 and 3.2
40000
30000
20000
10000
0
Fluorescence (AU)
0
20
40
Time/Cycles
AYE only
1.2
3.2

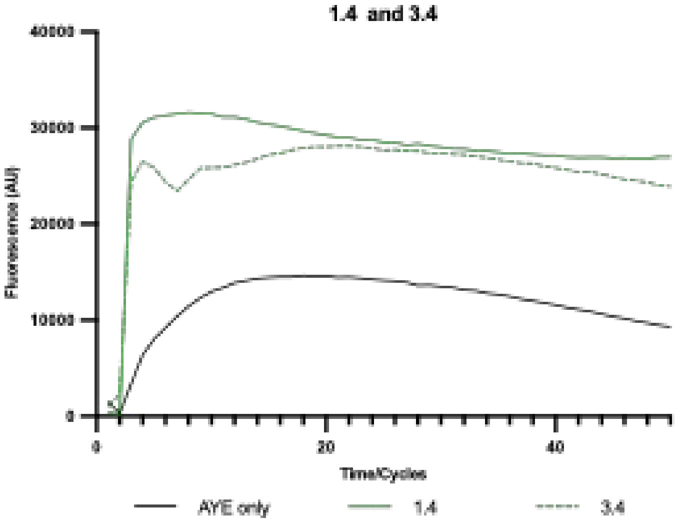

1.4 and 3.4
40000
30000
20000
10000
0
Fluorescence (AU)
0
20
40
Time/Cycles
AYE only
1.4
3.4

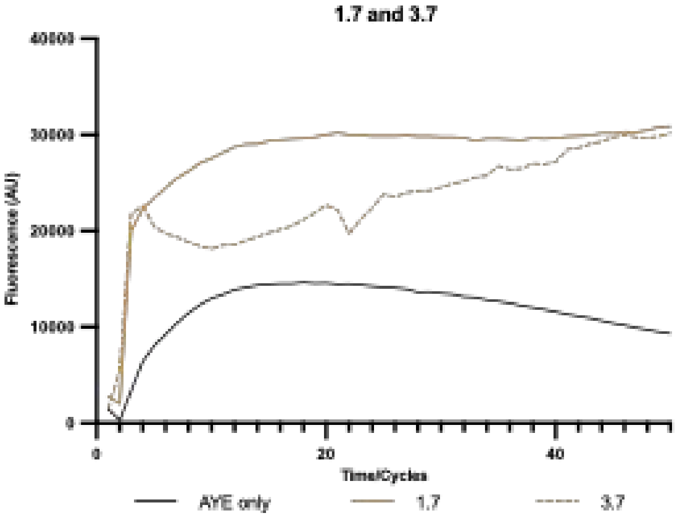

1.7 and 3.7
40000
30000
20000
10000
0
Fluorescence (AU)
0
20
40
Time/Cycles
AYE only
1.7
3.7

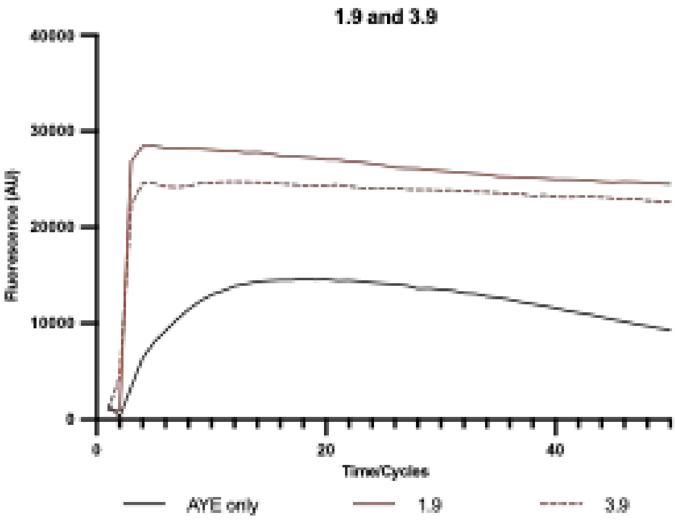
1.9 and 3.9
Fluorescence (AU)
40000
30000
20000
10000
0
0
20
40
Time/Cycles
AYE only
1.9
3.9

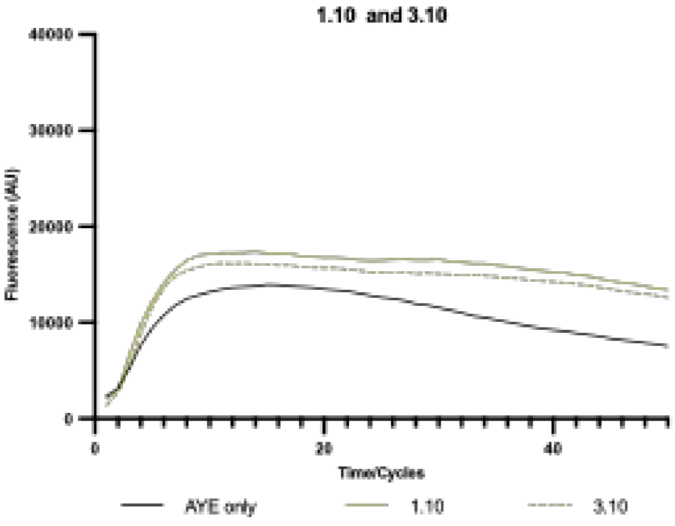
1.10 and 3.10
Fluorescence (AU)
40000
30000
20000
10000
0
0
20
40
Time/Cycles
AYE only
1.10
3.10

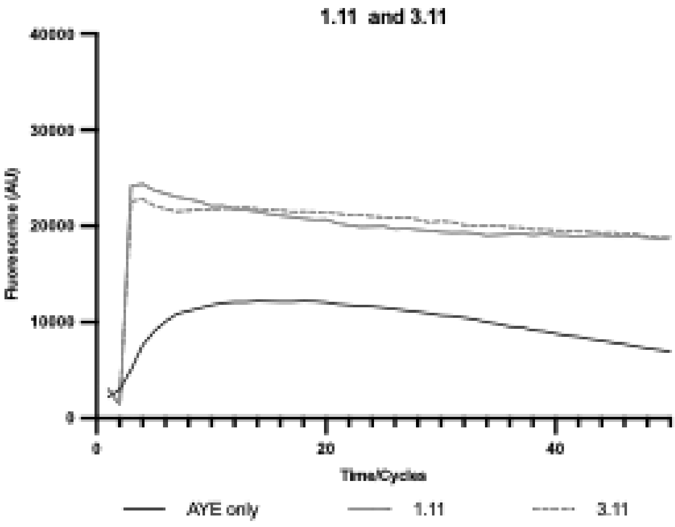
1.11 and 3.11
Fluorescence (AU)
40000
30000
20000
10000
0
0
20
40
Time/Cycles
AYE only
1.11
3.11

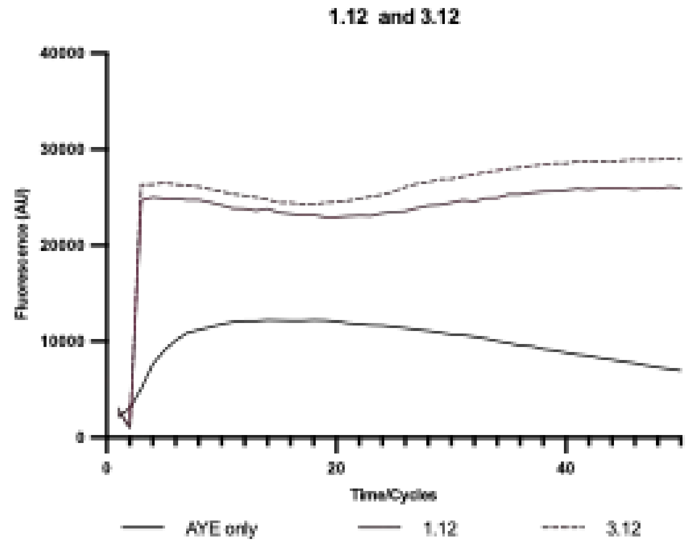

Figure S4. Hoechst accumulation in AYE cells with the addition of 14 synthesised second-generation EPI compounds. The EPI compounds were added to the cell suspension at the maximum concentration which did not adversely affect growth and incubated for 15 min before the addition of HOECHST dyes. Fluorescence levels were then recorded every 2 min 33 sec for 50 cycles. Cells with compounds with the same R_3 group but different R_2 are shown in the same colour, with Br substitutions shown as solid lines (R_2=quinoline), compared to dotted lines for EPIs with no substitution (R_2=amine). All results were performed in triplicates and the curve presented is the fluorescence value of three biological repeats after being blanked against cell free PBSM+G with HOECHST DYE; for clarity error bars are not shown on the graph, but the SD is included in the end-point measurements shown in table 2.

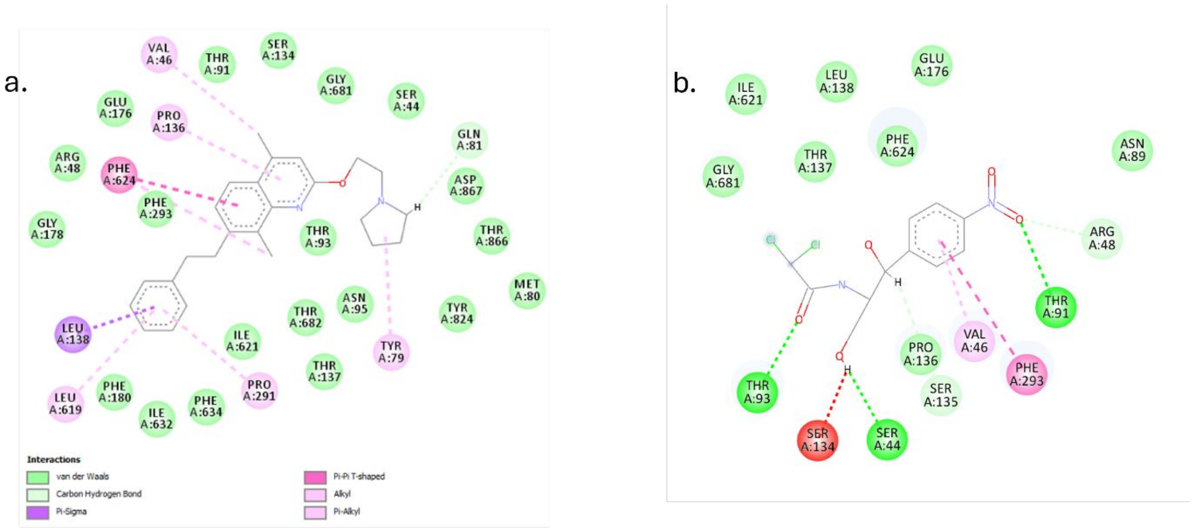

Figure S5. 2D interaction map of docked complexes of **1.3** and chloramphenicol to the AdeG efflux pump, showing that they occupy adjacent binding sites with some interacting residues common between chloramphenicol and **1.3**.

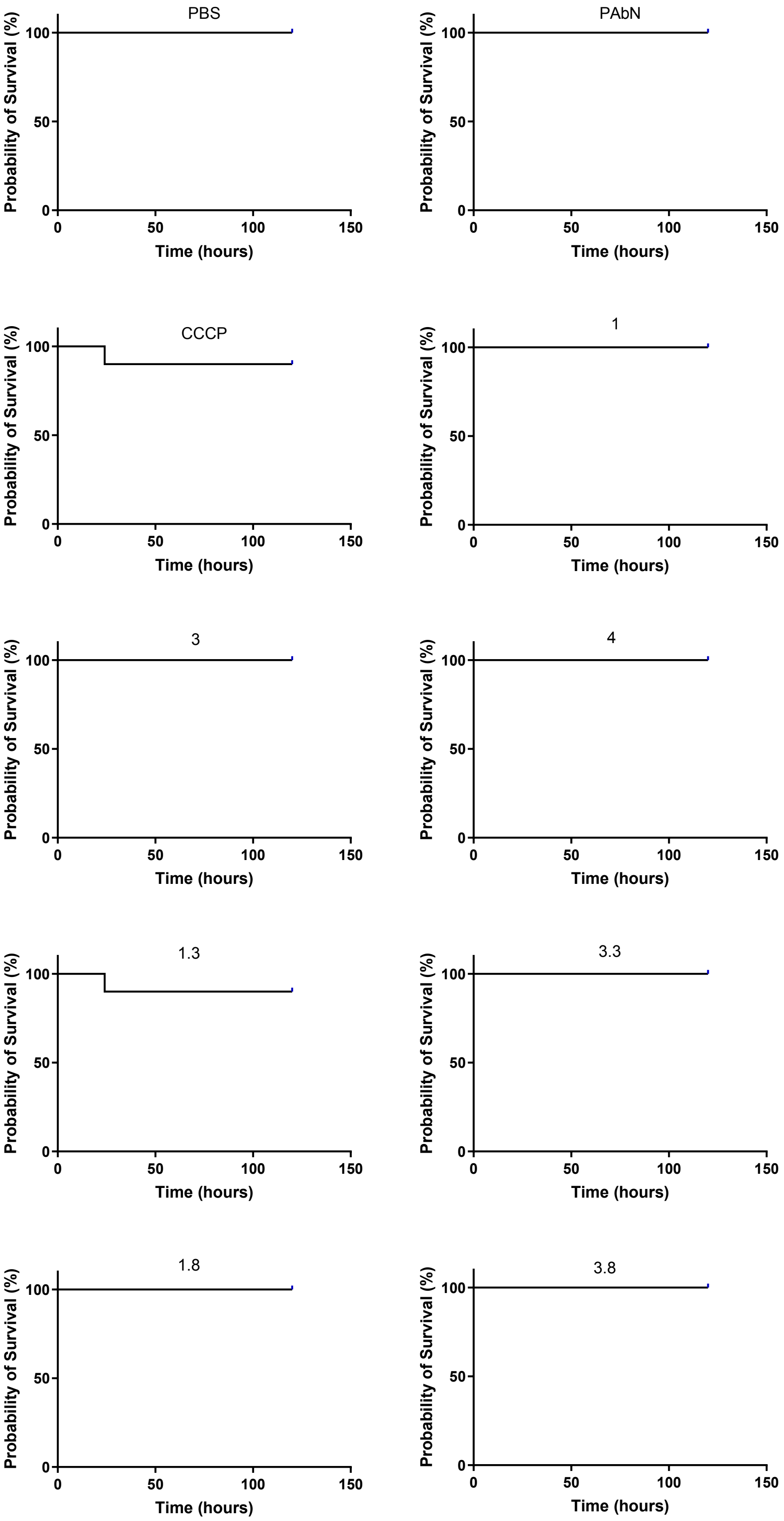

Figure S6: Survival curve for the tested compounds in *Galleria melonella* toxicity model.

Chemistry experimental section

Purity determination of synthesized final compounds

The level of purity of the compounds for biological testing has been evaluated through LC-MS analysis, using two different gradient methods, reported hereafter. LC-MS analyses were performed on a Waters Alliance 2695 system (from Waters), with elution in gradient. HPLC grade solvents were used as mobile phase while a Monolithic C18 50 X 4.60 mm column (from Phenomenex) was used as stationary phase. UV detection was performed using a Waters 2996 photo array detector (from Waters). Injection volume has been set to 10 µL. The compounds have been dissolved in a mixture of H2O/ACN (50/50, v/v) or DMSO/ACN (50/50, v/v) accordingly to the solubility. The area of the peak corresponding to the compound has been automatically determined by the software included in the LC-MS system. The eventual presence of solvent UV trace has been subtracted to the total in order to determine the percentage of purity. All compounds showed at least 95% purity in both methods.

LC-MS methods:

Method A: flow 0.5 mL/min

A) water + 0.1 % formic acid

B) acetonitrile + 0.1% formic acid

Time (min)	0	3	3.5	4.5	5
A (%)	95	10	5	5	95
B (%)	5	90	95	95	5

Method B: flow 1 mL/min

A) water + 0.1 % formic acid

B) acetonitrile + 0.1% formic acid

Time (min)	0	2	5	6	7.5	9	10
A (%)	95	95	50	50	5	95	95
B (%)	5	5	50	50	95	5	5

7-bromo-4,8-dimethyl-2-(2-(pyrrolidin-1-yl)ethoxy)quinoline (1)

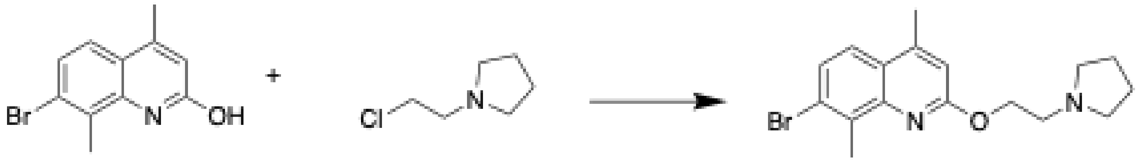

1

7-bromo-4,8-dimethylquinolin-2-ol (100 mg, 39.7 mmol) was mixed with 1-(2-chloroethyl)pyrrolidine (63.6 mg, 47.6 mmol) and K_2CO_3 (164.6 mg, 119.1 mmol), followed by addition of 20 ml acetone. The mixture was heated to reflux overnight or until TLC (DCM/ MeOH 9:1) showed complete consumption of the starting material. Upon cooling, solvent was evaporated. The product was extracted with EtOAc, dried over $MgSO_4$ and concentrated by rotary evaporation. Purification by flash column chromatograph was used to afford the title compound (108 mg, 30.9 mmol, yield=78^{1}H-NMR (400MHZ, CD_3OD, ppm) δ: 1.9-2.1 (m,4H), 2.56 (s,3H), 2.71 (s,3H), , 3,62 (t, *J*=6.0Hz, 6.0Hz, 2H), 4.72(t, *J*=6Hz,6Hz, 2H), 6.85 (s, 1H), 7.42 (d, *J*=8.8Hz, 1H), 7.65 (d, *J*=9.2Hz,1H). ^{13}C-NMR (101MHz, Chloroform-*d,* ppm) δ:161.2, 147.0, 146.0, 135.4, 127.4, 125.7, 124.1, 122.1, 112.9, 64.4, 57.9, 54.9, 54.7, 54.2, 23.5, 22.7, 18.9, 17.8.HR-MS, m/z calc. for $C_{17}H_{21}BrN_2O$ $(M)^+$ 349.1722 found 349.3711 $([M]+H)^+$.

7-bromo-4,8-dimethyl-2-(naphthalen-1-ylmethoxy)quinoline (2).

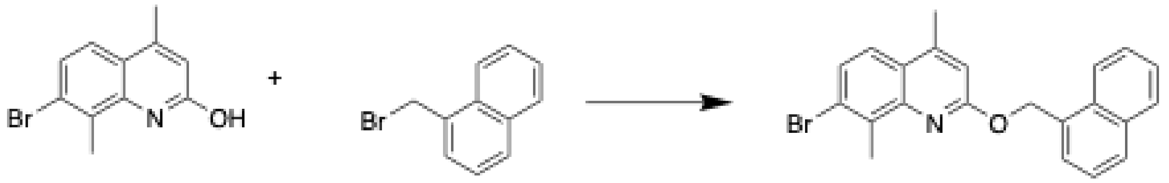

2

Compound **2** was obtained with 7-bromo-4,8-dimethylquinolin-2-ol (100mg, 39.7mmol) and 1-(bromomethyl)naphthalene (105.2 mg, 47.6 mmol) under the same condition of compound **1**. (56 mg, 14.3 mmol, yield=36%). ^{1}H-NMR (400MHZ, DMSO-*d₆,* ppm) δ: 2.59 (s, 3H), 2.80 (s, 3H), 5.99 (s, 2H), 6.98 (s, 1H), 7.52 (t, *J*=7.6Hz, 8Hz, 1H), 7.58 (t, *J*=7.2Hz, 6Hz, 2H), 7.63 (d, *J*=9.2Hz, 1H), 7.75 (d, *J*=8.4Hz, 2H), 7.77 (s, 1H), 7.97 (d *J*=9.6Hz, 1H), 8.13 (d, *J*=2.4Hz, 1H). ^{13}C-NMR (101MHz, DMSO-*d₆,* ppm) δ: 160.8, 148.3, 145.2, 134.1, 133.3, 132.6, 131.3, 128.7, 128.5, 127.5, 127.4, 126.5, 125.9, 125.34, 125.29, 124.0, 123.8, 123.2, 112.7, 65.2, 18.3, 17.6. HR-MS, m/z calc. for $C_{22}H_{18}BrNO$ $(M)^+$ 392.0876 found 392.9897 $([M]+H)^+$.

2-((7-bromo-4,8-dimethylquinolin-2-yl)oxy)-*N*,*N*-dimethylethan-1-amine (3).

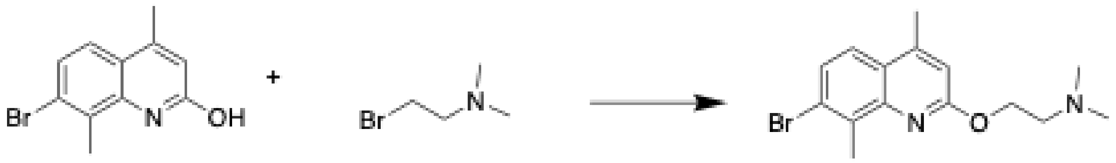

3

7-bromo-4,8-dimethylquinolin-2-ol (100 mg, 39.7 mmol) and K_2CO_3 (164.6 mg, 119.1 mmol) was mixed in 10 mL DMF, and the solution was heated up at 100°C for 2 hrs. 2-bromo-*N*,*N*-dimethylethan-1-amine (72.4 mg, 47.6 mmol) was then added into the solution. The mixture was heated to reflux overnight. The reaction was cooled to room temperature and extracted with EtOAc and water. The organic layer was separated, dried over $MgSO_4$ and evaporated. The residue was purified by flash column chromatography to obtain the product as white solid (62 mg, 19.2 mmol, yield=48%). ^{1}H-NMR (400MHZ, CD_3OD*,* ppm) δ:2.25 (s, 6H), 2.43 (s, 3H), 2.60 (s, 3H), 2.71 (t, *J*= 5.6Hz, 5.6Hz, 2H), 4.45 (t, *J*=6Hz, 5,6Hz, 2H), 6.65 (s, 1H), 7.34 (d, *J=8*.8Hz, 1H), 7.44 (d, *J*=8.8Hz, 1H). ^{13}C-NMR (101MHz, CD_3OD*,* ppm) δ:162.4, 149.0, 147.0, 136.0, 128.57, 128.60, 125.3, 123.6, 113.7, 63.9, 58.9, 45.86, 18.9, 18.0. HR-MS, m/z calc. for $C_{15}H_{19}BrN_2O$ $(M)^+$ 322.9765 found 323.7734 $([M]+H)^+$

7-bromo-4,8-dimethyl-2-(2-(piperidin-1-yl)ethoxy)quinoline (4).

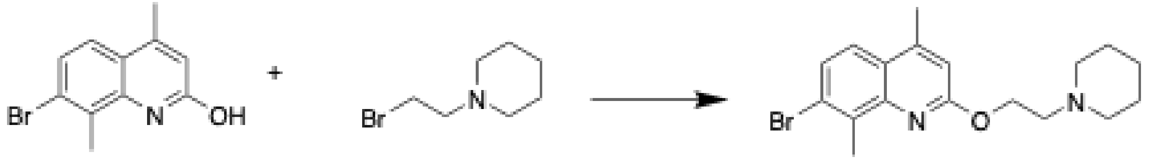

4

7-bromo-4,8-dimethylquinolin-2-ol (100 mg, 39.7 mmol), 1-(2-bromoethyl)piperidine (91.4 mg, 47.6 mmol) and K_2CO_3 (164.6 mg, 119.1 mmol) were mixed in 5 ml DMF in a microwave-proof glass tube. The mixture was heated for 30 mins at 170°C in a microwave synthesis. Upon cooling, the reaction was extracted with EtOAc and water. $MgSO_4$ was used to dry the separated organic layer. The compound was obtained by flash column chromatography (64 mg, 17.6 mmol, yield=44.3%). ^{1}H-NMR (400MHZ, CD_3OD, ppm) δ:1.49-1.50 (m, 2H), 1.62-1.68 (m, 4H), 2.51 (s, 3H), 2.57 (t, *J*=3.2Hz, 5.2Hz, 4H), 2.68 (s, 3H), 2.86 (t, *J*=5.6Hz, 5.6Hz, 3H), 4.57 (t, *J*=5.6Hz, 5.6Hz, 3H), 6.72 (s, 1H), 7.42 (d, *J*=8.4Hz, 1H), 7.52 (d, *J*=8.4Hz, 1H). ^{13}C-NMR (101MHz, DMSO-d_6, ppm) δ: 206.5, 160.87, 148.0, 145.2, 134.0, 127.2, 125.2, 123.8, 123.1, 112.7, 54.2, 40.1, 30.7, 25.3, 18.3, 17.4. HR-MS, m/z calc. for $C_{18}H_{23}BrN_2O$ $(M)^+$ 363.1275 found 364.0189 $([M]+H)^+$

7-bromo-4,8-dimethyl-2-(2-(4-methylpiperazin-1-yl)ethoxy)quinoline (5).

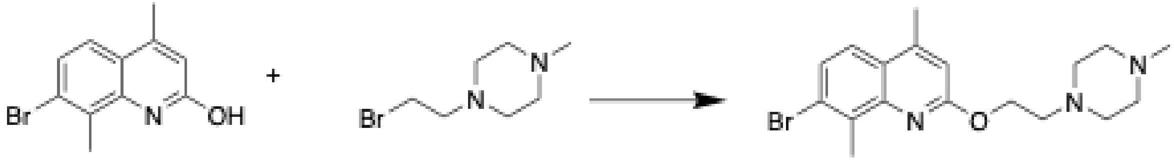

5

7-bromo-4,8-dimethylquinolin-2-ol (100 mg, 39.7 mmol), 1-(2-bromoethyl)-4-methylpiperazine (98.6 mg, 47.6 mmol) and K_2CO_3 (164.6 mg, 119.1 mmol) were mixed in 5 ml DMF in a microwave-proof glass tube. The mixture was heated for 1h at 170°C in a microwave synthesis. Upon cooling, the reaction was extracted with EtOAc and water. The organic layer was collected, dried by $MgSO_4$, and concentrated. The product was obtained by flash column chromatography (80 mg, 21.1 mmol, yield=53.1%). ^{1}H-NMR (400MHZ, Chloroform-*d*, ppm) δ:2.25 (s, 3H), 2.48 (t, *J*=9.2Hz, 5.6Hz, 4H), 2.51 (s, 3H), 2.60 (t, *J*=2.4Hz, 2.1Hz), 2.72 (s, 3H), 2.81 (t, *J*=6Hz, 5.6Hz), 4.55 (t, *J*=6Hz, 6Hz), 6.69 (s, 1H), 7.44 (d, *J*=8.8Hz, 1H), 7.48(d, *J*=9.2Hz, 1H). ^{13}C-NMR (101MHZ, Chloroform-*d*, ppm) δ: 161.1, 147.1, 145.9, 135.4, 127.5, 125.8, 124.1, 122.1, 112.9, 63.1, 57.0, 55.0, 53.4, 50.8, 46.0, 18.9, 17.8. HR-MS, m/z calc. for $C_{18}H_{24}BrN_3O$ $(M)^+$ 378.1437 found 378.1711 $([M]+H)^+$

7-bromo-4,8-dimethyl-2-(quinolin-8-ylmethoxy)quinoline (6).

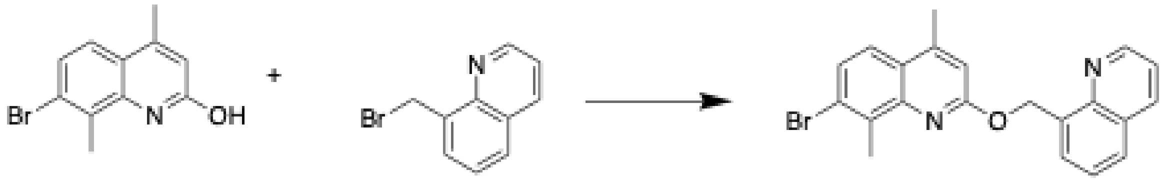

6

The compound was obtained with 7-bromo-4,8-dimethylquinolin-2-ol (100 mg, 39.7 mmol) and 8-(bromomethyl)quinoline (105.7 mg, 47.6 mmol) under the same condition of compound **1** (62 mg, 15.9 mmol, yield=40%). ^{1}H-NMR (400MHZ, Chloroform-*d,* ppm) δ:2.6 (s, 3H), 2.8 (s, 3H), 6.3 (s, 2H), 6.89 (s, 1H), 7.45 (d, *J*=4Hz, 1H), 7.47 (d, *J*=4Hz, 1H), 7.53 (t, *J*=4Hz, 8Hz, 1H), 7.54 (d, *J*=8Hz, 1H), 7.78 (d, *J*=8Hz, 1H), 7.95 (d, *J*=8Hz, 1H), 8.18 (d, *J*=8Hz, 1H), 9.12 (d, *J*=8Hz, 1H). ^{13}C-NMR (101MHZ, Chloroform-*d,* ppm) δ:161.2, 149.8, 147.1, 136.3, 135.8, 135.6, 128.6, 128.2, 127.6, 127.5, 126.3, 125.7, 124.2, 122.1, 121.2, 113.0, 63.7, 18.9, 17.9. HR-MS, m/z calc. for $C_{21}H_{17}BrN_2O$ $(M)^+$ 393.1251 found 394.1243 $([M]+H)^+$

4,8-dimethyl-2-(2-(pyrrolidin-1-yl)ethoxy)quinoline (7).

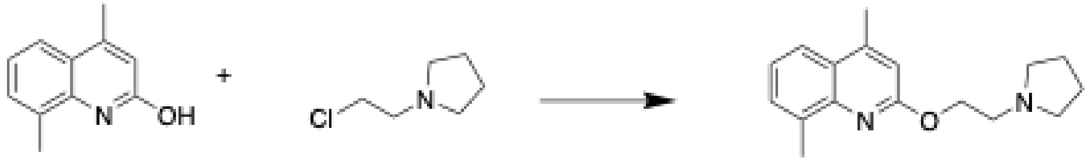

7

4,8-dimethylquinolin-2-ol (100 mg, 57.7 mmol) and K_2CO_3 (239.2 mg, 173,1 mmol) was dissolved in 20 mL DMF, and the solution was heated up at 100°C for 2h. 1-(2-chloroethyl)pyrrolidine (92.5 mg, 69.24 mmol) was then added into the solution. The mixture was heated to reflux overnight. Upon finishing, the reaction was cooled to room temperature, and extracted with EtOAc and water. The organic layer was separated, dried over $MgSO_4$ and evaporated. The residue was purified by flash column chromatography to obtain the product (30 mg, 11.1 mmol, yield=19.2%). ^{1}H-NMR (400MHZ, Chloroform-*d,* ppm) δ: 1.82-1.84 (m, 4H), 2.60 (s, 3H), 2.70 (s, 3H), 2.76 (t, *J*=3.2Hz, 5.2Hz, 4H), 3.25 (t, *J*=6Hz, 6Hz, 2H), 4.70 (t, *J*=6Hz, 6Hz, 2H), 6.81 (s, 1H), 7.29 (t, *J*=5.6Hz, 2Hz, 1H), 7.49 (d, *J*=6.8Hz, 1H), 7.73 (s, *J*=8Hz, 1H). ^{13}C-NMR (101MHz, DMSO-*d$_6$,* ppm) δ: 162.3, 155.6, 146.0, 136.2, 129.7, 123.6, 122.8, 122.1, 113.5, 66.8, 56.9, 56.52, 56.46, 23.56, 23.62, 19.9, 16.9.HR-MS, m/z calc. for $C_{17}H_{22}N_2O$ $(M)^+$ 271.2135 found 272.1733 $([M]+H)^+$

4,8-dimethyl-2-(naphthalen-1-ylmethoxy)quinoline (8).

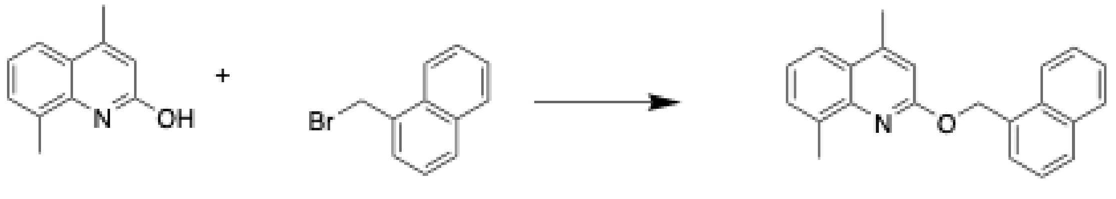

8

4,8-dimethylquinolin-2-ol (100 mg, 57.7 mmol), 1-(bromomethyl)naphthalene (191.4 mg, 86.6 mmol) and K_2CO_3 (160 mg, 115.4 mmol) were mixed in 5 μL DMF in a microwave-proof glass tube. The mixture was heated for 1h at 170°C in a microwave ml synthesis. Upon cooling, the reaction was extracted with EtOAc and water. The organic layer was collected, dried by $MgSO_{4,}$ and concentrated. The product was obtained by flash column chromatography (38 mg, 12.1 mmol, yield=21%). ^{1}H-NMR (400MHZ, Chloroform-*d,* ppm) δ: 2.48 (s, 3H), 2.69 (s, 3H), 5.90 (s, 2H), 6.69 (s,1H), 7.20 (t, *J*=8Hz, 7.6Hz, 1H), 7.36(t, *J*=8Hz, 7.2Hz, 1H), 7.38 (d, *J*=6.8Hz, 1H), 7.43 (t, *J*=8Hz, 8Hz, 2H), 7.62 (d, *J*=7.2Hz, 2H), 7.74 (d, *J*=8.4Hz, 1H), 7.78 (d, *J*=7.6Hz, 1H), 8.11 (d, *J*=8.4Hz, 1H). ^{13}C-NMR (101MHz, Chloroform-*d,* ppm) δ: 160.6, 147.2, 145.3, 135.8, 133.8, 133.3, 132.1, 129.6, 128.9, 128.7, 127.6, 126.4, 125.8, 125.4, 125.3, 124.2, 121.5, 112.8, 65.7, 19.1, 18.4. HR-MS, m/z calc. for $C_{22}H_{19}NO$ $(M)^+$ 314.1333 found 313.1121 $([M]+H)^+$

2-((4,8-dimethylquinolin-2-yl)oxy)-*N*,*N*-dimethylethan-1-amine (9).

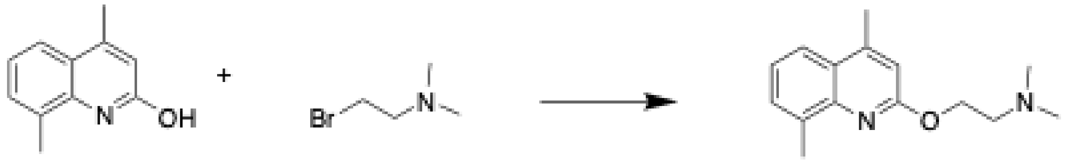

9

4,8-dimethylquinolin-2-ol (100 mg, 57.7 mmol) and 2-bromo-*N*, *N*-dimethylethan-1-amine (131.7 mg, 86.6 mmol) was used to afford the product under the same condition of.**8**. (20 mg, 8.3 mmol, yield=14.3%). ^{1}H-NMR (400MHZ, Chloroform-*d,* ppm) δ: 2.32 (s, 6H), 2.52 (s, 3H), 2.61 (s, 3H), 2.76 (t, *J*=6Hz, 5.6Hz, 2H), 4.55 (t, *J*=5.6Hz, 6Hz, 2H), 6.72 (s, 1H), 7.21 (d, *J*=8Hz, 1H), 7.40 (d, *J*=7.2Hz, 1H), 7.66 (d, *J*=8.4H, 1H). ^{13}C-NMR (101MHz, Chloroform-*d,* ppm) δ: 160.6, 147.0, 145.2, 135.7, 129.5, 125.2, 123.3, 121.4, 112.8, 62.8, 58.1, 45.8, 19.0, 18.2. HR-MS, m/z calc. for $C_{15}H_{20}N_2O$ $(M)^+$ 245.2766 found 246.2153 $([M]+H)^+$

4,8-dimethyl-2-(2-(piperidin-1-yl)ethoxy)quinoline (10).

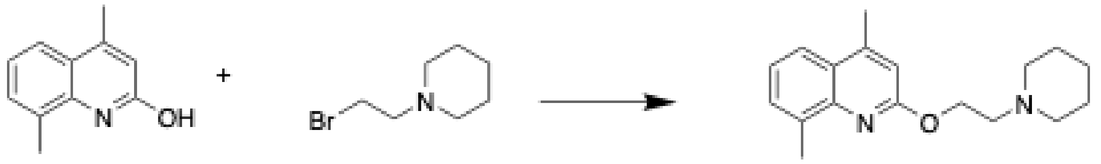

10

4,8-dimethylquinolin-2-ol (100 mg, 57.7 mmol) and 1-(2-bromoethyl)piperidine (166.3 mg, 86.6 mmol) was used to afford the product follow the same condition of **8** (28 mg, 9.8 mmol, yield=17% ^{1}H-NMR (400MHZ, Chloroform-*d,* ppm) δ: 1.33-1.36 (m, 2H), 1.51-1.56 (m, 4H), 2.45 (s, 3H), 2.50 (t, *J*=16Hz, 16Hz, 4H) 2.54 (s, 3H), 2.77 (t, *J*=6Hz, 6Hz, 2H), 2.54 (t, , *J*=6Hz, 6Hz, 2H), 6.61 (s, 1H), 7.12 (t, *J*=5.6Hz, 5.6Hz, 1H), 7.32 (d, , *J*=7.2Hz, 1H), 7.57 (d, , *J*=8Hz, 1H). ^{13}C-NMR (101MHz, Chloroform-*d,* ppm) δ:160.4, 147.0, 145.2, 135.7, 129.5, 125.1, 123.3, 121.4, 112.6, 62.6, 57.6, 54.9, 36.5, 25.6, 23.9, 19.0, 18.2. HR-MS, m/z calc. for $C_{18}H_{24}N_2O$ $(M)^+$ 285.1933 found 2854.9756 $([M]+H)^+$

4,8-dimethyl-2-(2-(4-methylpiperazin-1-yl)ethoxy)quinoline (11).

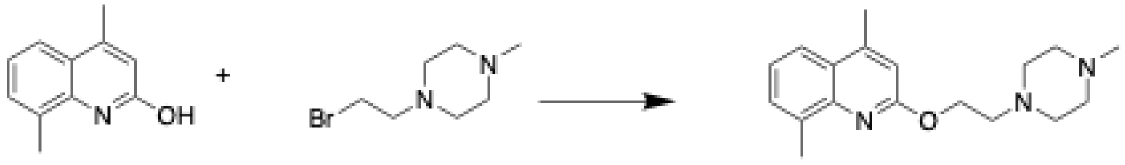

11

4,8-dimethylquinolin-2-ol (100 mg, 57.7 mmol) and 1-(2-bromoethyl)-4-methylpiperazine (179.4 mg, 86.6 mmol) was used to afford the product follow the same condition of **8** (38 mg, 12.7 mmol, yield=22%). ^{1}H-NMR (400MHZ, Chloroform-*d,* ppm) δ: 2.18 (s, 3H), 2.45 (t, *J*=9.2Hz, 6Hz,4H), 2.53 (t, *J*=9.2Hz, 5.6Hz,4H), 2.74 (t, *J*=6Hz, 6Hz,2H), 4.48 (t, *J*=6Hz, 6Hz,2H), 6.61 (s. 1H), 7.13 (t, *J*=8.4Hz, 6.8Hz,1H), 7.31 (d, *J*=5.6Hz, 1H), 7.56 (d, *J*=5.6Hz, 1H). ^{13}C-NMR (101MHz, DMSO-d_6, ppm) δ: 167.3, 153.1, 147.3, 137.6, 127.8, 126.7, 126.6, 126.1, 112.9, 63.6, 54.9, 54.9, 53.8, 53.0, 46.1, 17.6, 18.7. HR-MS, m/z calc. for $C_{18}H_{25}N_3O$ $(M)^+$ 300.2978 found 301.2694$([M]+H)^+$.

4,8-dimethyl-2-(quinolin-8-ylmethoxy) quinoline (12).

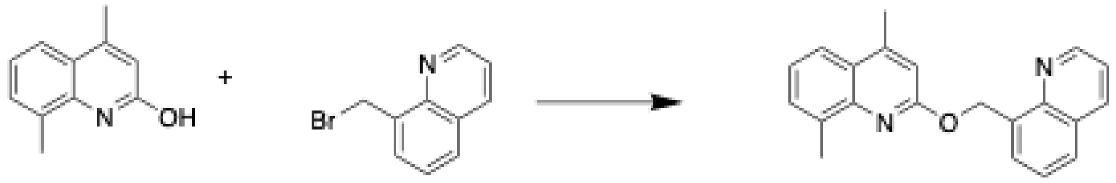

12

4,8-dimethylquinolin-2-ol (100 mg, 57.7 mmol) and 8-(bromomethyl)quinoline (192.3 mg, 86.6 mmol) was used to afford the product follow the same condition of **8**. (46 mg, 14.6 mmol, yield=25.6%). ^{1}H-NMR (400MHZ, Chloroform-*d,* ppm) δ: 2.51 (s, 3H), 2.58 (s, 3H), 6.24 (s, 2H), 6.79 (s, 1H), 7.18 (t, *J*=7.2Hz, 8.8Hz, 1H), 7.33 (d, *J*= 4.4Hz, 1H), 7.51 (t, *J*=6.8Hz, 6.4Hz,1H), 7.43 (t, *J*=7.2Hz, 8Hz,1H), 7.62 (d, *J*=8Hz, 1H), 7.65 (d, *J*=9.2Hz, 1H), 8.04(d, *J*=7.2Hz, 1H), 8.92 (d, *J*= 4.4Hz, 1H). ^{13}C-NMR (101MHz, DMSO-d_6, ppm) δ: 167.3, 153.1, 153.2, 148.3, 147.3, 137.6, 130.9, 129.6, 127.9, 127.8, 127.8, 126.7, 126.6, 126.6, 126.5, 126.0, 122.1, 112.9, 69.5, 18.7, 17.6. HR-MS, m/z calc. for $C_{21}H_{17}N_2O$ $(M)^+$ 315.1439 found 316.1422 $([M]+H)^+$.

1-(3-(4,8-dimethyl-2-(2-(pyrrolidin-1-yl)ethoxy)quinolin-7-yl)thiophen-2-yl)ethan-1-one (1.1).

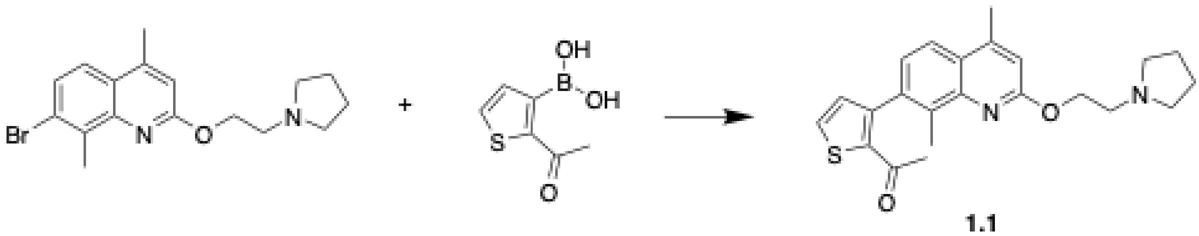

1.1

Compound **1** (100 mg, 28.6 mmol), and 2-acetylthiophen-3-yl) boronic acid (72.9 mg, 42.9 mmol) were used to afford the compound **1.1** (25 mg, 6.3 mmol, yield=22%). ^{1}H-NMR (400MHZ, DMSO-d_6, ppm) δ:1.86-1.90 (m, 4H), 2.27 (s, 3H), 2.31 (s, 3H), 3.8 (s,3H), 2.85 (t, *J*=8 Hz, 8.4Hz, 2H), 2.97-3.01 (m, 4H), 4.20 (t, *J*=6.4 Hz, 8.4Hz, 2H), 6.91 (s, 1H), 7.40 (d, *J*=6.4 Hz, 1H), 7.51 (d, *J*=8.4 Hz, 1H), 7.65 (d, *J*=6.4 Hz, 1H), 8.32 (d, *J*=8.4 Hz, 1H). ^{13}C-NMR (101MHZ, DMSO-d_6, ppm) δ: 191.4, 167.3, 147.3, 147.2, 145.8, 132.7, 129.2, 128.8, 127.6, 127.4, 126.7, 127.4, 126.7, 123.9, 112.9, 62.6, 55.0, 54.7, 54.6, 27.4, 23.4, 23.4, 18.7. HR-MS, m/z calc. for $C_{23}H_{26}N_2O_2S(M)^+$ 396.1779 found 396.1812 $([M]+H)^+$

7-(benzo[*b*]thiophen-3-yl)-4,8-dimethyl-2-(2-(pyrrolidin-1-yl)ethoxy)quinoline (1.2).

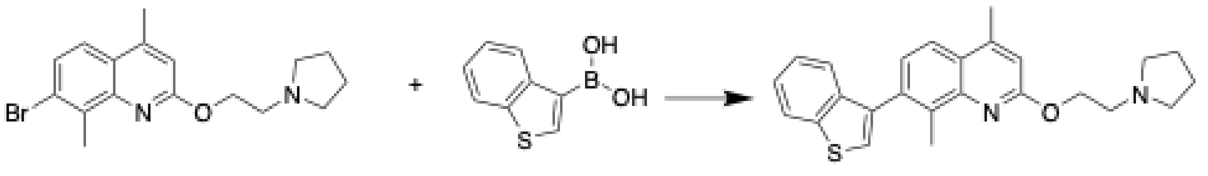

1.2

Compound **1** and benzo[*b*]thiophen-3-ylboronic acid (76.4 mg, 42.9 mmol) were used to obtain the compound **1.2** (31 mg, 7.7 mmol, yield=27%). ^{1}H-NMR (400MHZ, Chloroform-*d,* ppm) δ: 1.90-1.95 (m,4H), 2.56 (s, 3H), 2.69 (s,3H), 2.95 (t, *J*=6.8Hz, 8Hz, 4H), 3.23 (t, *J*=5.6Hz, 5.2Hz, 2H), 4.80 (t, *J*=5.6Hz, 5.2Hz, 2H), 6.87 (s,1H), 7.34 (t, *J*=6Hz, 6Hz, 1H),7.37 (s, 1H), 7.39 (t, *J*=6Hz, 6Hz, 1H), 7.47 (d, *J*=8.4Hz, 1H), 7.82 (d, *J*=8.4Hz, 1H), 7.96 (d, *J*=7.6Hz, 1H). ^{13}C-NMR (101MHz, DMSO-d_6, ppm) δ: 164.3, 153.1, 147.3, 147.1, 138.1, 137.4, 135.0, 130.7, 130.2, 127.8, 126.7, 126.0, 125.4, 125.3, 122.3, 121.7, 112.9, 62.6, 54.9, 54.7, 54.8, 23.4, 23.4, 18.9, 18.7. HR-MS, m/z calc. for $C_{25}H_{26}N_2OS$ (M)$^+$ 403.1928 found 404.1863 ([M]+H)$^+$.

4,8-dimethyl-7-phenethyl-2-(2-(pyrrolidin-1-yl)ethoxy)quinoline (1.3).

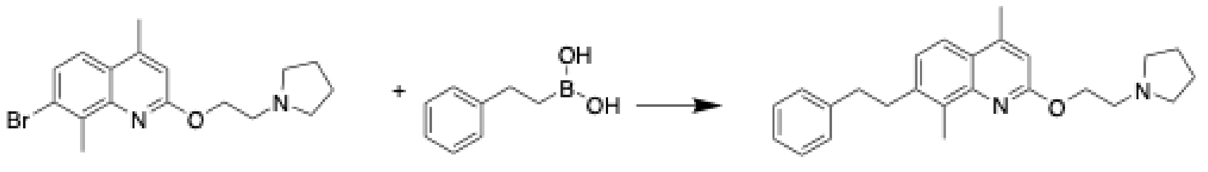

1.3

Compound **1** and phenethylboronic acid (64.3 mg, 42.9 mmol) were used to afford the compound **1.3** (42 mg, 11.2 mmol, yield=39.2%). ^{1}H-NMR (400MHZ, Chloroform-*d,* ppm) δ: 1.97-2.01 (m, 4H), 2.67 (s, 3H), 2.76 (s, 3H), 2.95-3.02 (m, 4H), 3.10 (t, *J*=4.8Hz, 8Hz, 2H), 3.25 (t, *J*=5.6Hz, 6Hz, 2H), 5.12 (t, *J*=4.8Hz, 4.8Hz, 2H), 5.32 (t, *J*=4.8Hz, 4.8Hz, 2H), 6.82 (s, 1 H) 7.57 (d, *J*=4.2Hz, 1H), 7.68 (t, *J*=4.8Hz, 6Hz, 1H), 7.71 (t, *J*=4.8Hz, 6Hz, 2H), 8.01 (d, *J*=8Hz, 2H), 8.23 (d, *J*=9.6Hz, 1H). ^{13}C-NMR (101MHz, DMSO-d_6, ppm) δ: 162.4, 146.3, 145.3, 139.0, 136.5, 129.0, 128.7, 128.7, 128.3, 127.9, 127.8, 127.4, 126.9, 126.7, 112.9, 62.6, 54.0, 54.8, 54.7, 35.8, 35.7, 23.4, 23.3, 18.6. HR-MS, m/z calc. for $C_{25}H_{30}N_2O$ (M)$^+$ 375.3723 found 376.2720 ([M]+H)$^+$.

7-(furan-3-yl)-4,8-dimethyl-2-(2-(pyrrolidin-1-yl)ethoxy)quinoline (1.4).

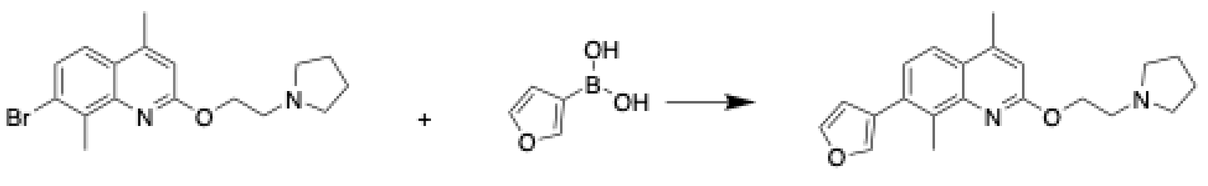

1.4

Compound **1** and furan-3-ylboronic acid (48 mg, 42.9 mmol) were used to afford the compound **1.4** (56 mg, 16.6 mmol, yield=58%). ^{1}H-NMR (400MHZ, DMSO-d_6, ppm) δ: 1.85-1.89 (m, 4H), 2.25 (s, 3H), 2.45 (s, 3H), 2.85 (t, *J*=8.4Hz, 5.6Hz, 2H), 2.97-3.00 (m, 4H), 4.14 (t, *J*=8Hz, 5.6Hz, 2H) 6.45 (d, *J*=4Hz, 1H), 6.80 (s, 1H), 7.3 (d, *J*=8.4Hz, 1H), 7.69 (d, *J*=4.8Hz, 1H), 8.12(s, 1H), 8.28 (d, *J*=8.4Hz, 1H). ^{13}C-NMR (101MHZ, DMSO-d_6, ppm) δ: 167.1, 147.3, 147.2, 143.1, 141.1, 132.7, 127.8, 127.4, 125.7, 126.6, 123.9, 112.9, 109.8, 62.6, 54.9, 54.7, 54.6, 23.4, 23.3, 18.7. HR-MS, m/z calc. for $C_{21}H_{24}N_2O_2$ $(M)^+$ 337.1904 found 338.1927 $([M]+H)^+$.

7-(furan-2-yl)-4,8-dimethyl-2-(2-(pyrrolidin-1-yl)ethoxy)quinoline (1.5).

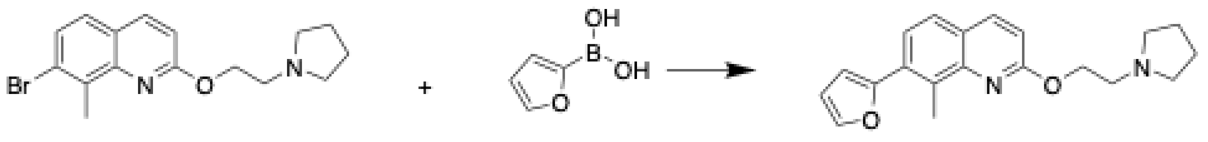

1.5

Compound **1** and furan-2-ylboronic acid (48 mg, 42.9 mmol) were used to afford the compound **1.5** (46 mg, 13.7 mmol, yield=47.8). ^{1}H-NMR (400MHZ, DMSO-d_6, ppm) δ: 1.86-1.90 (m, 4H), 2.29 (s, 3H), 2.44(s, 3H), 2.85 (t, *J*=8.4Hz, 8.4Hz, 2H), 2.97-3.01 (m, 4H), 4.15 (t, *J*=6Hz, 6.4Hz, 2H), 6.56 (t, *J*=4.0Hz, 4.8Hz, 1H), 6.81 (s, 1H), 6.85 (d, *J*=5.6Hz, 1H), 7.82 (d, *J*=6Hz, 2H), 8.17 (d, *J*=8.4Hz, 1H), 8.40 (d, *J*=5.6Hz, 2H). ^{13}C-NMR (101MHZ, DMSO-d_6, ppm) δ: 167.3, 154.1, 147.3, 147.4, 142.3, 128.2, 127.8, 127.4, 126.7, 126.6, 112.9, 111.4, 104.8, 62.6, 55.0, 54.7, 54.7, 23.4, 23.4, 18.7. HR-MS, m/z calc. for $C_{21}H_{24}N_2O_2$ $(M)^+$ 348.2070 found 348.2102 $([M]+H)^+$.

4,8-dimethyl-7-phenyl-2-(2-(pyrrolidin-1-yl)ethoxy)quinoline (1.6).

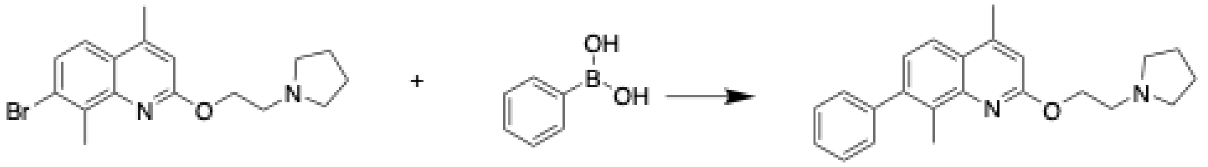

1.6

Compound **1** and phenylboronic acid (52.3 mg, 42.9 mmol) were used to afford the title compound (52 mg, 15 mmol, yield=52.5%). ^{1}H-NMR (400MHZ, Chloroform-*d,* ppm) δ: 1.88-1.92 (m, 4H), 2.65 (s, 3H), 2.67 (s, 3H), 2.81 (t, *J*=8Hz, 8.4Hz, 4H), 3.10 (t, *J*=4.8Hz, 8Hz, 2H), 4.75 (t, *J*=4.8Hz, 4.8Hz, 2H), 6.84 (s, 1H), 7.34 (d, *J*=4.2Hz, 1H), 7.39 (t, *J*=4.8Hz, 6Hz, 1H), 7.43 (t, *J*=4.8Hz, 6Hz, 2H), 7.48 (d, *J*=8Hz, 2H), 7.79 (d, *J*=9.6Hz, 1H). ^{13}C-NMR (101MHz, DMSO-d_6, ppm) δ: 167.3, 153.1, 147.3, 139.5, 137.6, 130.7, 130.1, 128.0, 128.8, 128.7, 128.6, 128.6, 126.7, 126.1, 112.9, 62.7, 55.1, 55.0, 55.0, 23.4, 23.3, 19.1, 18.3. HR-MS, m/z calc. for $C_{23}H_{26}N_2O$ $(M)^+$ 347.2170 found 347.2988 $([M]+H)^+$.

4,8-dimethyl-7-(naphthalen-2-yl)-2-(2-(pyrrolidin-1-yl)ethoxy)quinoline (1.7).

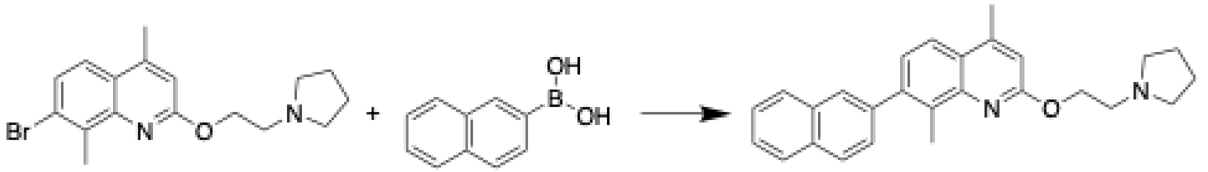

1.7

Compound **1** and naphthalen-2-ylboronic acid (73.8 mg, 42.9 mmol) were used to afford the compound **1.7** (70 mg, 17.7 mmol, yield=61.7%). ^{1}H-NMR (400MHZ, DMSO-d_6, ppm) δ: 1.88-1.92 (m, 4H), 2.26 (s, 3H), 2.52 (s, 3H), 2.87 (t, *J*=8.4Hz, 8Hz, 2H), 3.01-3.05 (m, 4H), 4.16 (t, *J*=8Hz, 5.6Hz, 2H), 6.65 (s, 1H), 7.55 (s, 1H), 7.57 (s, 1H), 7.70 (d, *J*=1.2Hz,1H), 7.72 (d, *J*=1.2Hz,1H), 7.87 (d, *J*=8.4Hz, 2H), 7.92 (t, *J*=8Hz, 5.6Hz, 2H), 8.31 (d, *J*=5.6Hz, 1H), 8.51 (t, *J*=6Hz, 5.6Hz, 1H). ^{13}C-NMR (101MHZ, DMSO-d_6, ppm) δ: 167.1, 147.3, 147.2,140.3, 139.2, 133.7, 133.6, 128.5, 128.4, 127.7, 127.3, 126.7, 126.6, 126.6, 126.5, 126.5, 125.7, 125.6, 124.5, 112.9, 62.6, 55.0, 54.7, 54.7, 23.4, 23.3, 18.7. HR-MS, m/z calc. for $C_{27}H_{28}N_2O$ $(M)^+$ 397.2852 found 398.1021 $([M]+H)^+$.

4',8'-dimethyl-2'-(2-(pyrrolidin-1-yl) ethoxy)-3,7'-biquinoline. (1.8)

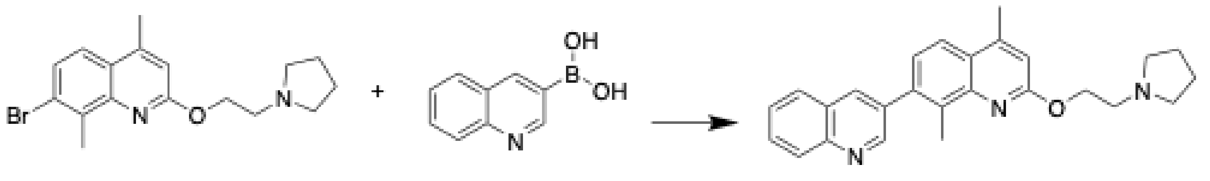

1.8

Compound **1** and quinolin-3-ylboronic acid (74.2 mg, 42.9 mmol) were used to afford the compound **1.8** (65 mg, 16.4 mmol, yield=57.2%). ^{1}H-NMR (400MHZ, DMSO-d_6, ppm) δ: 1.86-1.90 (m, 4H), 2.23 (s, 3H), 2.52 (s, 3H), 2.87 (t, *J*=6.4Hz, 8Hz, 2H), 2.98-3.02 (m, 4H), 4.19(t, *J*=8Hz, 8.4Hz, 2H), 6.83 (s, 1H), 7.66 (t, *J*=8.4Hz, 8Hz 2H), 7.93 (d, *J*=8Hz,2H), 8.19 (d, *J*=8.4Hz, 1H), 8.33 (d, *J*=8Hz, 1H), 8.7 (s, 1H), 9.4 (d, *J*= 5.6Hz, 1H). ^{13}C-NMR (101MHZ, DMSO-d_6, ppm) δ: 167.3, 139.0, 148.1, 147.3, 147.2, 139.2, 133.9, 129.1, 129.0, 128.9, 128.1, 127.4, 127.2, 126.7, 126.6, 126.5, 125.7, 112.9, 62.6, 55.1, 54.7, 54.7, 23.4, 23.4, 18.7. HR-MS, m/z calc. for $C_{26}H_{27}N_3O$ $(M)^+$ 398.2227 found 399.2258 $([M]+H)^+$.

4,8-dimethyl-7-(pyridin-4-yl)-2-(2-(pyrrolidin-1-yl)ethoxy)quinoline (1.9)

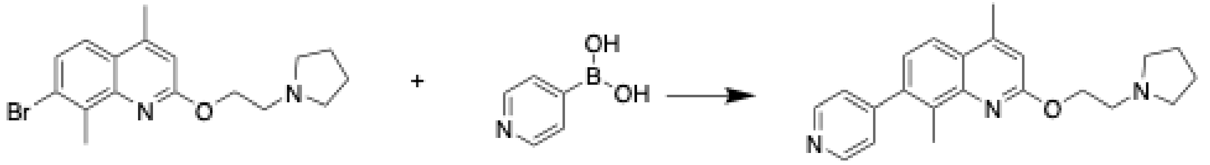

1.9

Compound **1** and pyridin-4-ylboronic acid (52.7 mg, 42.9 mmol) were used to afford compound **1.9** (60 mg, 17.2 mmol, yield=60.1%). ^{1}H-NMR (400MHZ, DMSO-d_6, ppm) δ: 1.71-1.74 (m, 4H), 2.50-2.51 (m, 4H), 2.58 (s, 3H), 2.65 (s,3H), 2.98 (t, *J*=4.8 Hz, 6.4Hz, 2H), 4.59 (t, *J*=6Hz, 5.6Hz, 2H), 6.96 (s, 1H), 7.11 (d, *J*=4.4Hz, 1H), 7.33 (d, *J*=8.4Hz, 1H), 7.46 (d, *J*=6Hz, 2H), 7.91 (d, *J*=8.4Hz, 1H), 8.68 (d, *J*=5.6Hz, 2H). ^{13}C-NMR (101MHZ, DMSO-d_6, ppm) δ: 161.1, 150.1, 149.6, 148.2, 145.1, 139.6, 132.2, 125.2, 124.9, 124.8, 122.3, 113.2, 64.0, 54.4, 54.3, 49.6, 23.5, 18.8, 15.4. HR-MS, m/z calc. for $C_{22}H_{25}N_3O$ $(M)^+$ 348.2070 found 348.2102 $([M]+H)^+$.

1-(3-(2-(2-(dimethylamino)ethoxy)-4,8-dimethylquinolin-7-yl)thiophen-2-yl)ethan-1-one (3.1).

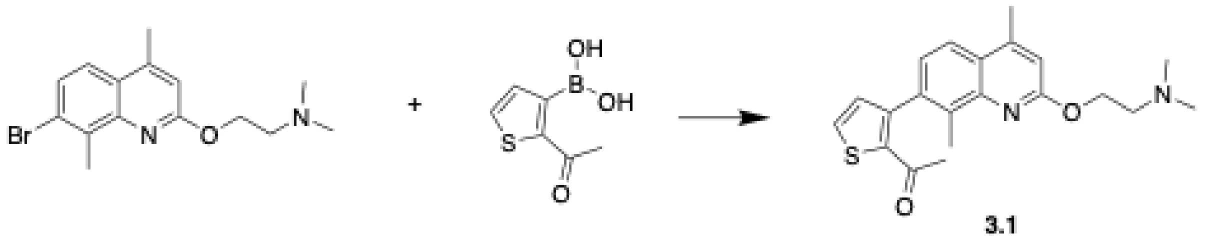

3.1

Compounf **3** and 2-acetylthiophen-3-yl)boronic acid (78.7 mg, 42.9 mmol) were used to afford the compound **3.1** (48 mg, 13 mmol, yield=42.1%). ^{1}H-NMR (400MHZ, DMSO-d_6, ppm) δ: 2.27 (s,3H), 2.30 (s, 6H), 2.35 (s, 3H), 2.42 (s, 3H), 2.82 (t, *J*=5.6Hz, 6Hz, 2H), 4.14 (t, *J*=5.6Hz, 5.6Hz, 2H), 6.88 (s, 1H), 7.62 (d, *J*=4.8Hz, 1H), 7.52(d, *J*=4.8Hz, 1H), 7.60 (d, *J*=5.6Hz, 1H), 8.34 (d, *J*=8.4Hz, 1H). ^{13}C-NMR (101MHZ, DMSO-d_6, ppm) δ: 191.4, 167.3, 147.3, 147.2, 145.8, 132.7, 129.2, 128.8, 127.6, 127.4, 126.7, 126.6, 123.9, 112.9, 62.6, 58.2, 45.6, 45.5, 27.4, 18.7. HR-MS, m/z calc. for $C_{21}H_{24}N_2O_2S(M)^+$ 369.1631 found 370.1662 ($[M]+H)^+$.

2-((7-(benzo[*b*]thiophen-3-yl)-4,8-dimethylquinolin-2-yl)oxy)-*N*,*N*-dimethylethan-1-amine (3.2).

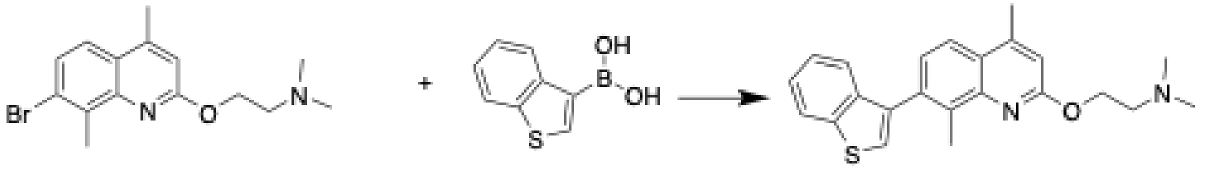

3.2

Compound **3** and benzo[*b*]thiophen-3-ylboronic acid (82.5 mg, 46.3 mmol) were used to obtain the title compound (25 mg, 6.6 mmol, yield=21.4%). ^{1}H-NMR (400MHZ, DMSO-d_6, ppm) δ:2.23 (s, 3H), 2.30 (s, 6H), 2.36(s, 3H), 2.84 (t, *J*=5.6Hz, 6Hz, 2H), 4.37 (t, *J*=5.6Hz, 6Hz, 2H), 6.83 (s, 1H), 7.29 (t, *J*=6Hz, 6Hz, 2H), 7.34(d, *J*=8.4Hz,1H), 7.89 (d, *J*=8.4Hz, 1H), 8.10 (d, *J*=6.0Hz, 1H), 8.14(d, *J*=8.4Hz, 1H), 8.43 (s, 1H). ^{13}C-NMR (101MHZ, DMSO-d_6, ppm) δ:167.0, 147.1, 146.9, 138.1, 137.1, 132.5, 129.9, 127.4, 126.7, 127.6, 125.4, 125.3, 123.7, 122.3, 121.7, 112.9, 62.6, 58.2, 45.6, 45.6, 18.7. HR-MS, m/z calc. for $C_{23}H_{24}N_2OS$ $(M)^+$ 377.1682 found 378.1708 ($[M]+H)^+$.

2-((4,8-dimethyl-7-phenethylquinolin-2-yl)oxy)-*N*,*N*-dimethylethan-1-amine (3.3).

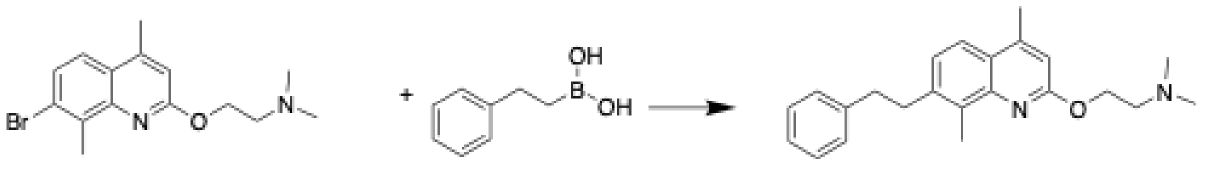

3.3

Compound **3** and phenethylboronic acid (69.4 mg, 46.3 mmol) were used to afford the compound **3.3** (28 mg, 8.5mol, yield=25.9%). ^{1}H-NMR (400MHZ, CD_3OD, ppm) δ: 2.23 (s, 6H), 2.41 (s, 3H), 2.52 (s, 3H), 2.95 (t, *J*=6Hz, 5.6Hz, 2H), 3.02 (t, *J*=6Hz, 5.6Hz, 2H), 3.15 (t, *J*=6Hz, 5.6Hz, 2H), 4.82 (t, *J*=5.6Hz, 5.6Hz, 2H), 6.56 (s, 1 H), 7.34 (d, *J*=4.2Hz, 1H), 7.53 (t, *J*=4.8Hz, 6Hz, 1H), 7.72 (t, *J*=4.8Hz, 6Hz, 2H), 8.01 (d, *J*=8Hz, 2H), 8.23 (d, *J*=9.6Hz, 1H). ^{13}C-NMR (101MHZ, DMSO-d_6, ppm) δ: 167.3, 147.3, 147.2, 143.0, 136.5, 128.9, 128.7, 128.7, 127.5, 128.4, 128.0, 127.4, 126.7, 126.6, 112.9, 62.3, 58.2, 45.6, 45.6, 36.9, 36.8, 18.7. HR-MS, m/z calc. for $C_{19}H_{22}N_2O_2$ $(M)^+$ 311.2301 found 312.1778 $([M]+H)^+$.

2-((7-(furan-3-yl)-4,8-dimethylquinolin-2-yl)oxy)-*N*,*N*-dimethylethan-1-amine (3.4).

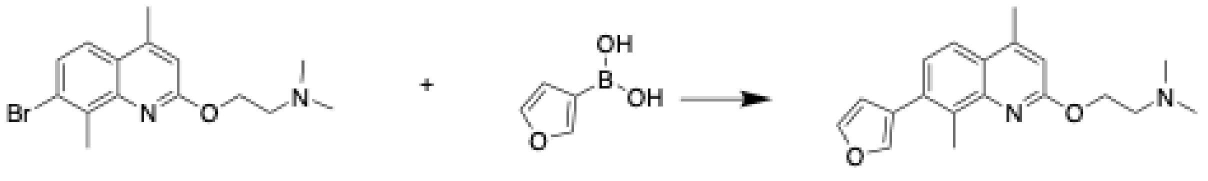

3.4

Compound **3** and furan-3-ylboronic acid (51.8 mg, 46.3 mmol) were used to afford the compound **3.4** (42 mg, 13.5 mmol, yield=43.7%). ^{1}H-NMR (400MHZ, DMSO-d_6, ppm) δ: 2.29 (s, 6H), 2.62, (s, 3H), 2.72 (s, 3H), 2.77 (t, *J*=5.6Hz, 5.6Hz, 2H), 4.57 (t, *J*=6Hz, 6.4Hz, 2H), 6.88 (s, 1H), 7.45 (d, *J*=8.4Hz, 1H), 7.82 (d, *J*=2Hz, 1H), 7.83 (s, 1H), 7.99 (d, *J*=2Hz, 1H),8.14 (d, *J*=2Hz, 1H). ^{13}C-NMR (101MHZ, DMSO-d_6, ppm) δ:161.3, 153.1, 147.3, 143.1, 141.1, 135.0, 130.7, 127.9, 127.8, 126.7, 126.0, 112.9, 109.8, 62.6, 58.2, 45.6, 45.7, 18.9, 18.7. HR-MS, m/z calc. for $C_{19}H_{22}N_2O_2$ $(M)^+$ 311.1749 found 312.1781 $([M]+H)^+$.

2-((7-(furan-2-yl)-4,8-dimethylquinolin-2-yl)oxy)-*N*,*N*-dimethylethan-1-amine (3.5).

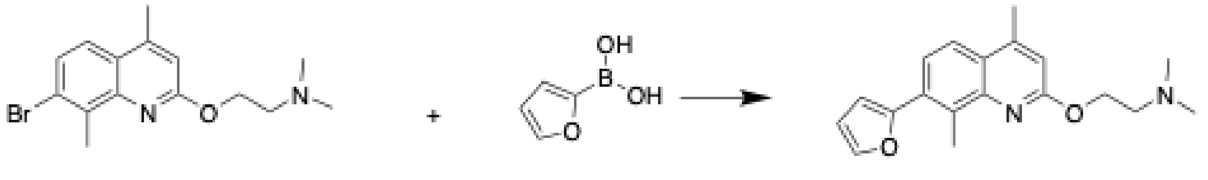

3.5

Compound **3** and furan-2-ylboronic acid (51.8 mg, 46.3 mmol) were used to afford the compound **3.5** (30 mg, 9.7 mmol, yield=31.4%).^{1}H-NMR (400MHZ, DMSO-d_6, ppm) δ: 2.28 (s, 3H), 2.31 (s, 6H), 2.42(s, 3H), 2.80 (t, *J*=8.2Hz, 6.4Hz, 2H), 4.15 (t, *J*=6Hz, 6.4Hz, 2H), 6.56 (t, *J*=4.0Hz, 4.8Hz, 1H),6.85 (s, 1H), 6.89 (d, *J*=5.6Hz, 1H), 7.84 (d, *J*=6Hz, 2H), 8.20 (d, *J*=8.4Hz, 1H), 8.41(d, *J*=5.6Hz, 2H). ^{13}C-NMR (101MHZ, DMSO-d_6, ppm) δ: 161.3, 154.1, 147.3, 147.4, 142.3, 128.2, 127.8, 127.4, 126.7, 126.6, 112.9, 111.4, 104.8, 62.6, 58.2, 45.6, 45.6, 18.7. HR-MS, m/z calc. for $C_{19}H_{22}N_2O_2$ $(M)^+$ 311.2703 found 311.8955 $([M]+H)^+$.

2-((4,8-dimethyl-7-phenylquinolin-2-yl)oxy)-*N*,*N*-dimethylethan-1-amine (3.6).

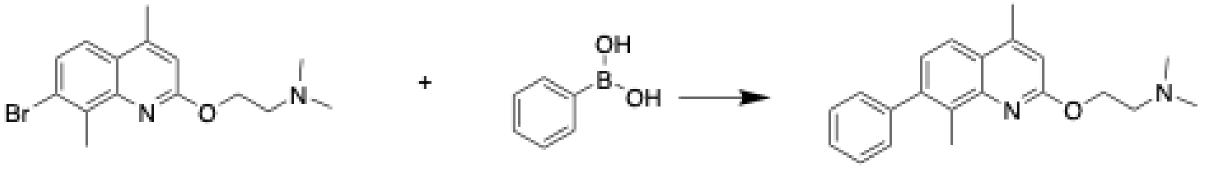

3.6

Compound **3** and phenylboronic acid (56.4mg, 42.9mmol) were used to afford the compound **3.6** (38 mg, 11.8 mmol, yield=38.2%). ^{1}H-NMR (400MHZ, CD_3OD, ppm) δ: 2.44 (s, 6H), 2.60 (s, 3H), 2.65 (s, 3H), 2.94 (t, *J*=6Hz, 5.6Hz, 2H), 4.66 (t, *J*=5.6Hz, 5.6Hz, 2H), 6.83 (s, 1H), 7.29 (d, *J*=4.2Hz, 1H), 7.37 (t, *J*=4.8Hz, 6Hz, 1H), 7.39 (t, *J*=4.8Hz, 6Hz, 2H), 7.45 (d, *J*=8Hz, 2H), 7.82 (d, *J*=8.4Hz, 1H). ^{13}C-NMR (101MHZ, DMSO-d_6, ppm) δ: 167.3, 153.1, 147.3, 139.5, 137.6, 130.7, 130.0, 128.9, 128.9, 128.8, 128.6, 128.5, 126.7, 112.9, 62.6, 58.2, 45.6, 45.5, 18.9, 18.7. HR-MS, m/z calc. for $C_{19}H_{22}N_2O_2$ $(M)^+$ 311.1955 found 311.8577 $([M]+H)^+$.

2-((4,8-dimethyl-7-(naphthalen-2-yl)quinolin-2-yl)oxy)-*N*,*N*-dimethylethan-1-amine (3.7).

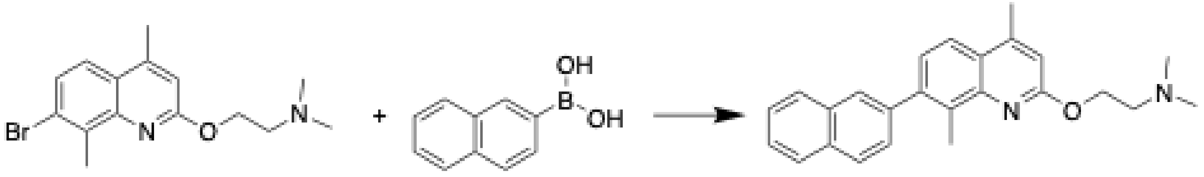

3.7

Compound **3** and naphthalen-2-ylboronic acid (79.6 mg, 46.3 mmol) were used to afford the compound **3.7** (56 mg, 15.1 mmol, yield=48.9%). ^{1}H-NMR (400MHZ, DMSO-d_6, ppm) δ: 2.24 (s, 6H), 2.3 (s, 3H), 2.59 (s, 3H), 2.83 (t, *J*=6Hz, 5.6Hz, 2H), 4.12 (t, *J*=5.6Hz, 6Hz, 2H), 6.88 (s, 1H), 7.56 (d, *J*=8.4Hz, 6Hz , 2H), 7.71 (d, *J*=8.8Hz, 1H), 7.69 (d, *J*=8Hz, 1H), 7.78 (d, *J*=8.4Hz, 1H), 7.92 (t, *J*=5.6Hz, 6Hz, 2H), 8.30 (d, *J*=8.8Hz, 1H), 8.5(s, 1H). ^{13}C-NMR (101MHZ, DMSO-d_6, ppm) δ:163.3, 147.3, 147.2, 140.2, 139.2, 133.7, 133.6, 128.5, 127.4, 127.7, 127.4, 126.7, 126.6, 126.5, 126.5, 126.7, 126.6, 124.5, 112.9, 62.6, 58.2, 45.6, 45.6, 18.7. HR-MS, m/z calc. for $C_{25}H_{26}N_2O$ $(M)^+$ 371.2733 found 372.1021 $([M]+H)^+$.

2-((4',8'-dimethyl-[3,7'-biquinolin]-2'-yl) oxy)-*N*,*N*-dimethylethan-1-amine (3.8).

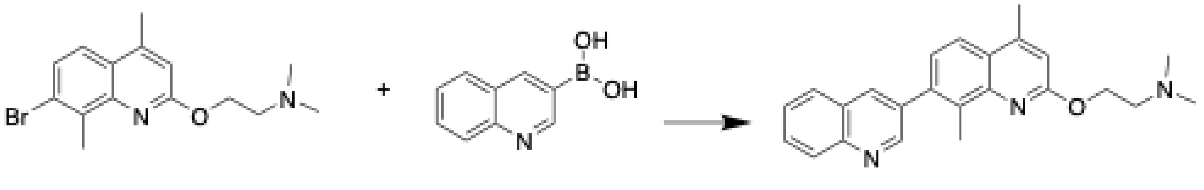

3.8

The title compounds were obtained with **3** and quinolin-3-ylboronic acid (80.1mg, 46.3mmol) followed by the general protocol. (53mg, 14.3mmol, yield=46.3%). ^{1}H-NMR (400MHZ, DMSO-d_6, ppm) δ:2.23 (s, 6H), 2.58 (s, 3H), 2.59 (s, 3H), 2.70 (t, *J*=5.6Hz, 6Hz, 2H), 4.53 (t, *J*=5.6Hz, 6Hz, 2H), 6.87 (s, 1H), 7.39 (d, *J*=8.4Hz, 1H), 7.63 (t, *J*=7.2Hz, 7.6Hz, 1H), 7.78 (t, *J*=6.8Hz, 8Hz, 1H), 7.84 (d, *J*=8.8Hz, 1H), 8.01 (d, *J*=8Hz, 1H), 8.09 (d, *J*=8.4Hz, 1H), 8.37 (s, 1H), 8.96 (d, *J*=2Hz, 1H). ^{13}C-NMR (101MHZ, DMSO-d_6, ppm) δ:161.0, 151.7, 147.9, 147.0, 145.2, 138.5, 135.9, 134.9, 132.8, 130.1, 129.2, 128.7, 127.8, 127.4, 126.0, 124.6, 122.1, 113.0, 63.2, 57.8, 45.8, 18.7, 15.5. HR-MS, m/z calc. for $C_{24}H_{25}N_3O$ $(M)^+$ 371.2 found 372.2 $([M]+H)^+$.

2-((4,8-dimethyl-7-(pyridin-4-yl)quinolin-2-yl)oxy)-*N*,*N*-dimethylethan-1-amine (3.9).

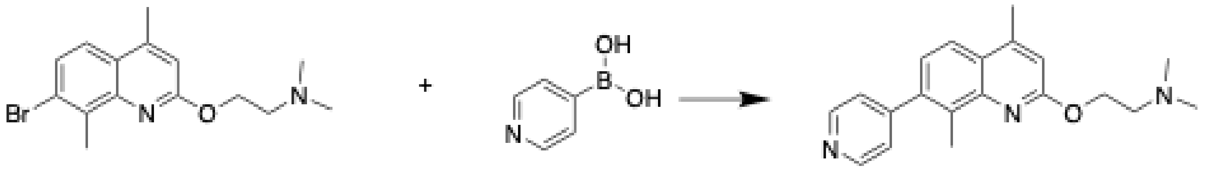

3.9

Compound **3** and pyridin-4-ylboronic acid (56.9 mg, 46.3 mmol) were used to afford the product **3.9** (54 mg, 16.8 mmol, yield=54.3%). ^{1}H-NMR (400MHZ, DMSO-d_6, ppm) δ: 2.23 (s, 3H), 2.29 (s, 6H), 2.45(s, 3H), 2.82 (t, *J*=6.4Hz, 4Hz, 2H), 4.18 (t, *J*=6Hz, 6.4Hz, 2H), 6.92 (s, 1H), 7.24 (d, *J*=8.4Hz, 1H), 7.93 (d, *J*=6Hz, 2H), 8.36 (d, *J*=8.4Hz, 1H),8.44 (d, *J*=5.6Hz, 2H). ^{13}C-NMR (101MHZ, DMSO-d_6, ppm) δ: 167.3, 150.0, 149.8, 147.3, 147.2, 147.0, 139.2, 127.4, 126.7, 126.6, 125.7, 123.1, 123.1, 112.9, 62.6, 58.2, 45.6, 46.6, 18.7. HR-MS, m/z calc. for $C_{20}H_{23}N_2OS$ (M)$^+$ 372.2070 found 373.2102 ([M]+H)$^+$.

7-bromo-2-(2-(pyrrolidin-1-yl)ethoxy)quinoline (1.10).

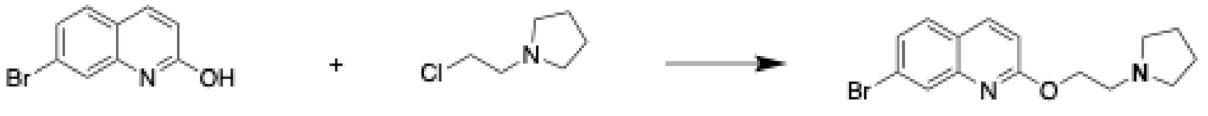

1.10

7-bromoquinolin-2-ol (200 mg, 89.2 mmol), 1-(2-chloroethyl)pyrrolidine (99.3 mg, 107 mmol) and K_2CO_3 (246.6 mg, 178.4 mmol) were mixed in 15mL DMF in a microwave-proof glass tube. The mixture was heated for 2 hrs at 170°C in a microwave synthesis. Upon cooling, the reaction was extracted with EtOAc and H_2O. $MgSO_4$ was used to dry the separated organic layer. The title compound was purified by flash column chromatography. ^{1}H-NMR (400MHZ, CD_3OD, ppm) δ: 1.96-1.87 (m, 4H), 2.75 (t, *J*= 6.8Hz, 6.4Hz, 3H), 3.01 (t, *J*= 5.6Hz, 6Hz, 2H), 4.52 (t, *J*= 5.6Hz, 6Hz, 2H), 7.09 (d, *J*= 4.8Hz, 1H), 7.51 (d, *J*= 8Hz, 1H), 7.89 (d, *J*=8Hz, 1H), 8.00 (d, *J*= 4Hz, 1H), 8.95 (s, 1H). ^{13}C-NMR (101MHZ, DMSO-d_6, ppm) δ: 163.5, 147.3, 130.2, 128.3, 127.6, 126.9, 126.7, 120.0, 118.2, 62.6, 55.0, 54.7, 54.7, 23.4, 23.4. HR-MS, m/z calc. for $C_{15}H_{17}BrN_2O$ (M)$^+$ 321.0723 found 321.5149 ([M]+H)$^+$.

7-phenyl-2-(2-(pyrrolidin-1-yl)ethoxy)quinoline (1.11).

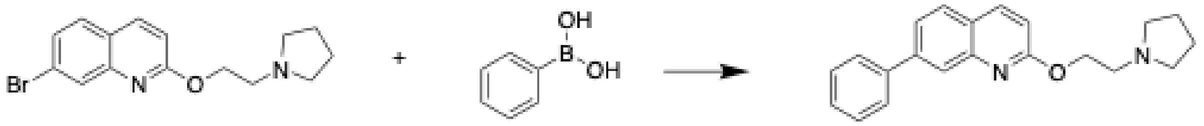

1.11

1.10 (100 mg, 31.1 mmol), phenylboronic acid (60 mg, 37.4 mmol), $CsCO_3$(121.6 mg, 37.32 mmol), and $Pd(PPh_3)_4$ (10%mol, 3.11 mmol) was mixed in the round bottom flask, followed by addition of 5mL mixture of toluene and MeOH (4:1). The reaction was heated to reflux for overnight. Upon cooling, EtOAc and H_2O were used for extraction. The organic layer was dried through $MgSO_4$ and evaporated. The final compound was obtained by purification using flash column chromatography (68 mg, 21.4 mmol, yield=68.8%).^{1}H-NMR (400MHZ, CD_3OD, ppm) δ: 1.81-1.85 (m, 4H), 2.85 (t, *J*= 8.4HZ, 8.4Hz, 2H), 3.01-3.05 (m, 4H), 4.55 (t, *J*= 8.4HZ, 8.4Hz, 2H), 6.78 (d, *J*= 8.4Hz, 1H), 7.44 (t, *J*= 1.2Hz, 2.4Hz, 1H), 7.50 (t, *J*= 4.8Hz, 2.4Hz, 2H), 7.71 (d, *J*= 6.4Hz, 1H), 7.91 (d, *J*= 6.4Hz, 2H), 8.07 (d, *J*= 8.4Hz, 1H), 8.32 (d, *J*= 5.6Hz, 1H),8.66 (d, *J*= 8.4Hz, 1H). ^{13}C-NMR (101MHZ, DMSO-d_6, ppm) δ: 167.3, 147.3, 147.2, 139.2, 139.1, 129.0, 128.9, 128.9, 127.3, 127.1, 127.0, 126.7, 126.6, 125.7, 112.9, 62.6, 55.0, 54.7, 54.7, 23.4, 23.4, 18.7. HR-MS, m/z calc. for $C_{21}H_{22}BN_2O$ $(M)^+$ 319.1744 found 319.3558 $([M]+H)^+$.

_N,N_-dimethyl-2-((7-phenylquinolin-2-yl)oxy)ethan-1-amine (1.12).

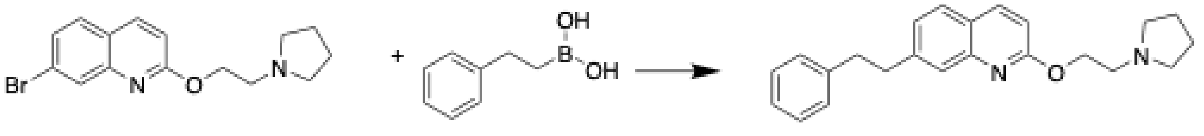

1.12

1.10 (100mg, 31.1mmol), phenethylboronic acid (56 mg, 37.3 mmol), $CsCO_3$(121.6 mg, 37.32 mmol), and $Pd(PPh_3)_4$ (10%mol, 3.11mmol) was mixed in the round bottom flask, followed by addition of 5mL mixture of toluene and MeOH (4:1). The reaction was heated to reflux for overnight. Upon cooling, EtOAc and H_2O were used for extraction. The organic layer was dried through $MgSO_4$ and evaporated. The final compound **1.12** was obtained by purification using flash column chromatography (61 mg, 17.6 mmol, yield=56.6%).^{1}H NMR (400 MHz, CD_3OD-*d4,* ppm) δ: 1.97-2.01 (m, 4H), 2.95-3.02 (m, 4H), 3.10 (t, *J*=4.8Hz, 8Hz, 2H), 3.25 (t, *J*=5.6Hz, 6Hz, 2H), 3.82 (t, *J*=4.8Hz, 4.8Hz, 2H), 5.32 (t, *J*=4.8Hz, 4.8Hz, 2H), 6.78 (d, *J*= 8.4Hz, 1H), 7.44 (t, *J*= 1.2Hz, 2.4Hz, 1H), 7.50 (t, *J*= 4.8Hz, 2.4Hz, 2H), 7.71 (d, *J*= 6.4Hz, 1H), 7.91 (d, *J*= 6.4Hz, 2H), 8.07 (d, *J*= 8.4Hz, 1H), 8.32 (d, *J*= 5.6Hz, 1H),8.66 (d, *J*= 8.4Hz, 1H). ^{13}C-NMR (101MHZ, DMSO-d_6, ppm) δ: 167.1, 147.1, 146.9, 143.0, 136.5, 128.9, 128.7, 128.7, 128.5, 128.5, 128.0, 127.1, 126.7, 126.6, 112.9, 62.5, 55.0, 54.6, 54.6, 36.9, 36.8, 23.4, 23.4, 18.7. HR-MS, m/z calc. for $C_{23}H_{26}N_2O$ $(M)^+$ 347.2423 found 348.2337 $([M]+H)^+$.

2-((7-bromoquinolin-2-yl)oxy)-*N*,*N*-dimethylethan-1-amine (3.10)

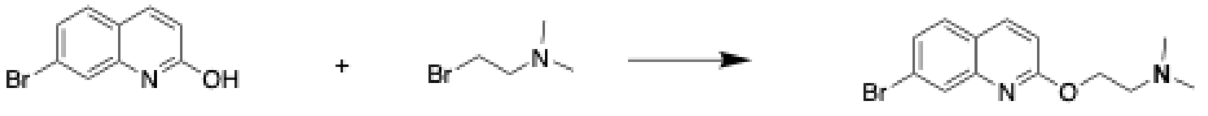

3.10

7-bromoquinolin-2-ol (200 mg, 89.2 mmol), 2-bromo-*N*,*N*-dimethylethan-1-amine (203.5 mg, 133.8 mmol)and K_2CO_3 (246.6 mg, 178.4 mmol) were mixed in 15 mL DMF in a microwave-proof glass tube. The mixture was heated for 2 hrs at 170°C in a microwave synthesis. Upon cooling, the reaction was extracted with EtOAc and H_2O. $MgSO_4$ was used to dry the separated organic layer. The title compound was purified by flash column chromatography. (102 mg, 34.5 mmol, yield=38.7%). ^{1}H-NMR (400MHZ, CD_3OD, ppm) δ: 2.41 (S, 6H), 2.88 (t, *J*= 6.4Hz, 6.4Hz, 2H), 4.50 (t, *J*= 6.4Hz, 6.4Hz, 2H), 7.09 (d, *J*= 4.8Hz, 1H), 7.51 (d, *J*= 12.8Hz, 1H), 7.89 (d, *J*=4.8Hz, 1H), 8.01 (d, *J*= 1.6Hz, 1H), 8.96 (s, 1H). ^{13}C-NMR (101MHZ, DMSO-d_6, ppm) δ: 163.5, 147.3, 130.2, 128.3, 127.6, 126.9, 126.7, 120.0, 118.2, 62.6, 58.2, 45.6, 45.7. HR-MS, m/z calc. for $C_{13}H_{15}BrN_2O$ $(M)^+$ 294.9772 found 295.2791 $([M]+H)^+$.

***N*,*N*-dimethyl-2-((7-phenylquinolin-2-yl)oxy)ethan-1-amine. (3.11)**

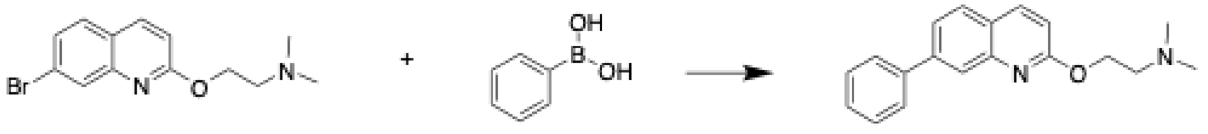

3.11

3.10 (100 mg, 33.9 mmol), phenylboronic acid (65.3 mg, 40.7 mmol), $CsCO_3$ (165.7 mg, 50.85 mmol), and $Pd(PPh_3)_4$ (10%mol, 3.39 mmol) was mixed in the round bottom flask, followed by addition of 5 mL mixture of toluene and MeOH (4:1). The reaction was heated to reflux for overnight. Upon cooling, EtOAc and H_2O were used for extraction. The organic layer was dried through $MgSO_4$ and evaporated. The compound **3.11** was obtained by purification using flash column chromatography (49 mg, 16.8 mmol, yield=49.6%).^{1}H-NMR (400MHZ, CD_3OD, ppm) δ: 2.45 (s, 6H), 2.93 (t, *J*= 6.4Hz, 65.6Hz, 2H), 4.50 (t, *J*= 6.4Hz, 6.4Hz, 2H), 7.20 (d, *J*= 6.4Hz, 1H), 7.44 (t, *J*= 1.2Hz, 2.4Hz, 1H), 7.49 (t, *J*= 4.8Hz, 2.4Hz, 2H), 7.72 (d, *J*= 6.4Hz, 1H), 7.88 (d, *J*= 6.4Hz, 2H), 8.06 (d, *J*= 8.4Hz, 1H), 8.36 (d, *J*= 5.6Hz, 1H),8.66 (d, *J*= 8.4Hz, 1H). ^{13}C-NMR (101MHZ, DMSO-d_6, ppm) δ: 167.3, 147.3, 127.2, 139.2, 129.1, 129.0, 128.9, 128.9, 127.4, 127.1, 126.7, 126.6, 125.7, 112.9, 62.6, 58.2, 45.6, 45.6, 18.7. HR-MS, m/z calc. for $C_{19}H_{20}N_2O$ $(M)^+$ 293.2133 found 294.1002 $([M]+H)^+$.

***N*,*N*-dimethyl-2-((7-phenethylquinolin-2-yl)oxy)ethan-1-amine (3.12).**

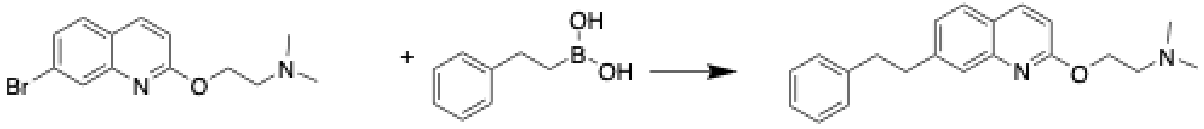

3.12

3.10 (100 mg, 33.9 mmol), phenethylboronic acid (61.1 mg, 40.7 mmol), $CsCO_3$(165.7 mg, 50.85 mmol), and $Pd(PPh_3)_4$ (10%mol, 3.39 mmol) was mixed in the round bottom flask, followed by addition of 5mLmixture of toluene and MeOH (4:1). The reaction was heated to reflux for overnight. Upon cooling, EtOAc and water were used for extraction. The organic layer was dried through $MgSO_4$ and evaporated. The final compound was purified using flash column chromatography (47 mg, 14.7 mmol, yield=43.4%).1H NMR (400 MHz, CD_3OD, ppm) δ: 2.43 (s, 6 H) 3.10 (t, *J*=4.8Hz, 8Hz, 2H), 3.25 (t, *J*=5.6Hz, 6Hz, 2H), 3.82 (t, *J*=4.8Hz, 4.8Hz, 2H), 5.32 (t, *J*=4.8Hz, 4.8Hz, 2H), 6.78 (d, *J*= 8.4Hz, 1H), 7.44 (t, *J*= 1.2Hz, 2.4Hz, 1H), 7.50 (t, *J*= 4.8Hz, 2.4Hz, 2H), 7.71 (d, *J*= 6.4Hz, 1H), 7.91 (d, *J*= 6.4Hz, 2H), 8.07 (d, *J*= 8.4Hz, 1H), 8.32 (d, *J*= 5.6Hz, 1H),8.66 (d, *J*= 8.4Hz, 1H). ^{13}C-NMR (101 MHZ, DMSO-d_6, ppm) δ: 166.2, 147.1, 146.9, 143.0, 136.5, 128.9, 128.7, 128.5, 128.5, 128.0, 127.3, 126.7, 126.6, 112.9, 62.6, 58.2, 45.6, 45.6, 36.9, 36.8, 18.7. HR-MS, m/z calc. for $C_{21}H_{24}N_2O$ $(M)^+$ 321.1776 found 322.1135 ([M]+H)

^{1}H and ^{13}C NMR spectra of Quinoline based EPI Compounds

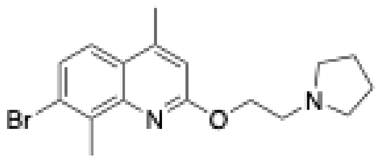

1

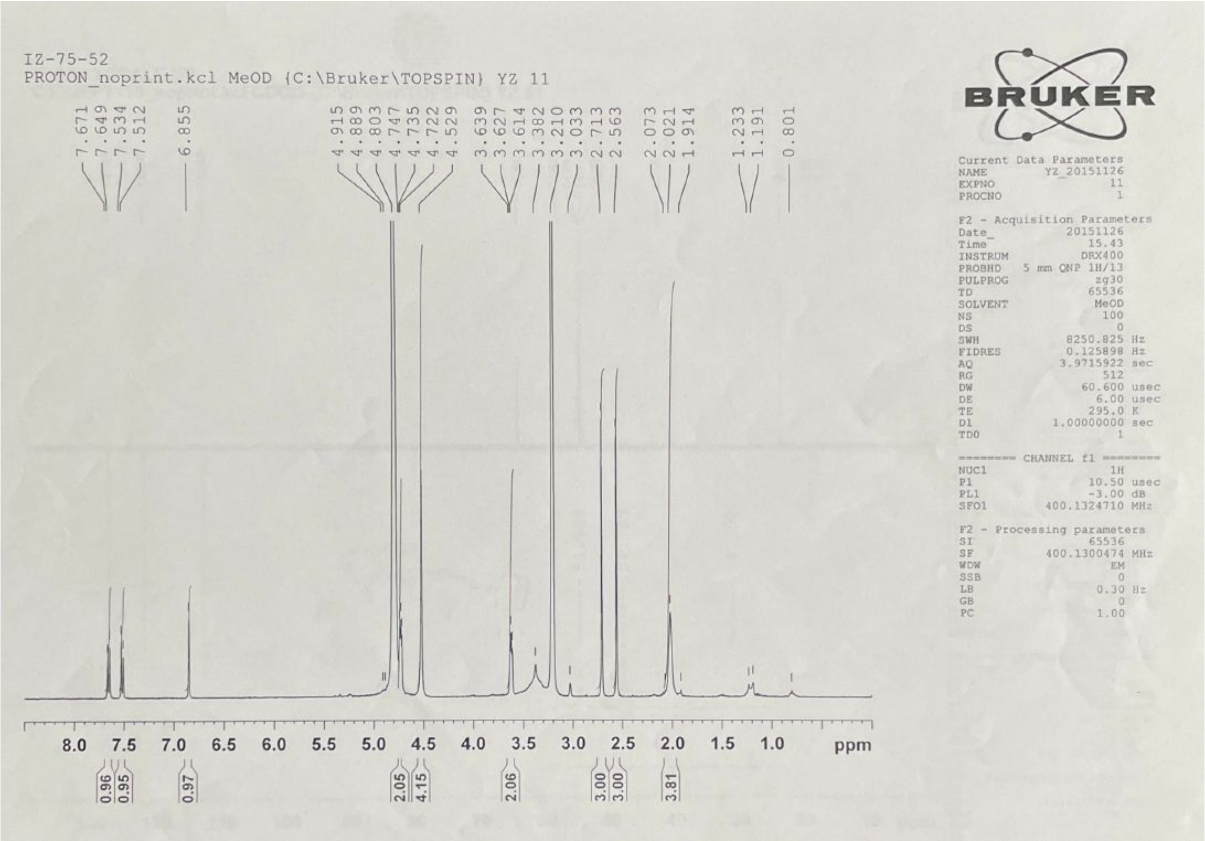

^{1}H-NMR of compound 1

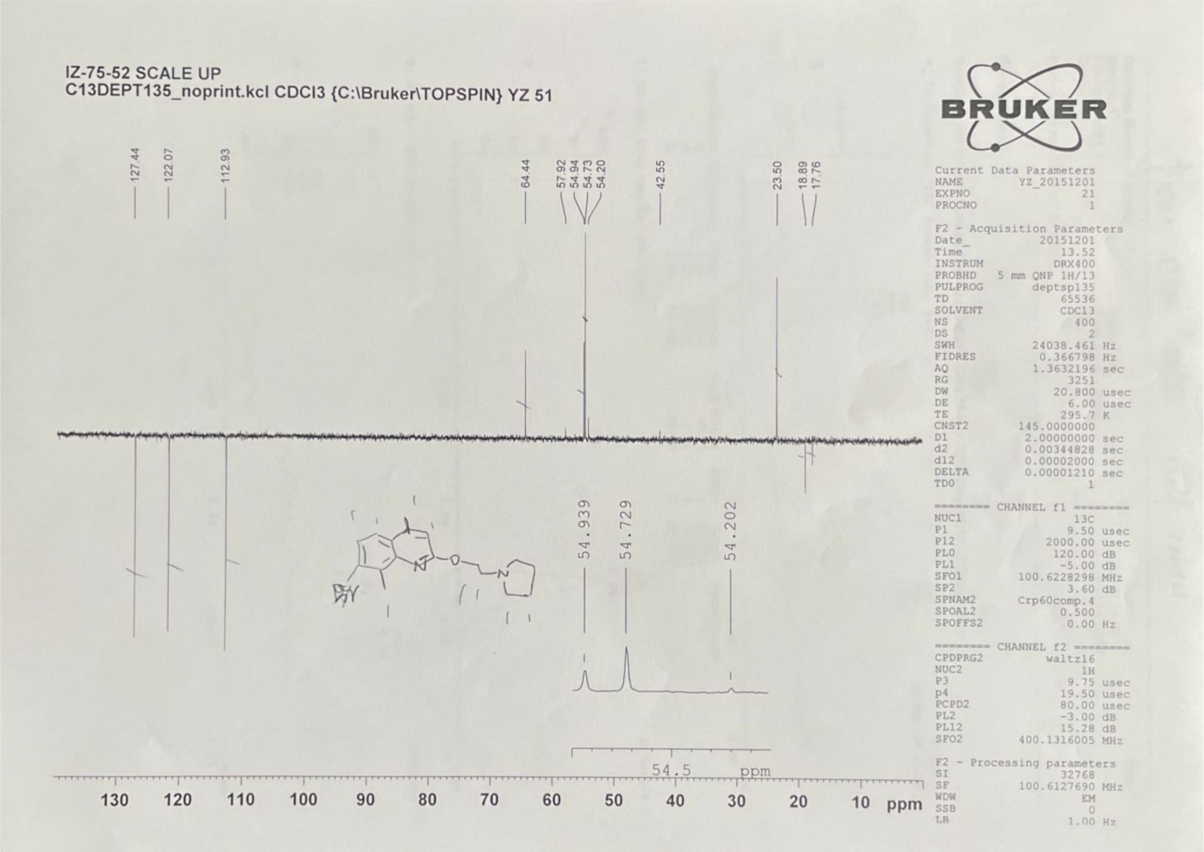

^{13}C-NMR of compound 1

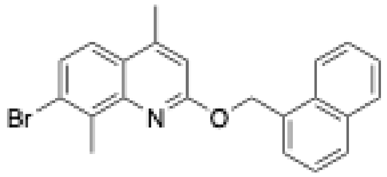

2

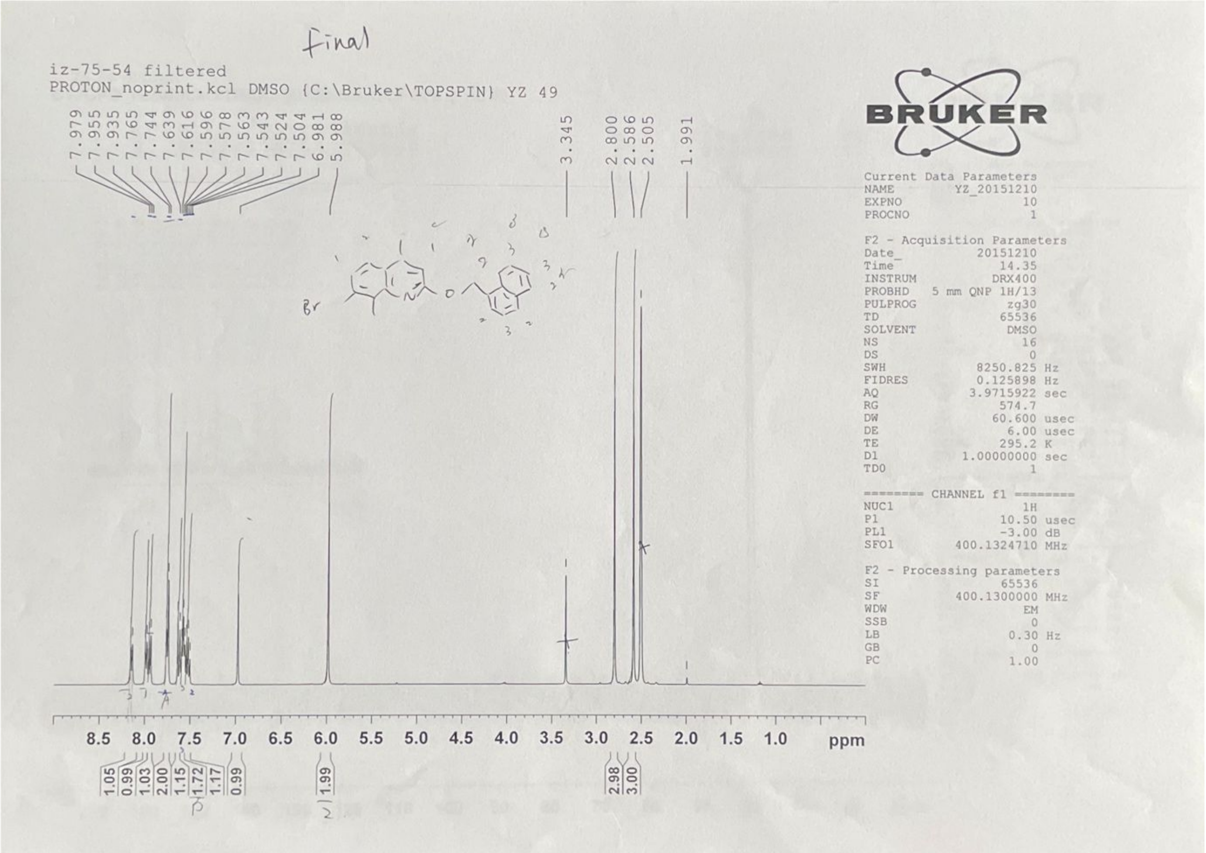

^{1}H-NMR of compound 2

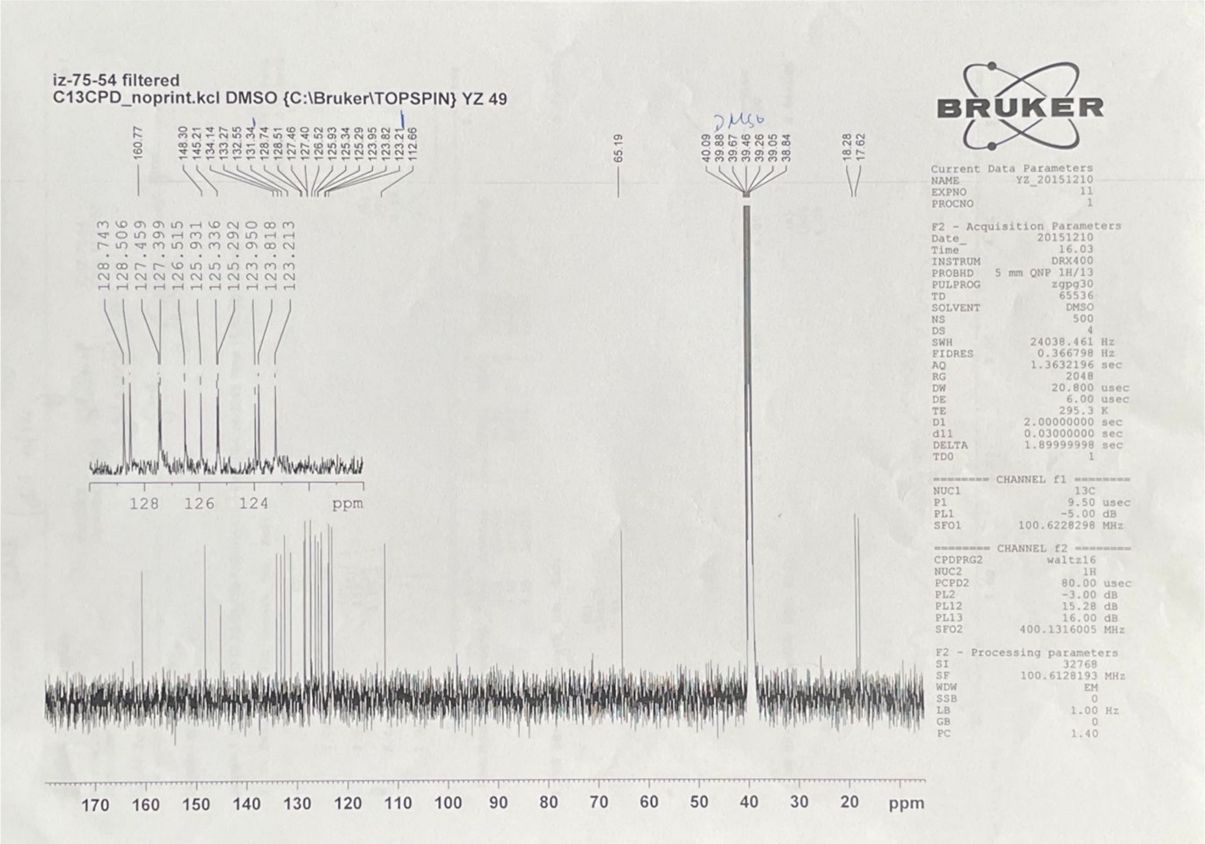

^{13}C-NMR of compound 2

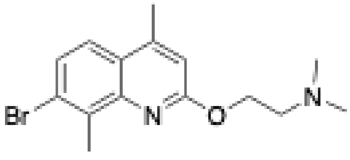

3

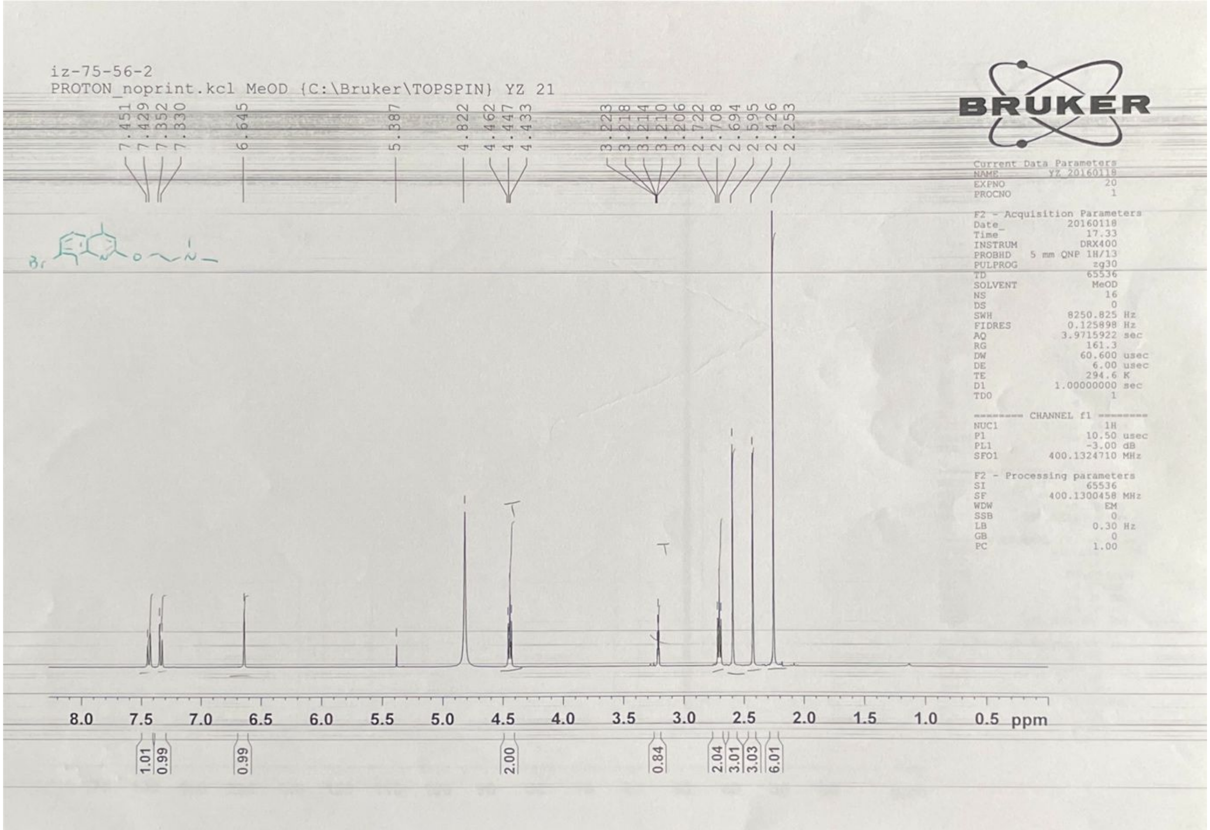

^{1}H-NMR of compound 3

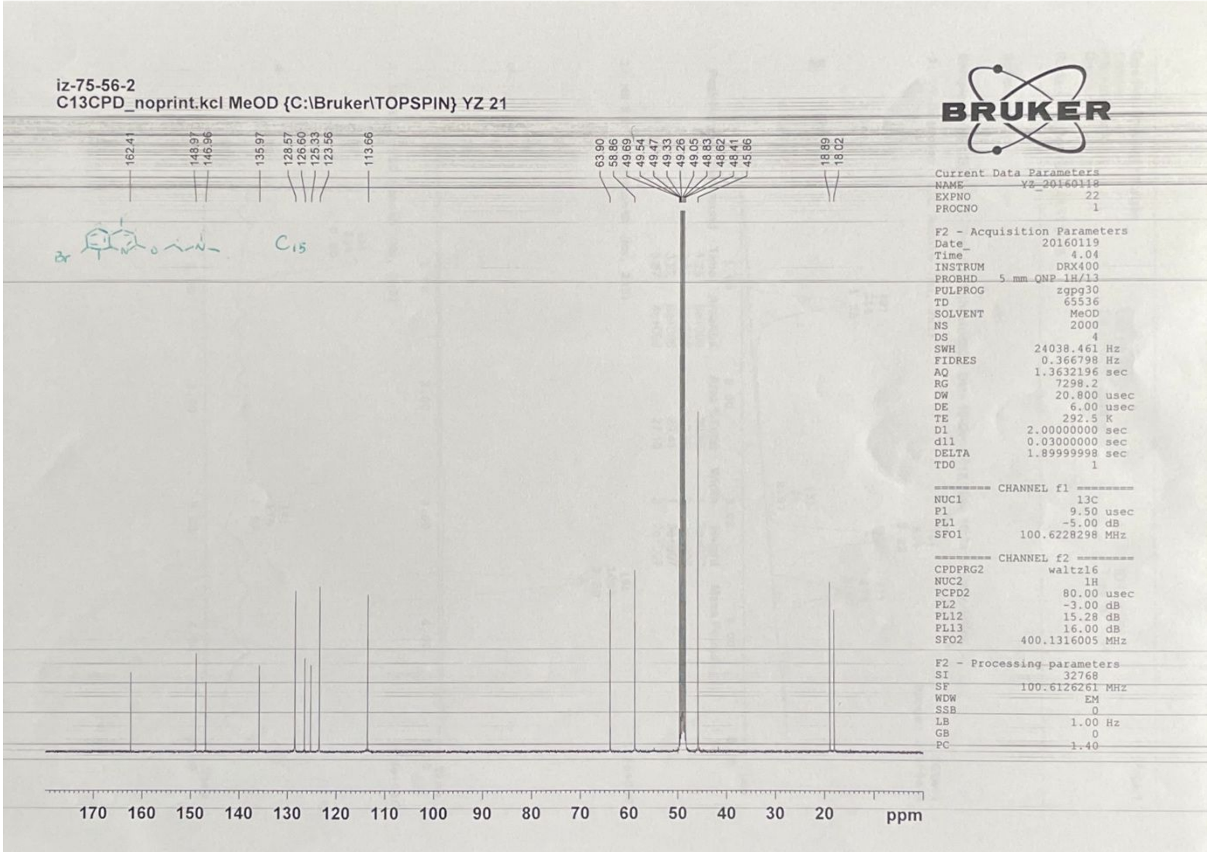

^{13}C-NMR of compound 3

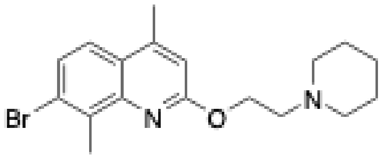

4

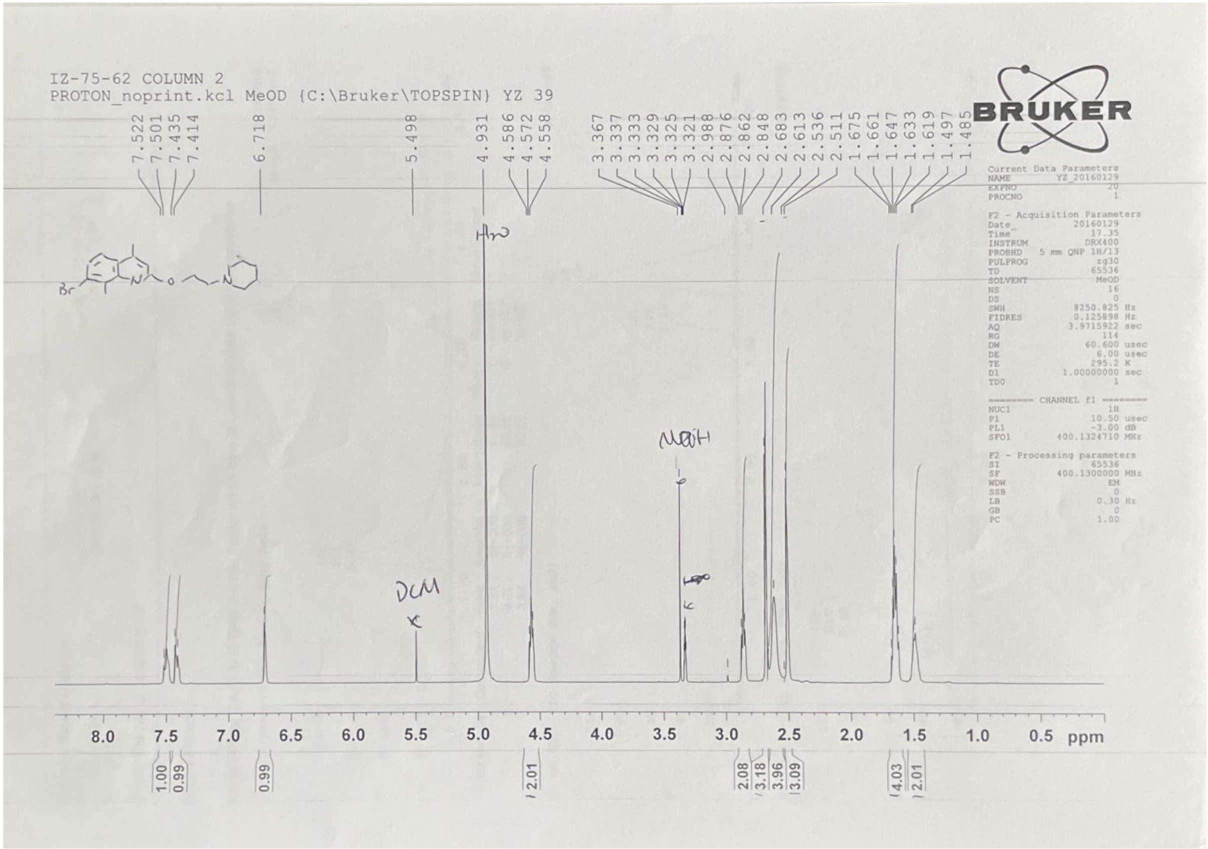

^{1}H-NMR of compound 4

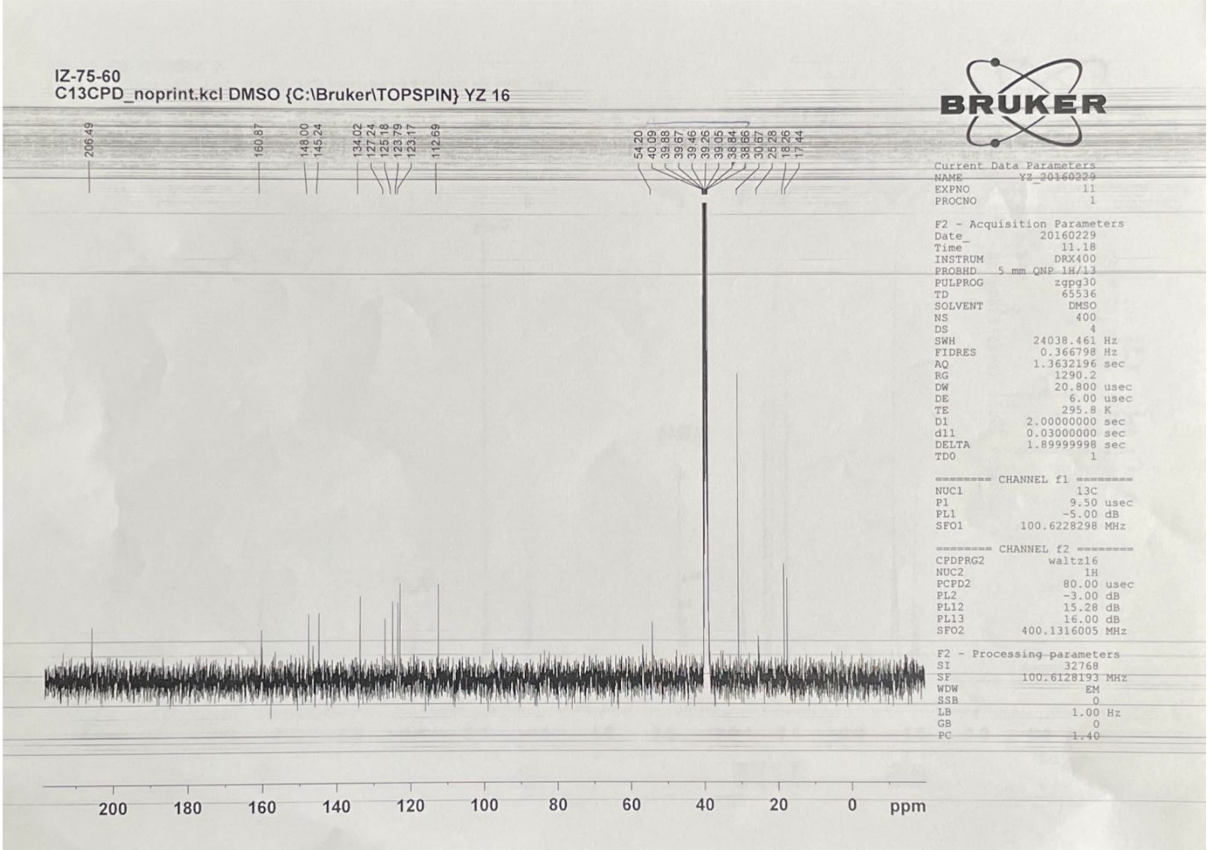

^{13}C-NMR of compound 4

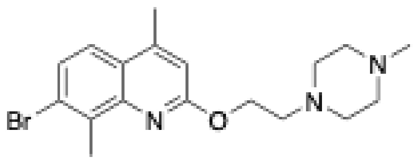

5

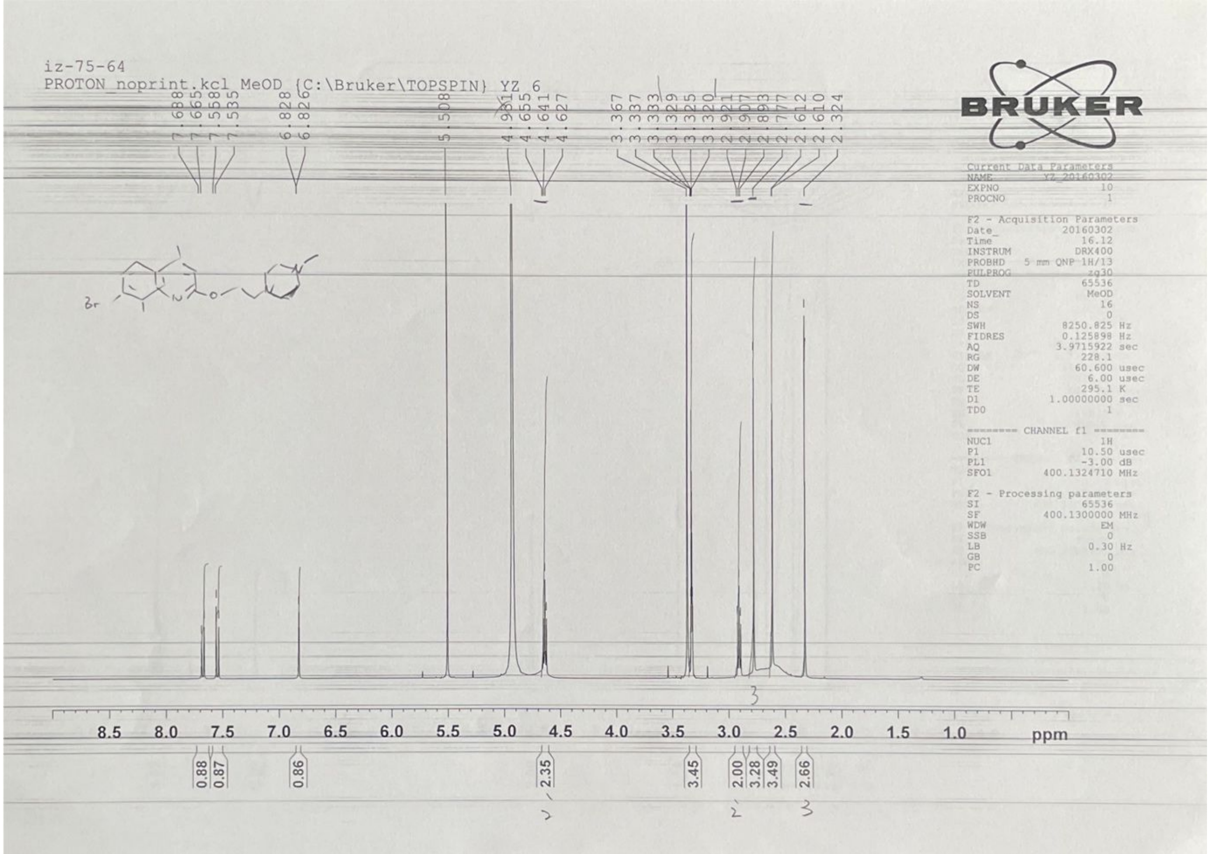

^{1}H-NMR of compound 5

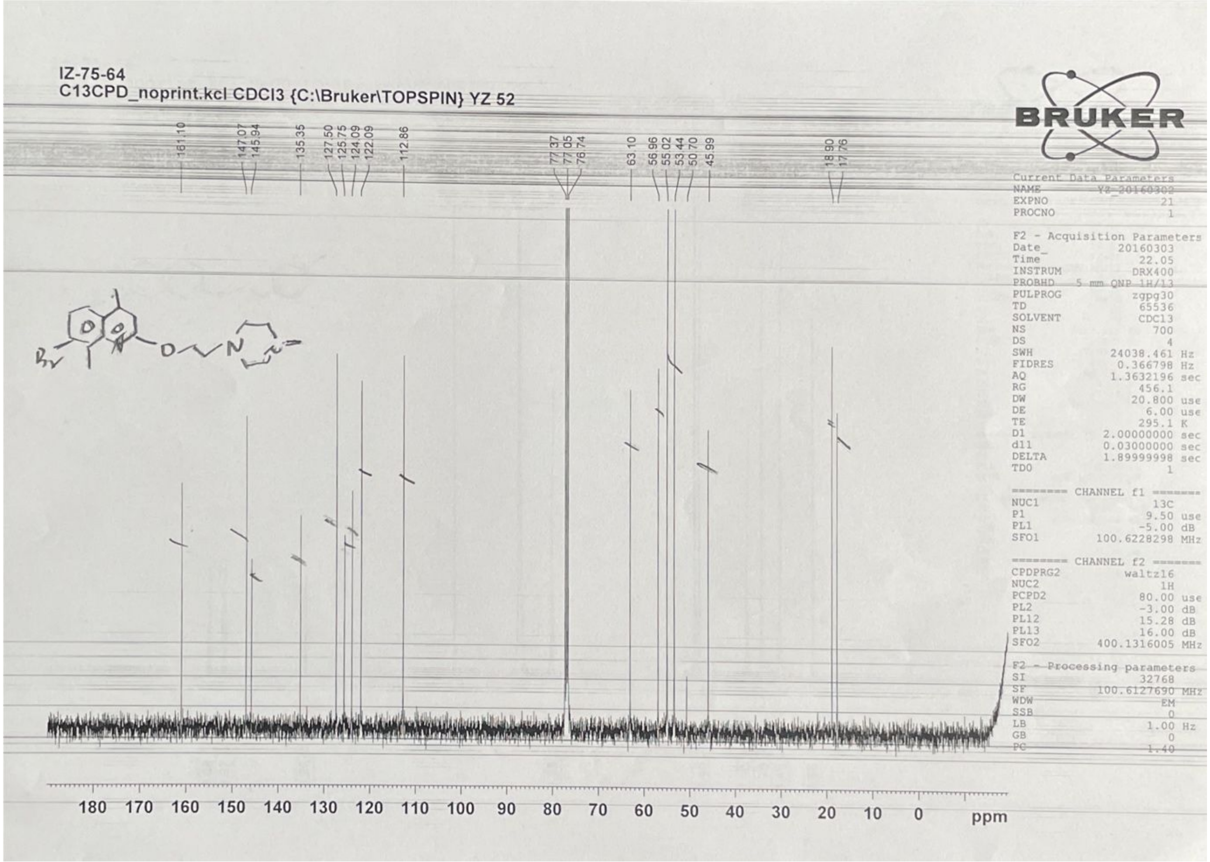

^{13}C-NMR of compound 5

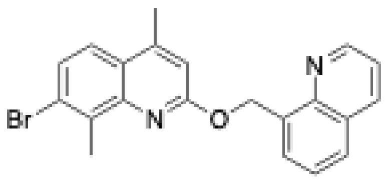

6

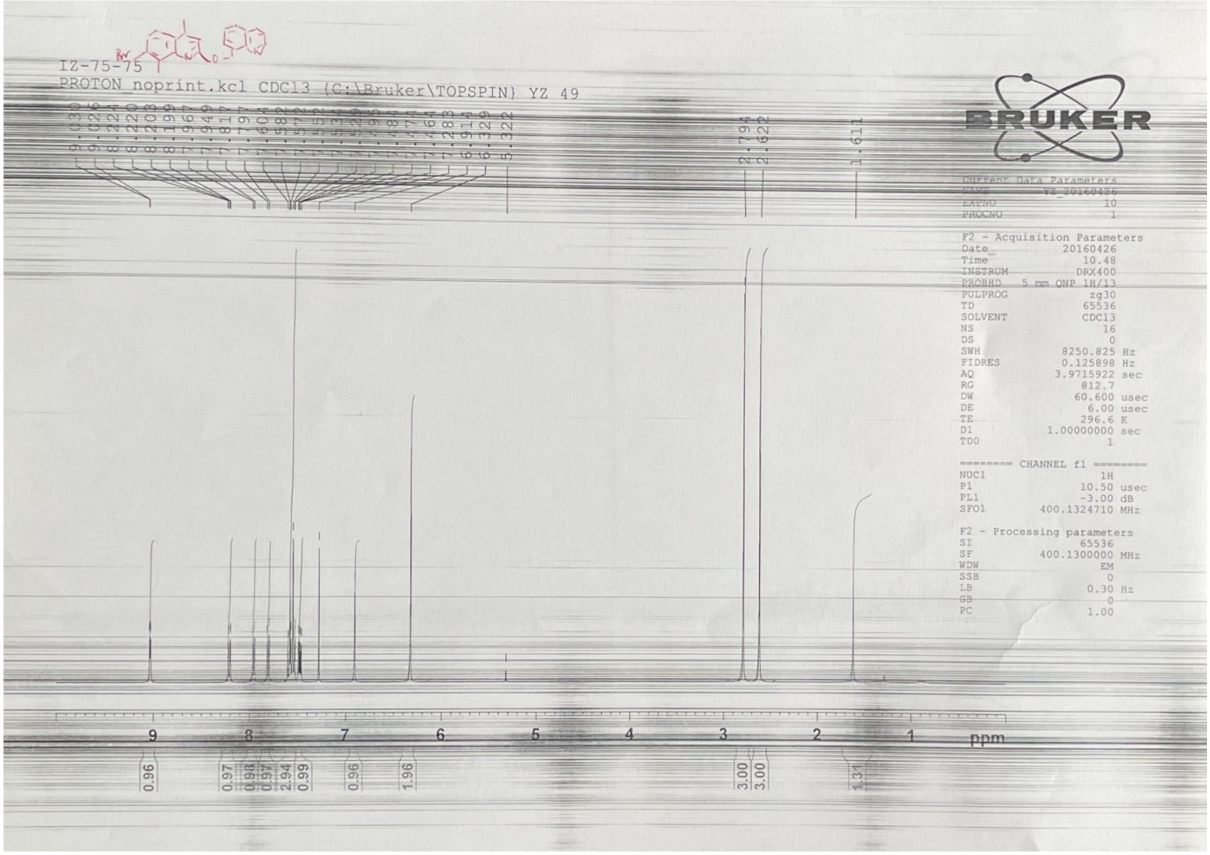

^{1}H-NMR of compound 6

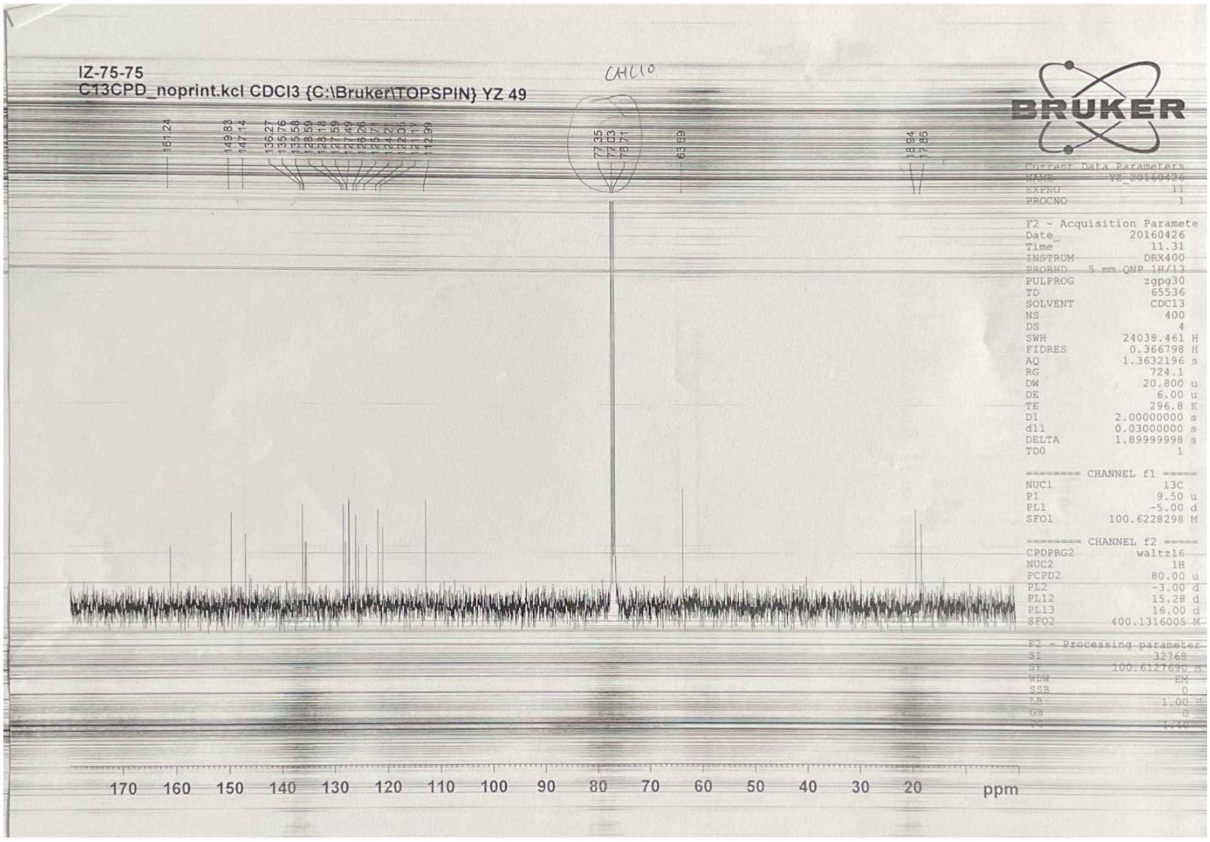

^{13}C-NMR of compound 6

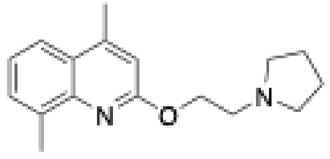

7

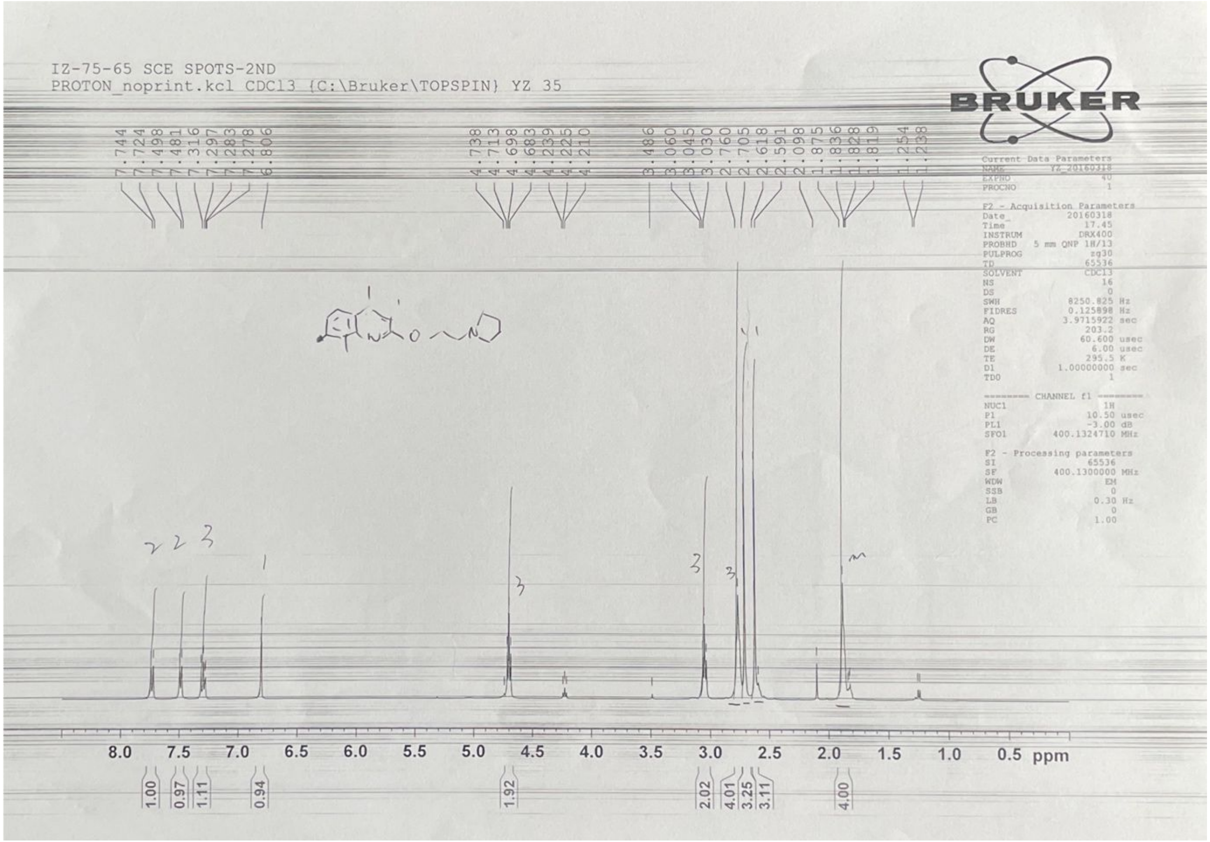

^{1}H-NMR of compound 7

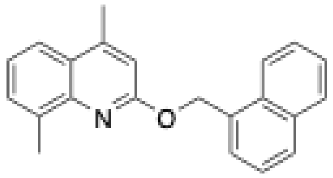

8

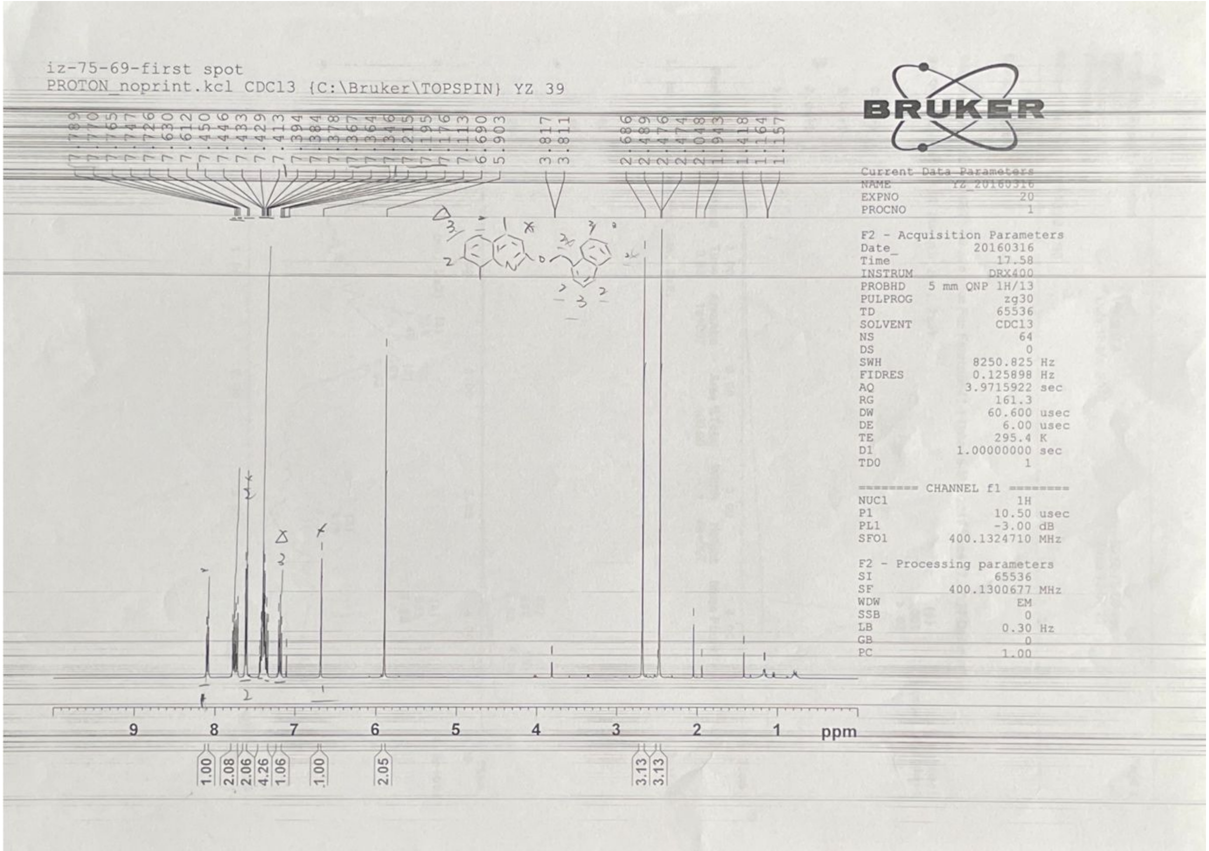

^{1}H-NMR of compound 8

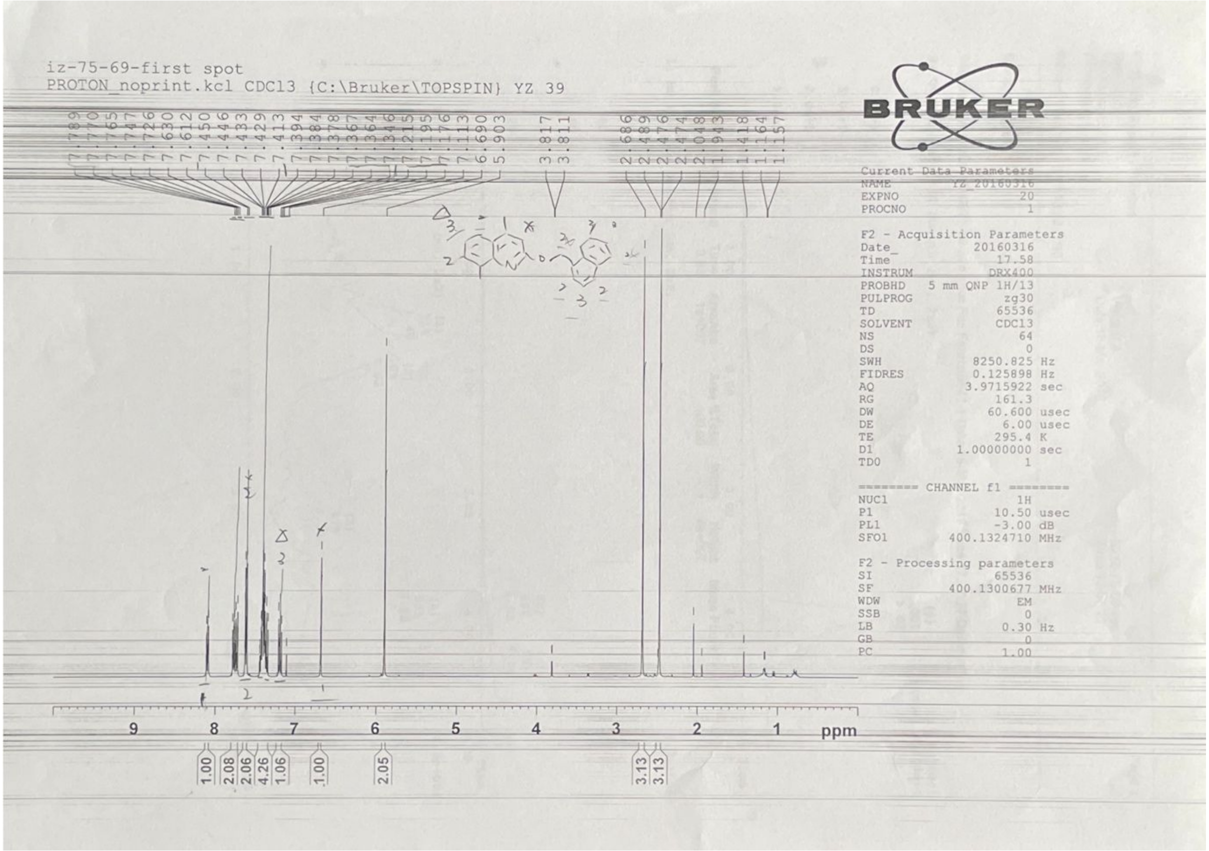

^{13}C-NMR of compound 8

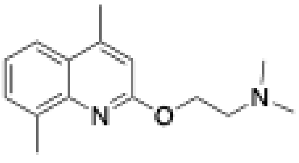

9

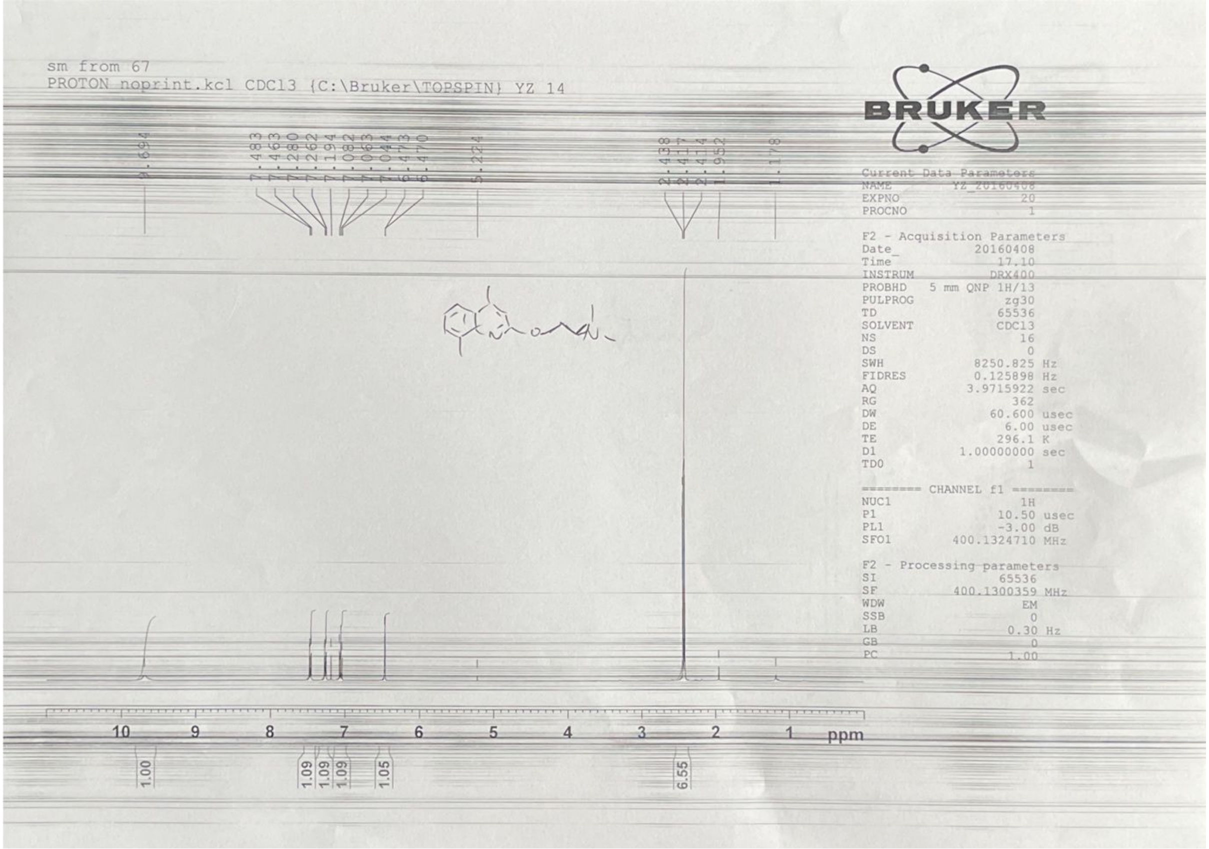

^{1}H-NMR of compound 9

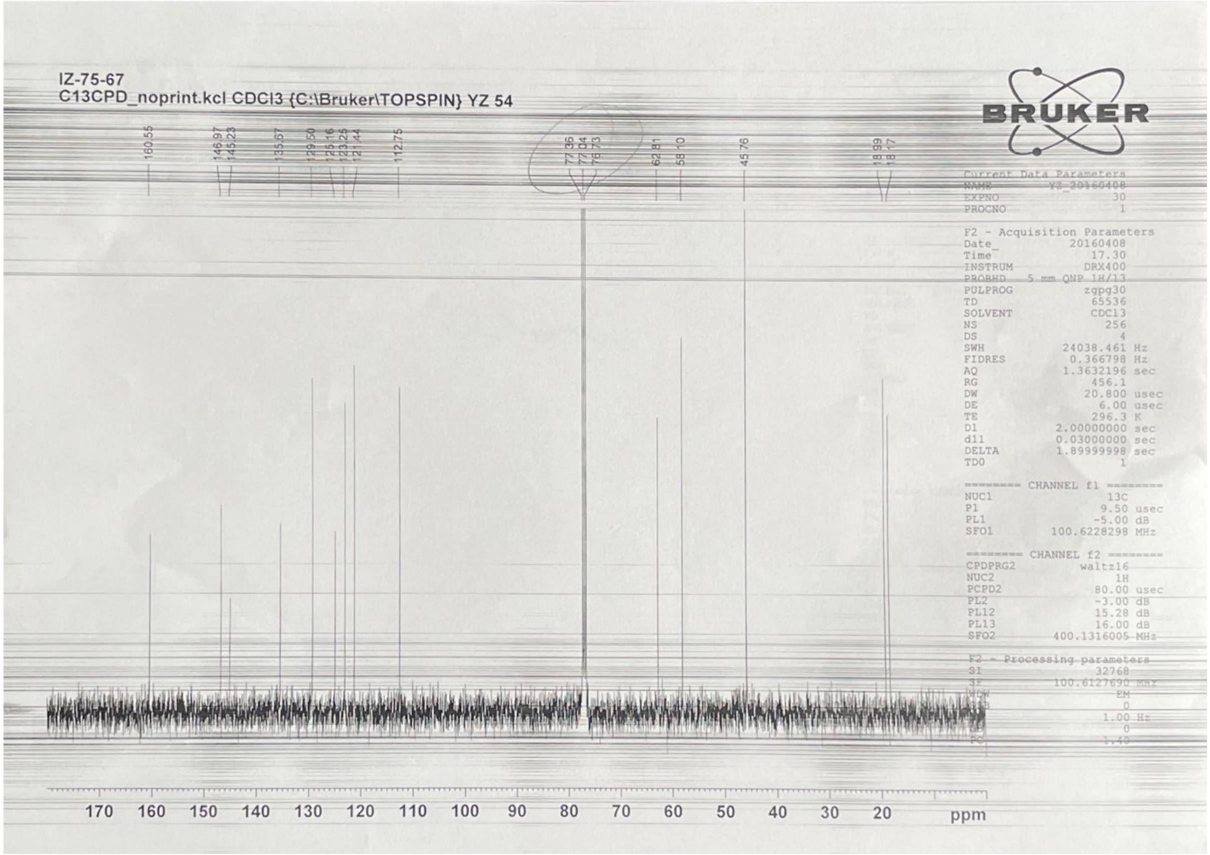

^{13}C-NMR of compound 9

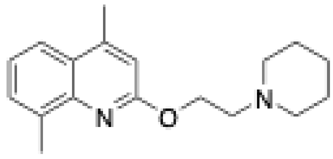

10

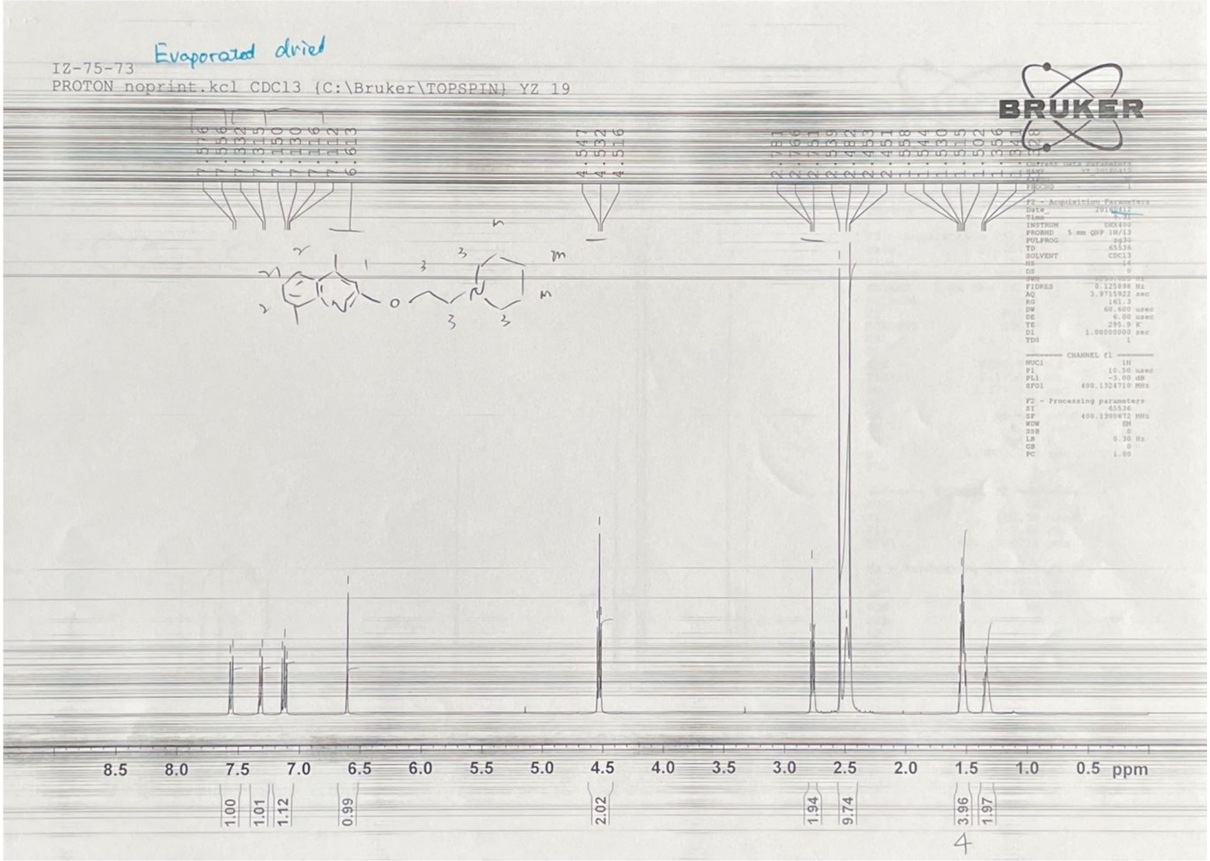

^{1}H-NMR of compound 10

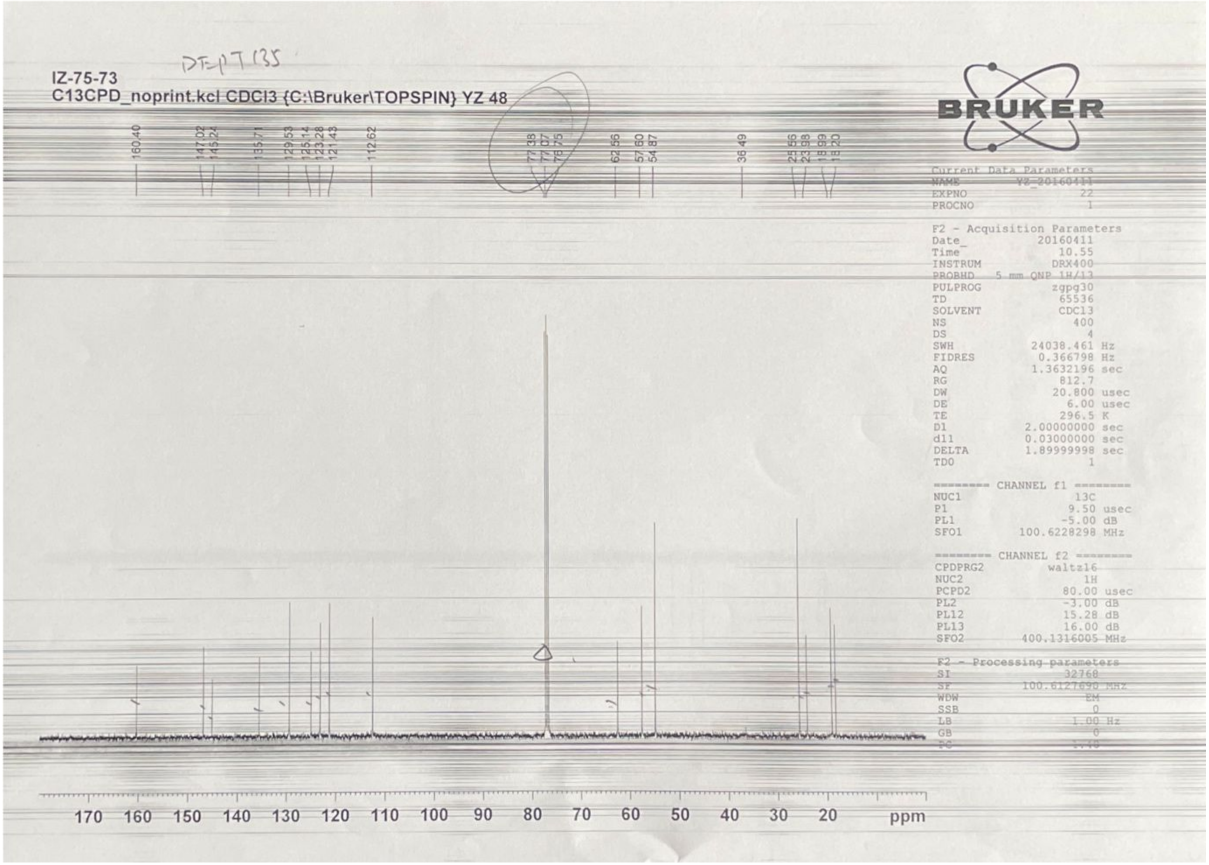

^{13}C-NMR of compound 10

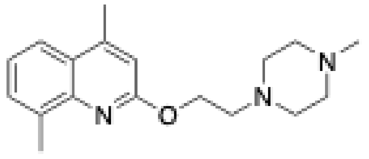

11

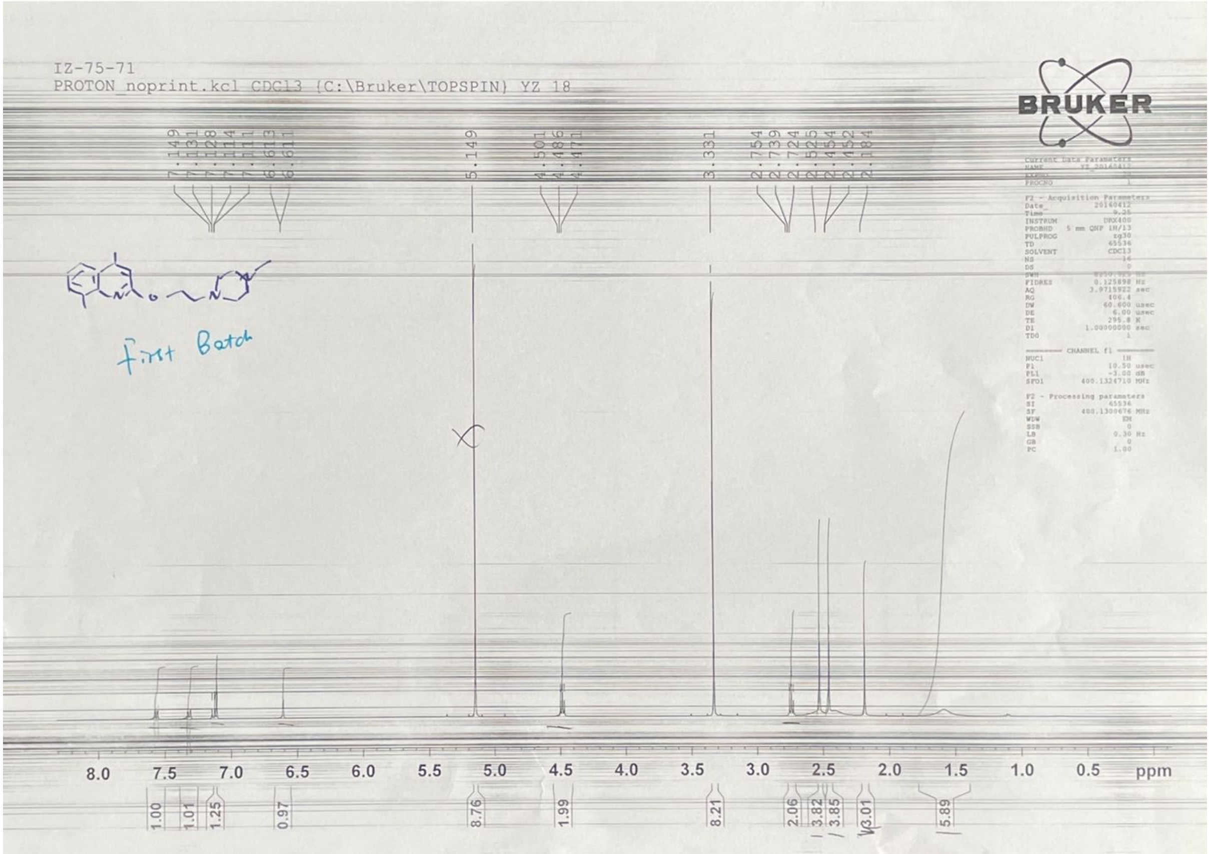

^{1}H-NMR of compound 11

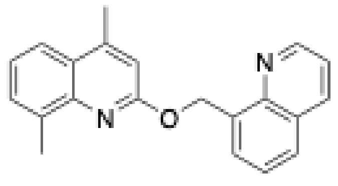

12

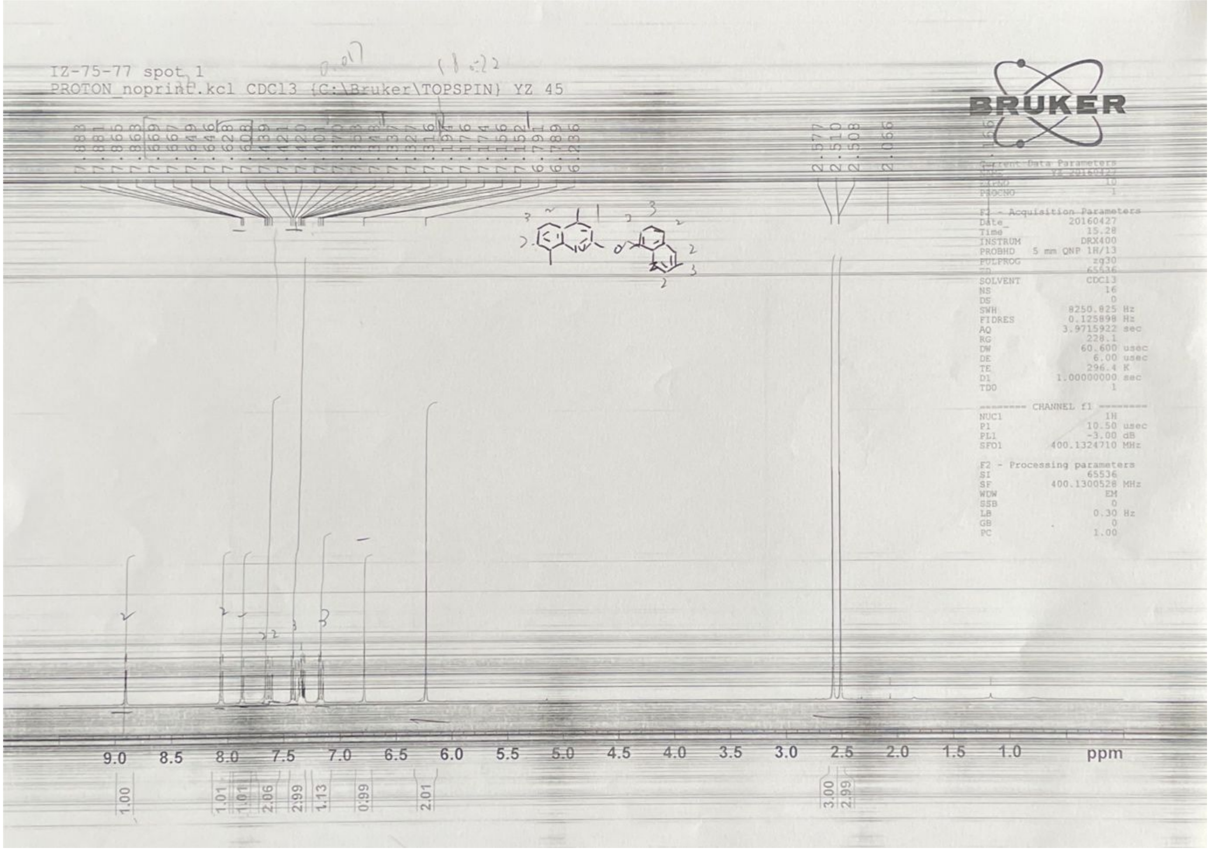

^{1}H-NMR of compound 12

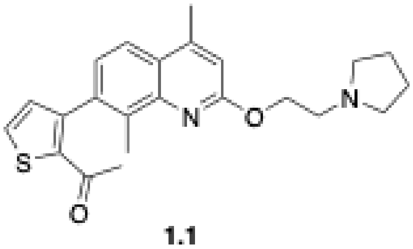

1.1

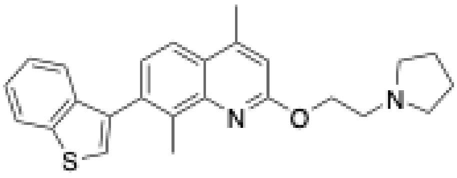

1.2

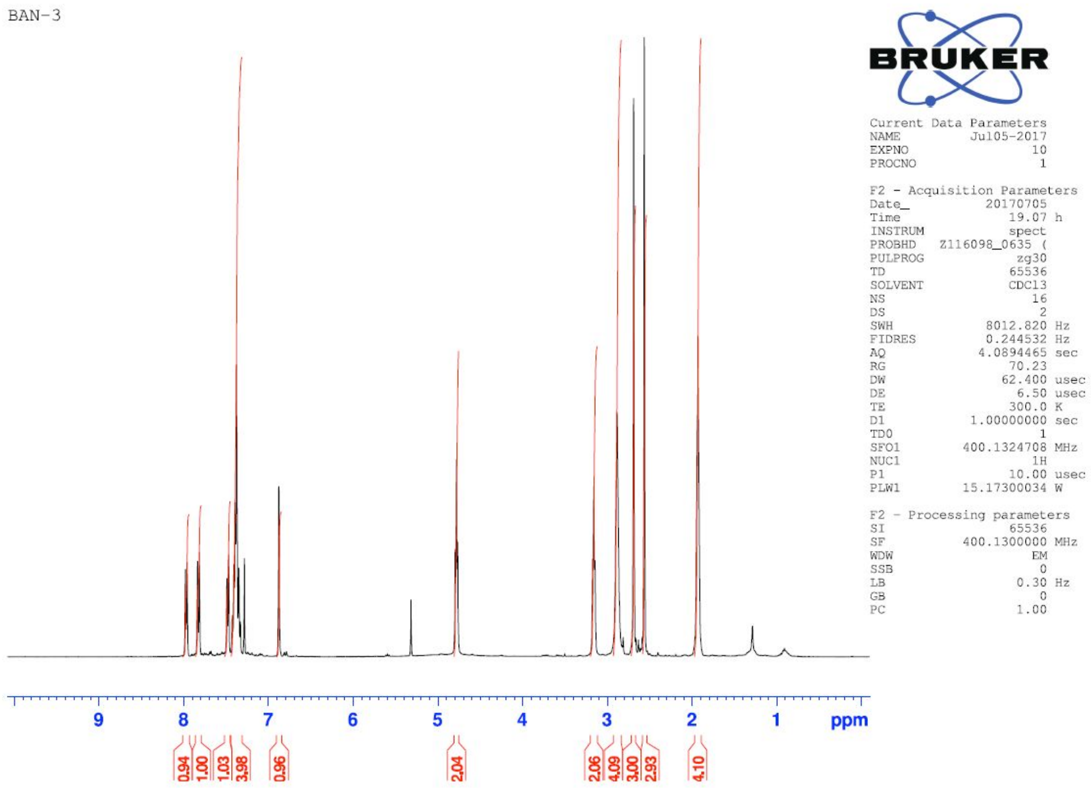

Figure S24. ^{1}H-NMR of compound 1.2

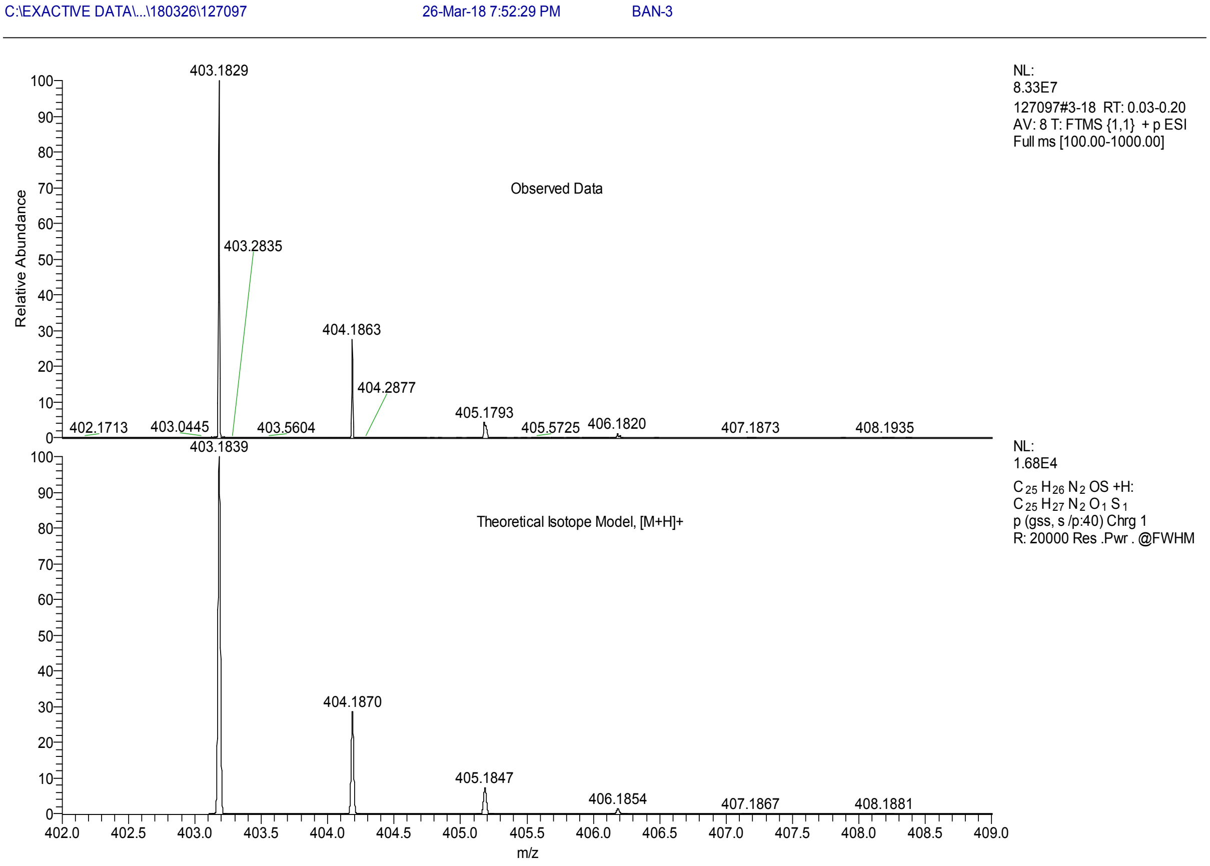

HR-MS of compound 1.2

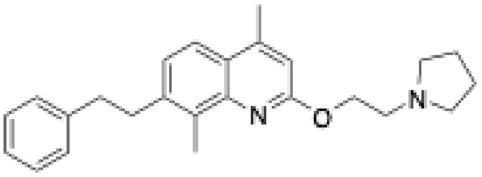

1.3

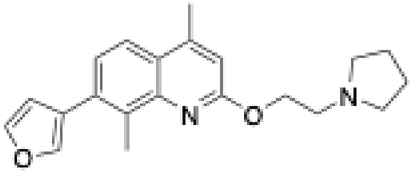

1.4

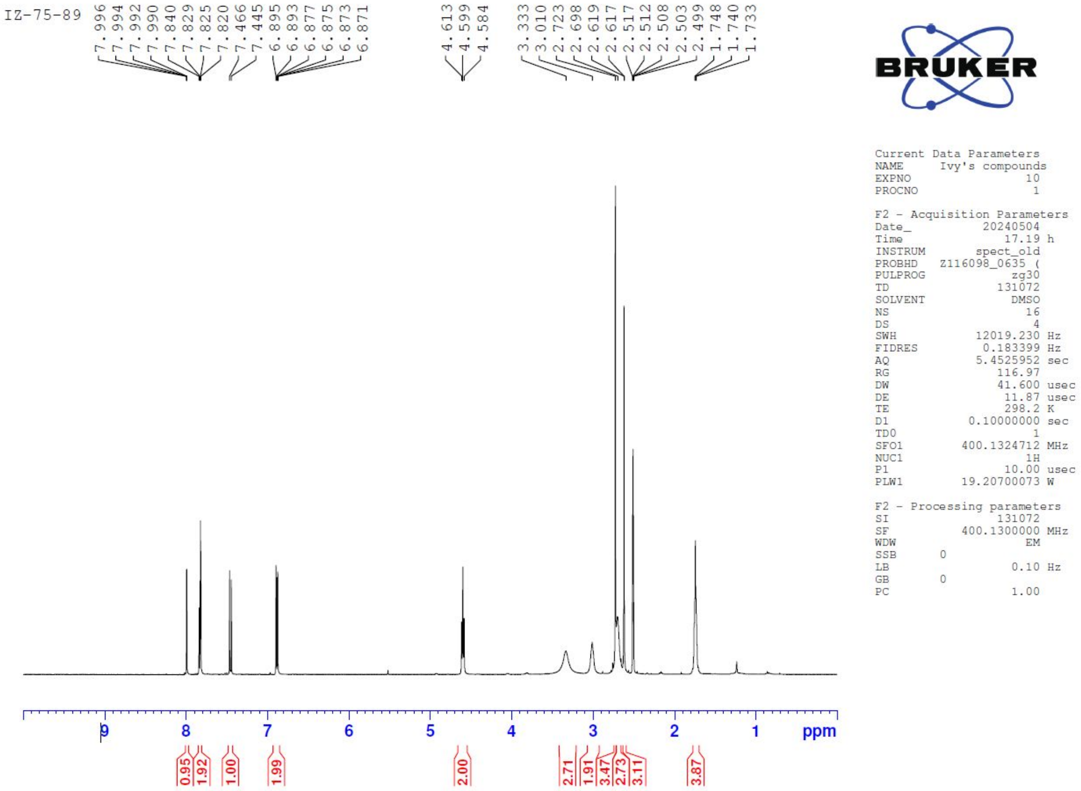

^{1}H-NMR of compound 1.4

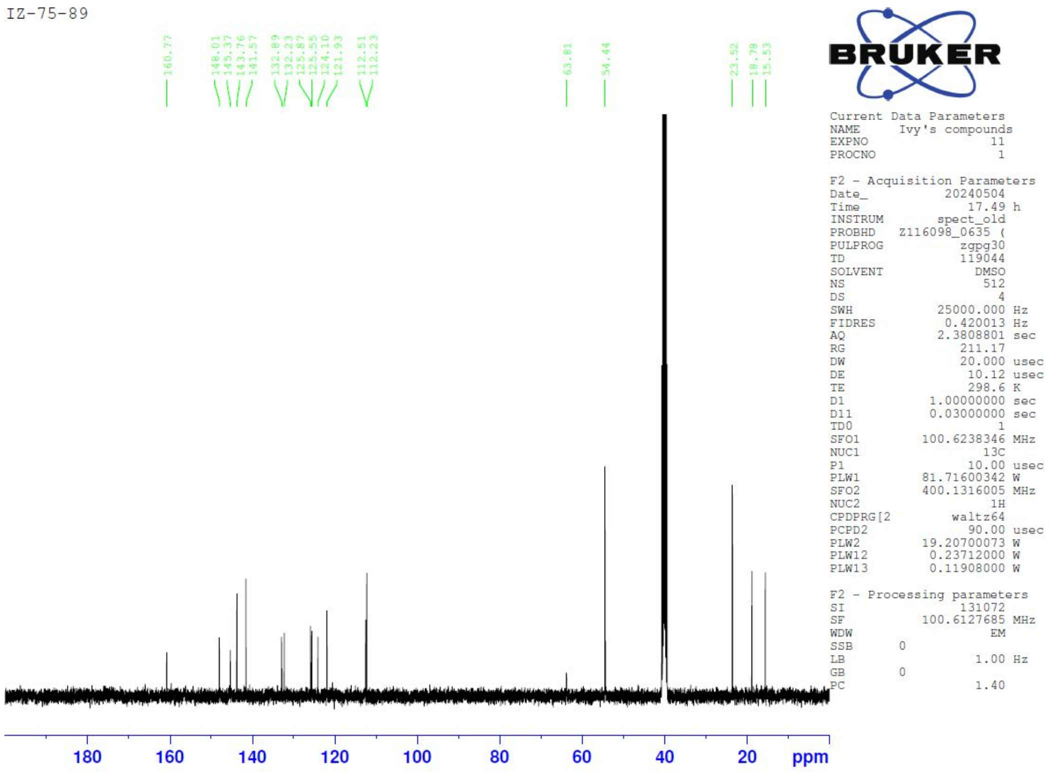

^{13}C-NMR of compound 1.4

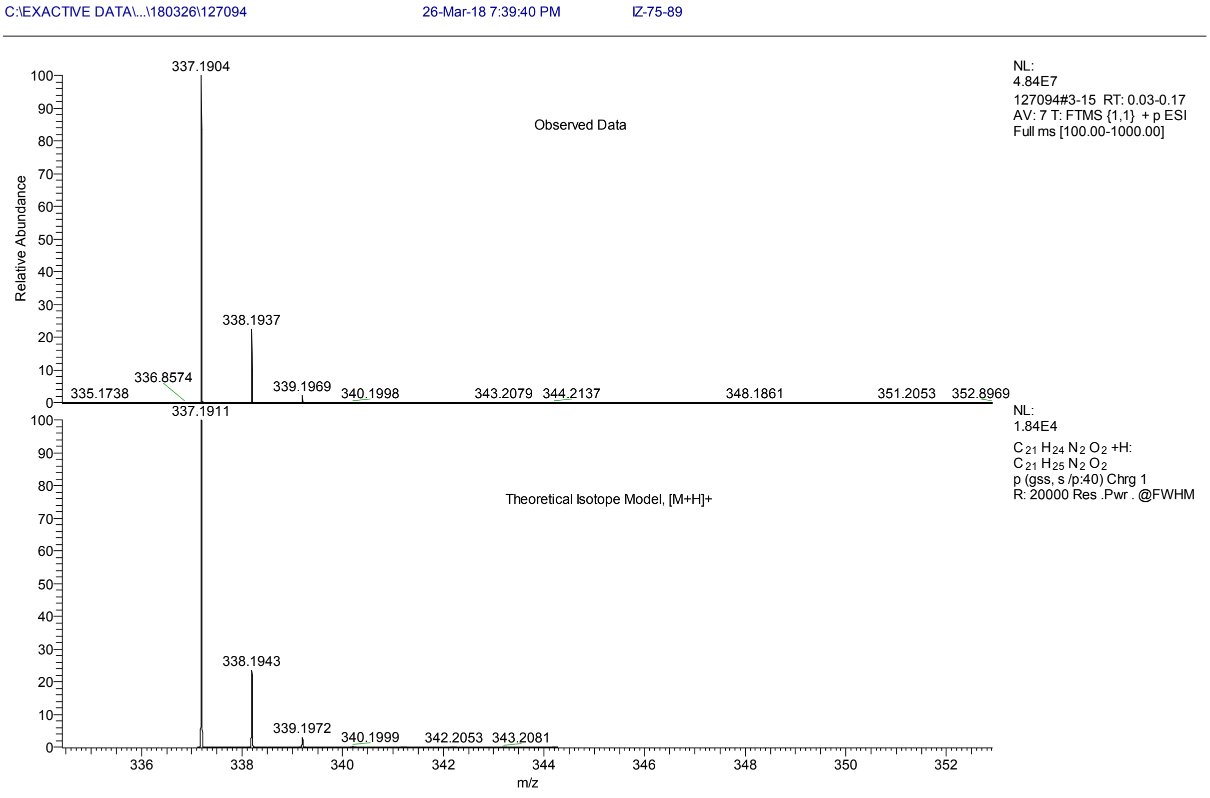

HR-MS of compound 1.4

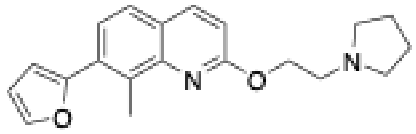

1.5

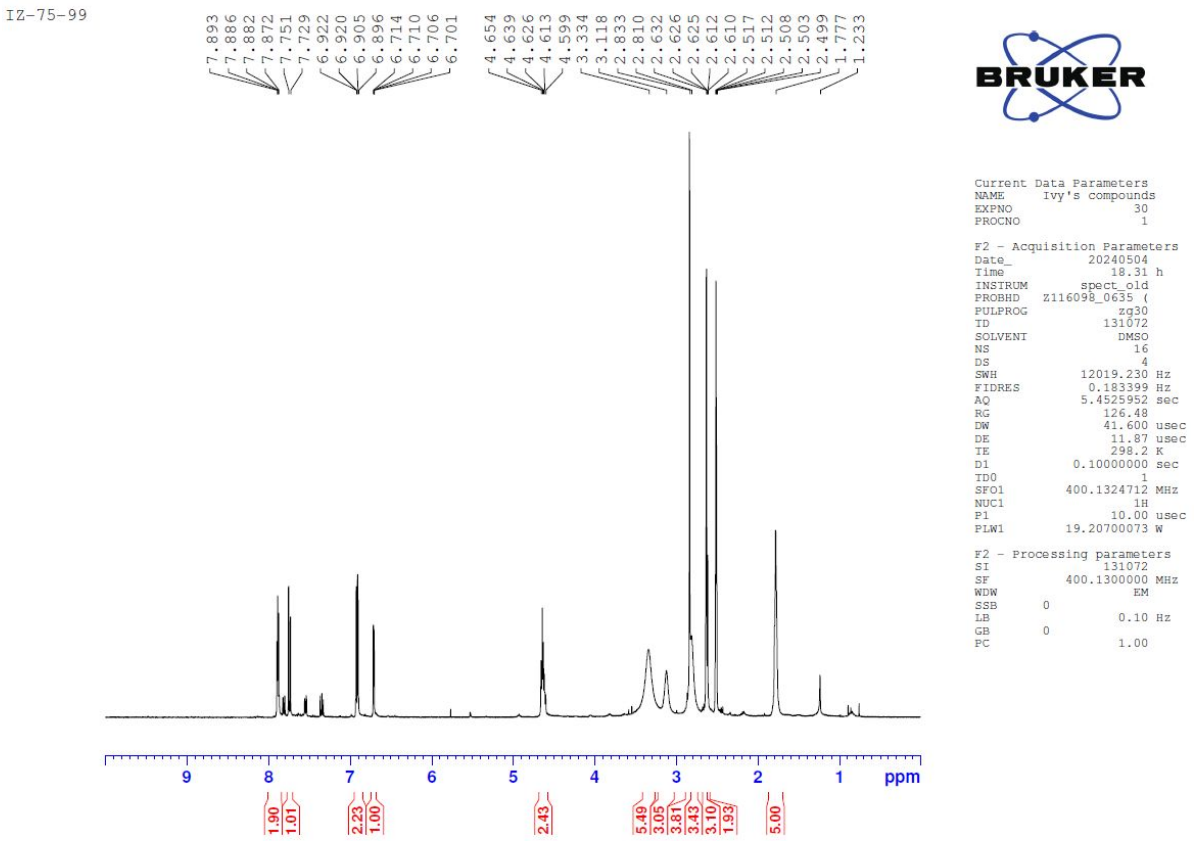

^{1}H-NMR of compound 1.5

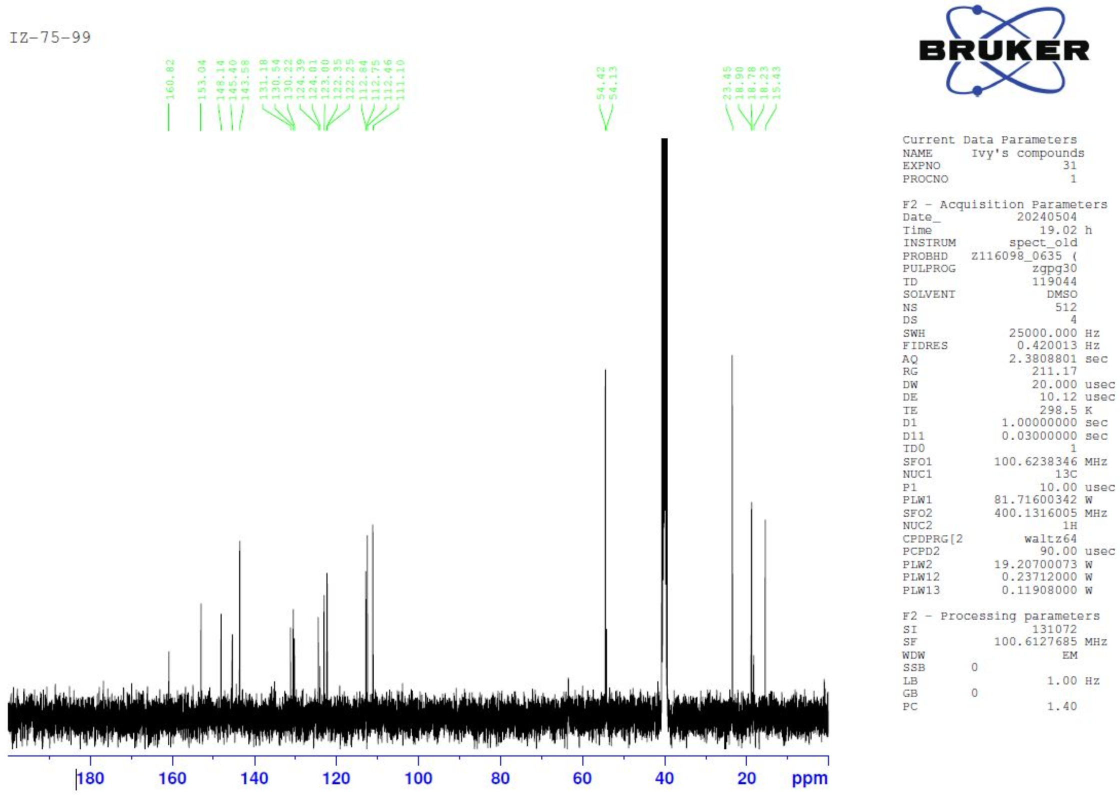

^{13}C-NMR of compound 1.5

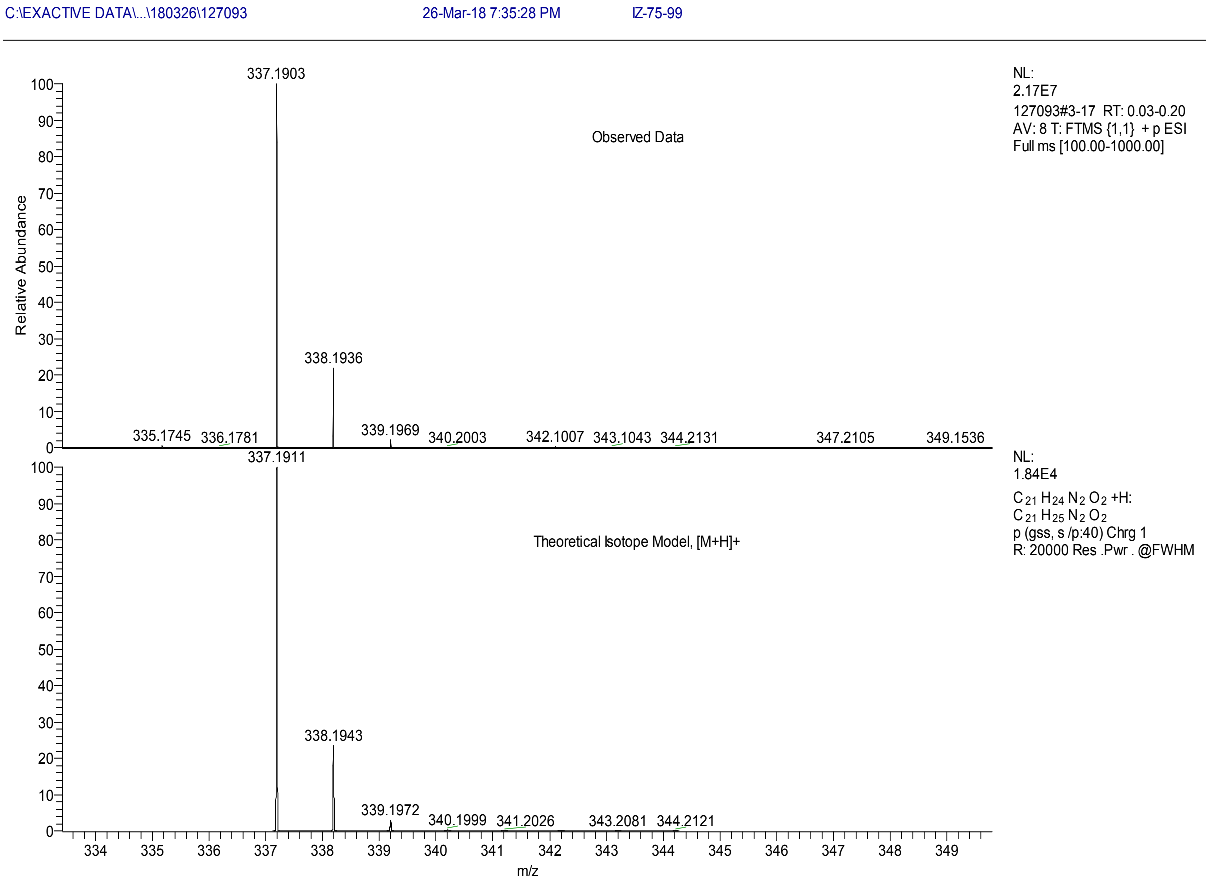

HR-MS of compound 1.5

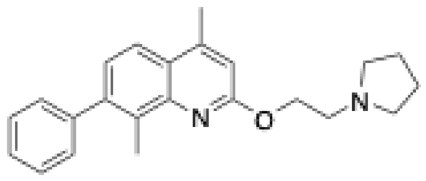

1.6

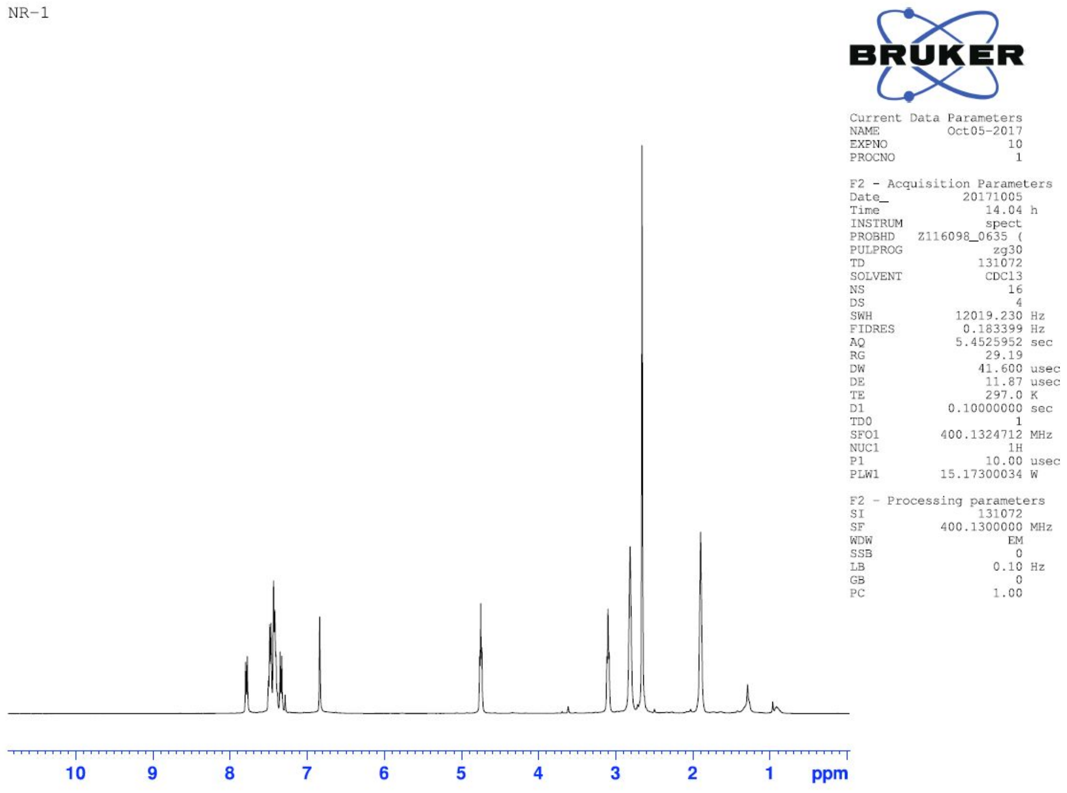

^{1}H-NMR of compound 1.6

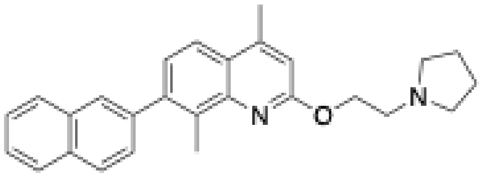

1.7

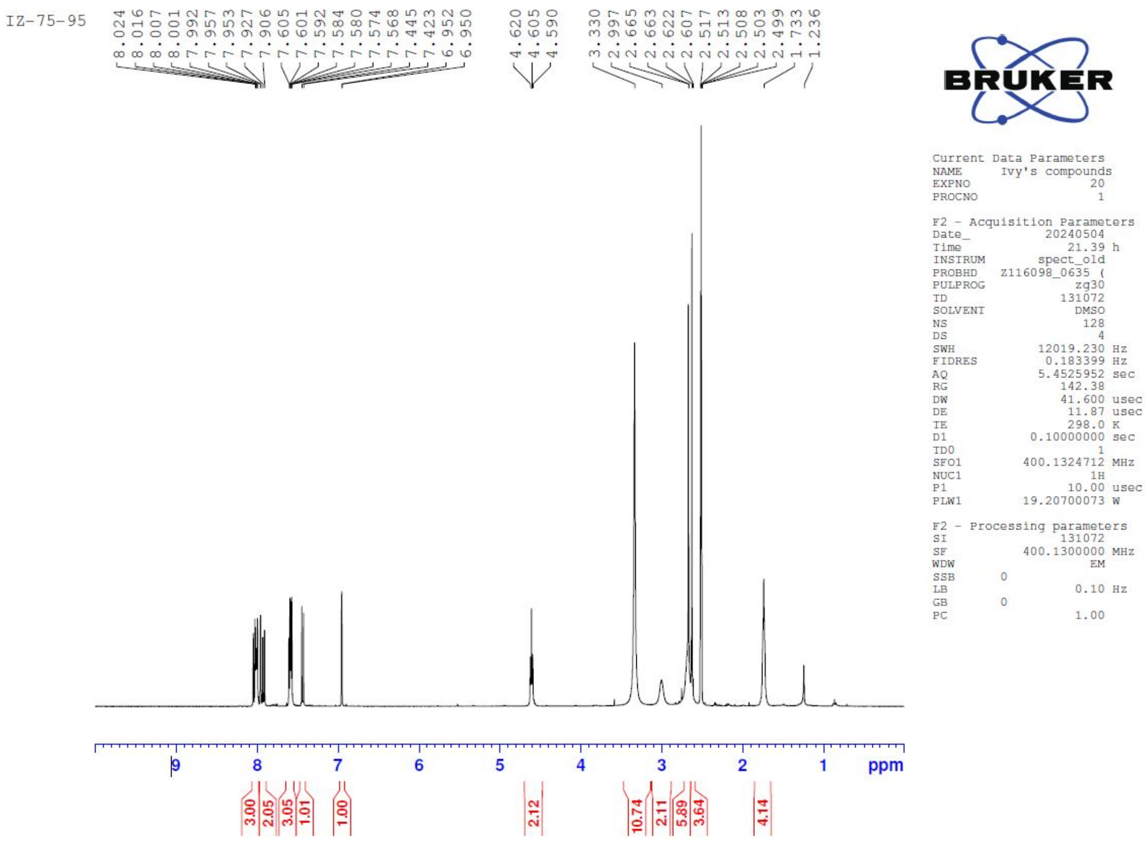

^{1}H-NMR of compound 1.7

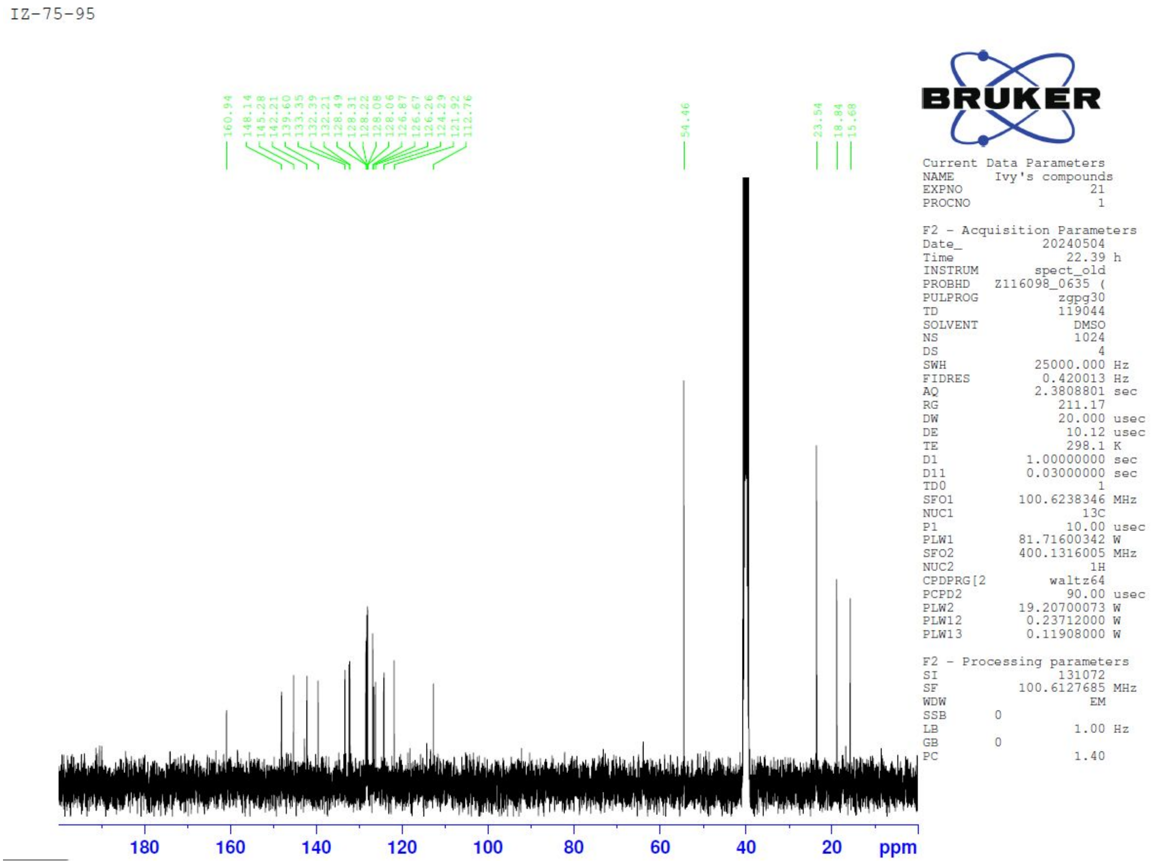

^{13}C-NMR of compound 1.7

C:\EXACTIVE DATA\...\180326\127095 26-Mar-18 7:43:52 PM IZ-75-95

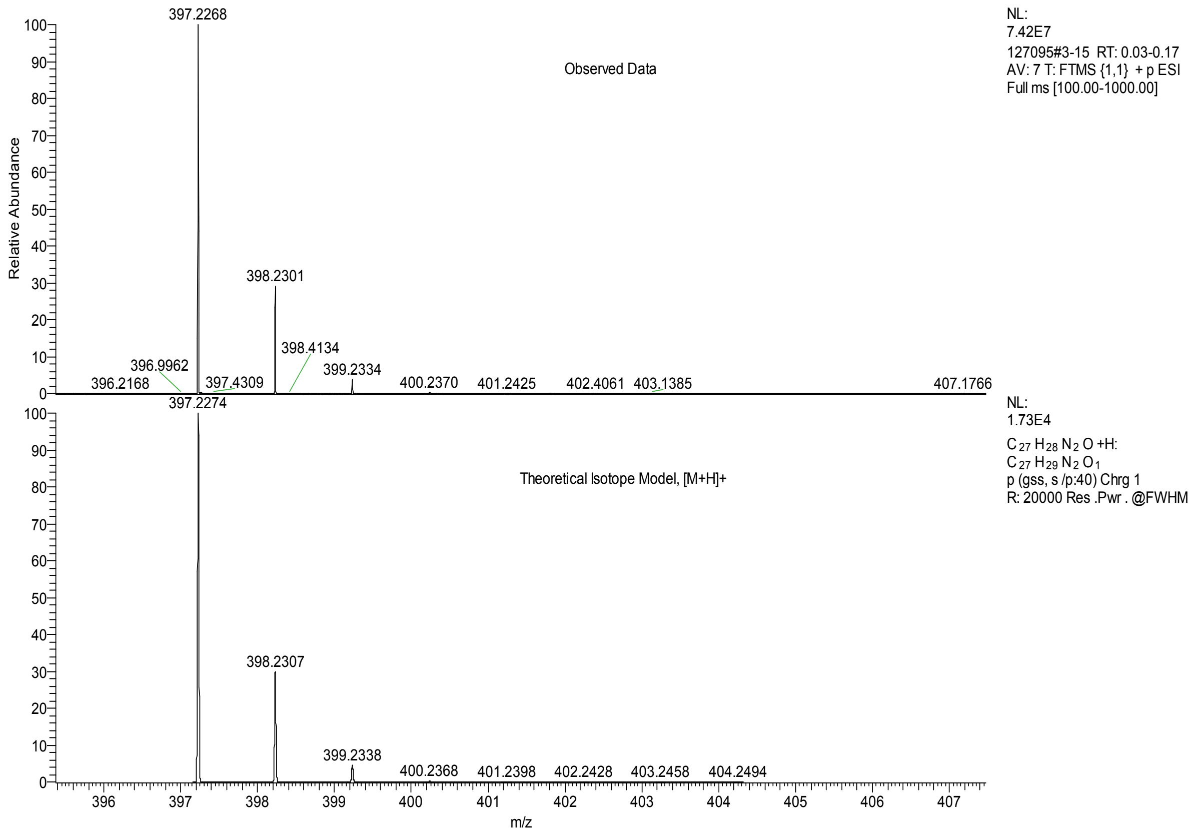

HR-MS of compound 1.7

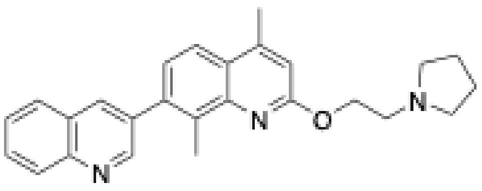

1.8

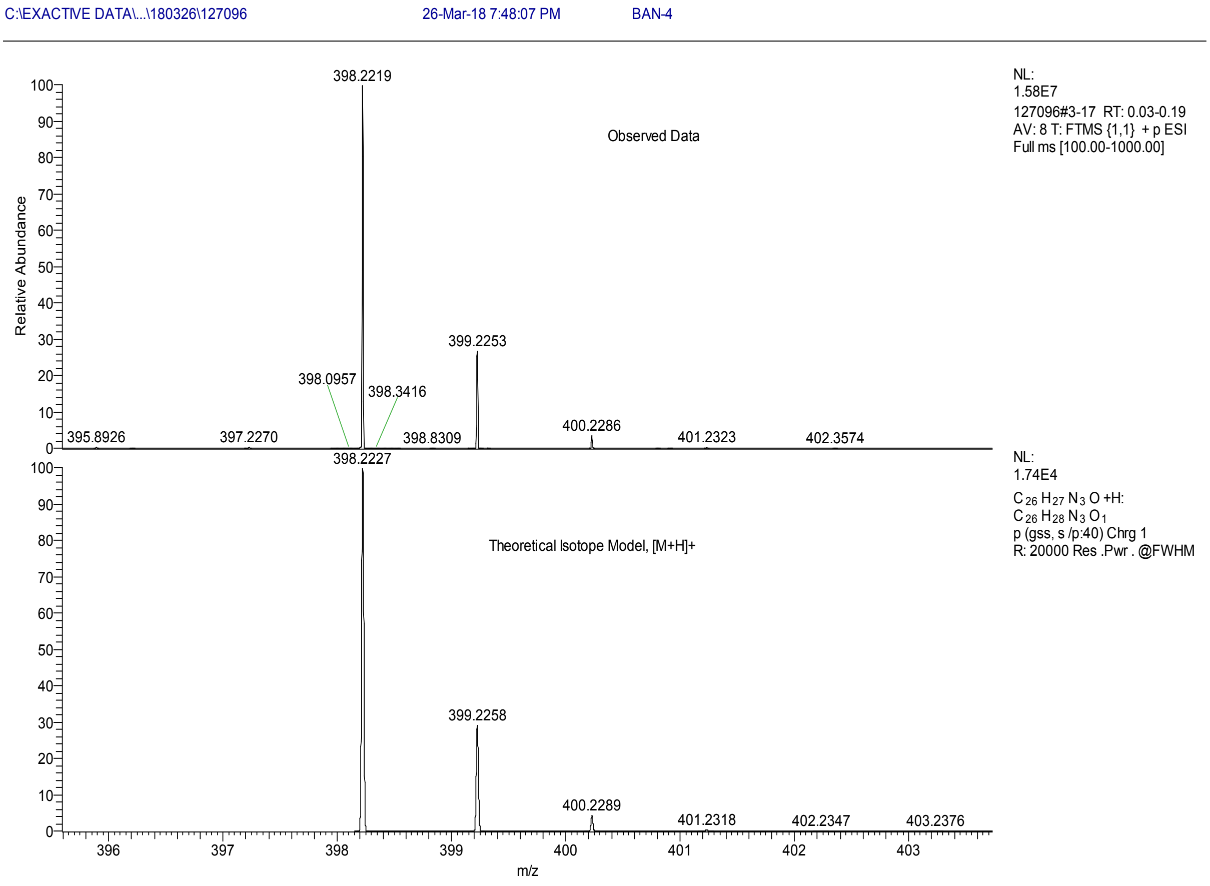

HR-MS of compound 1.8

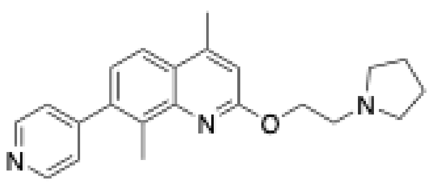

1.9

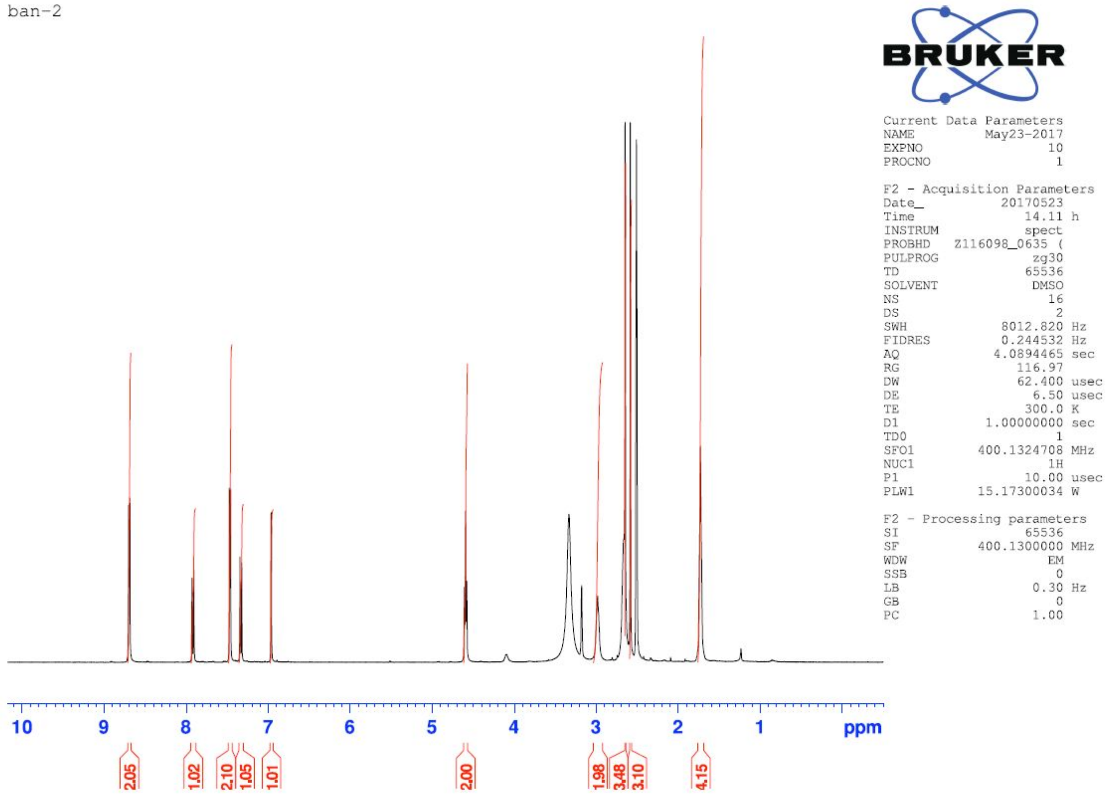

^{1}H-NMR of compound 1.9

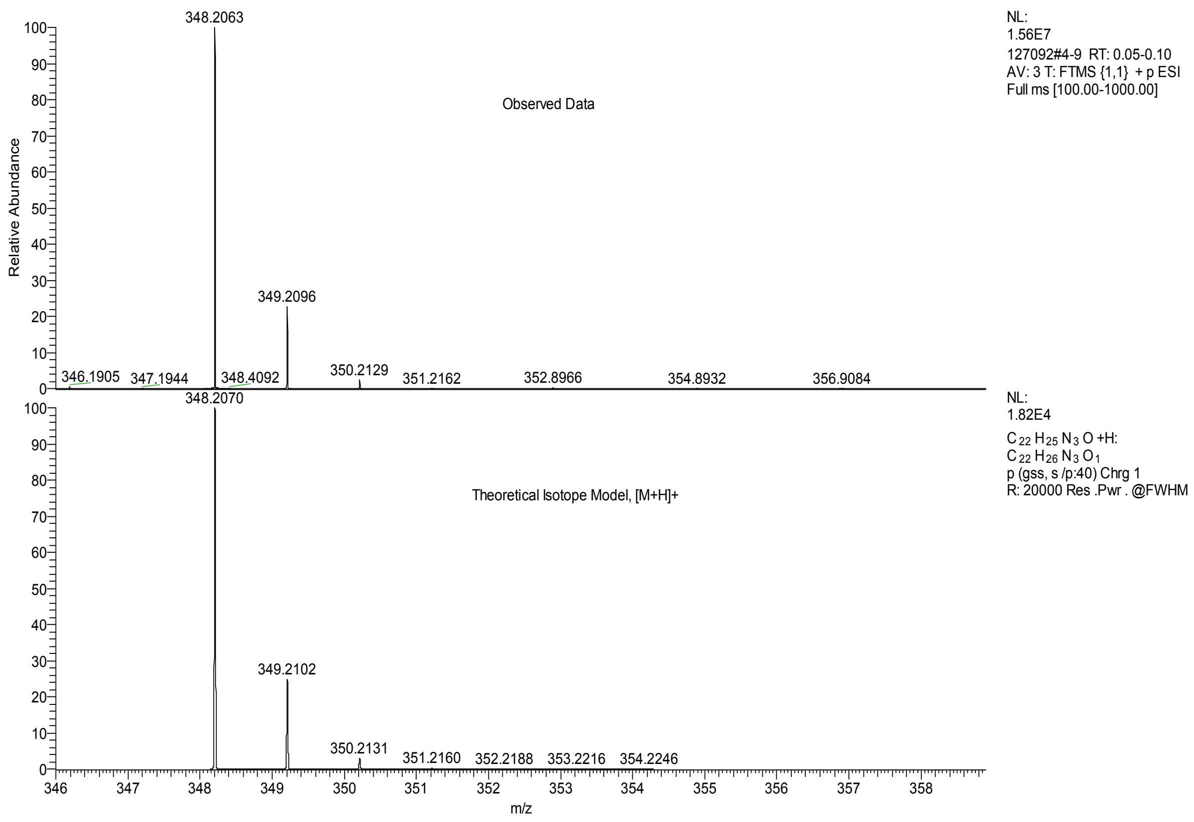

HR-MS of compound 1.9

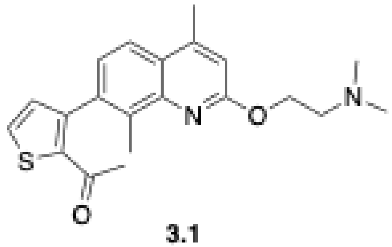

3.1

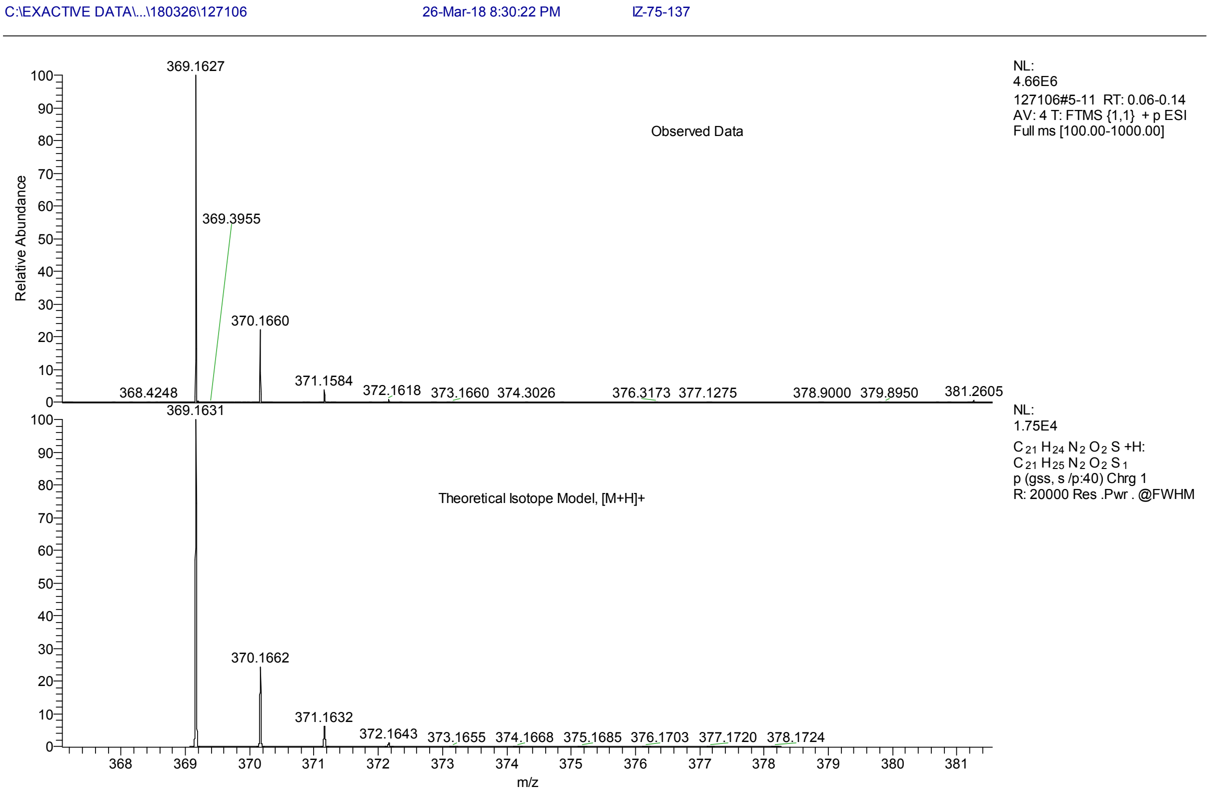

HR-MS of compound 3.1

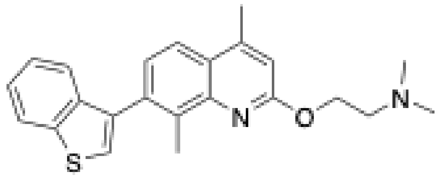

3.2

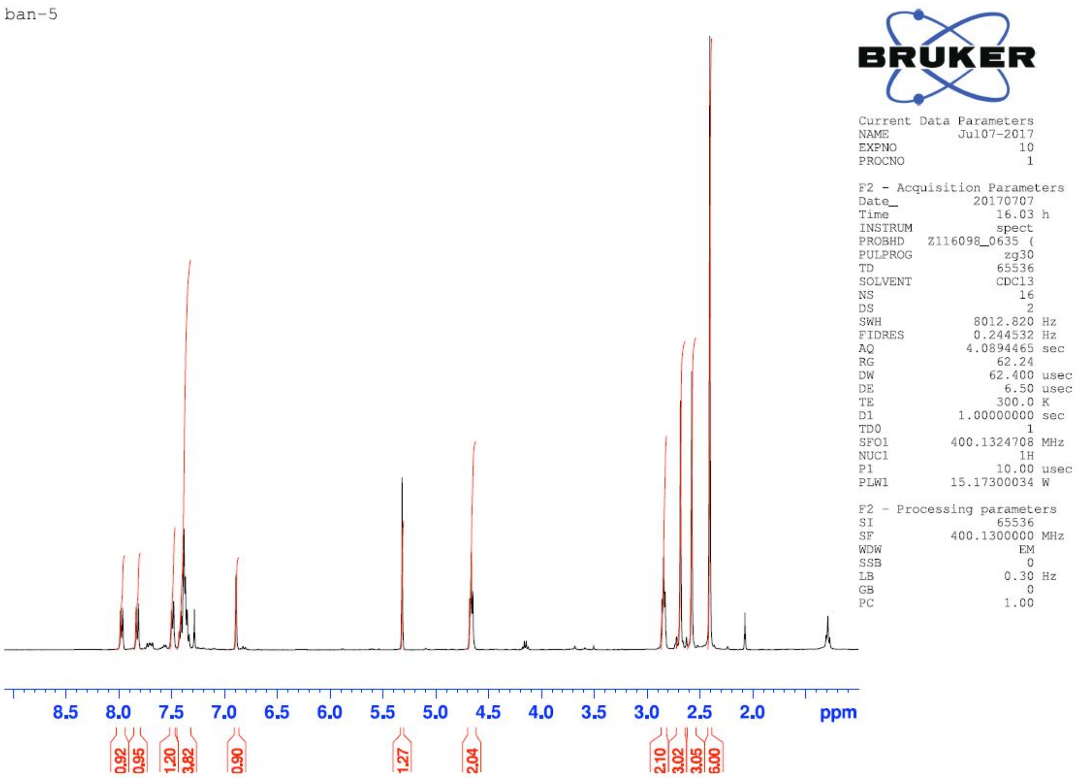

^{1}H-NMR of compound 3.2

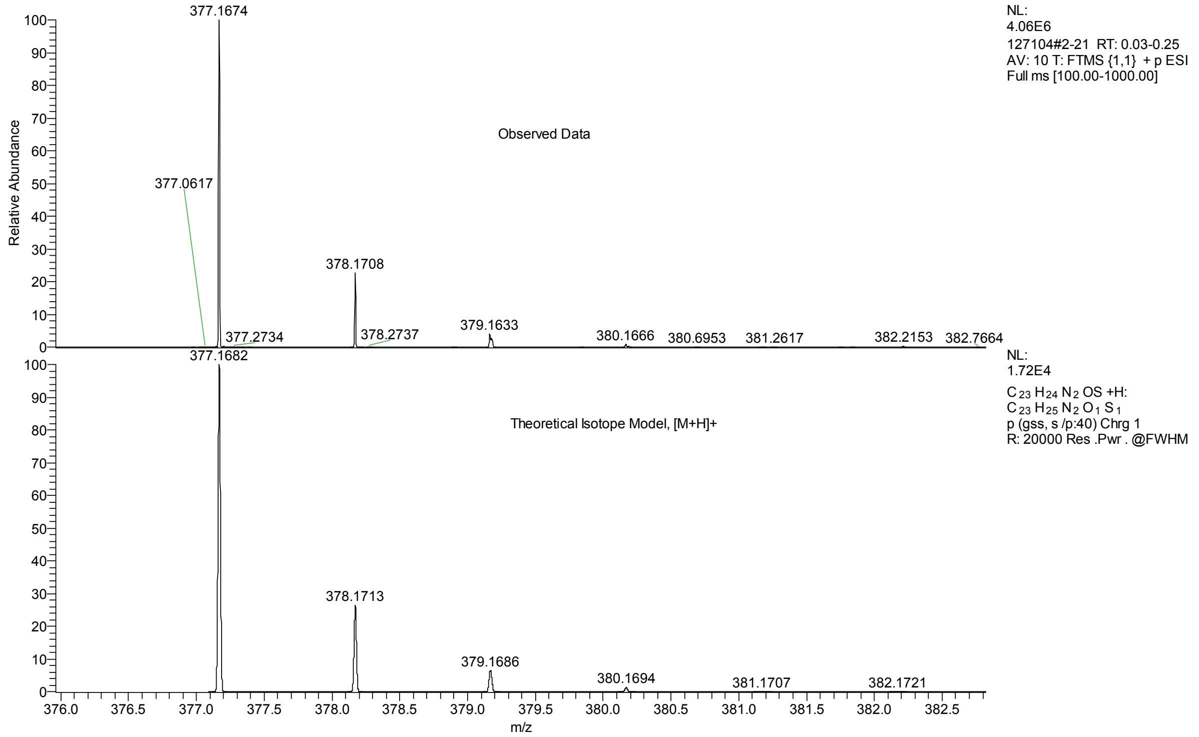

HR-MS of compound 3.1

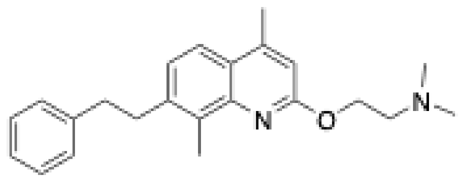

3.3

3.4

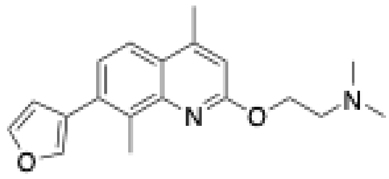

3.4

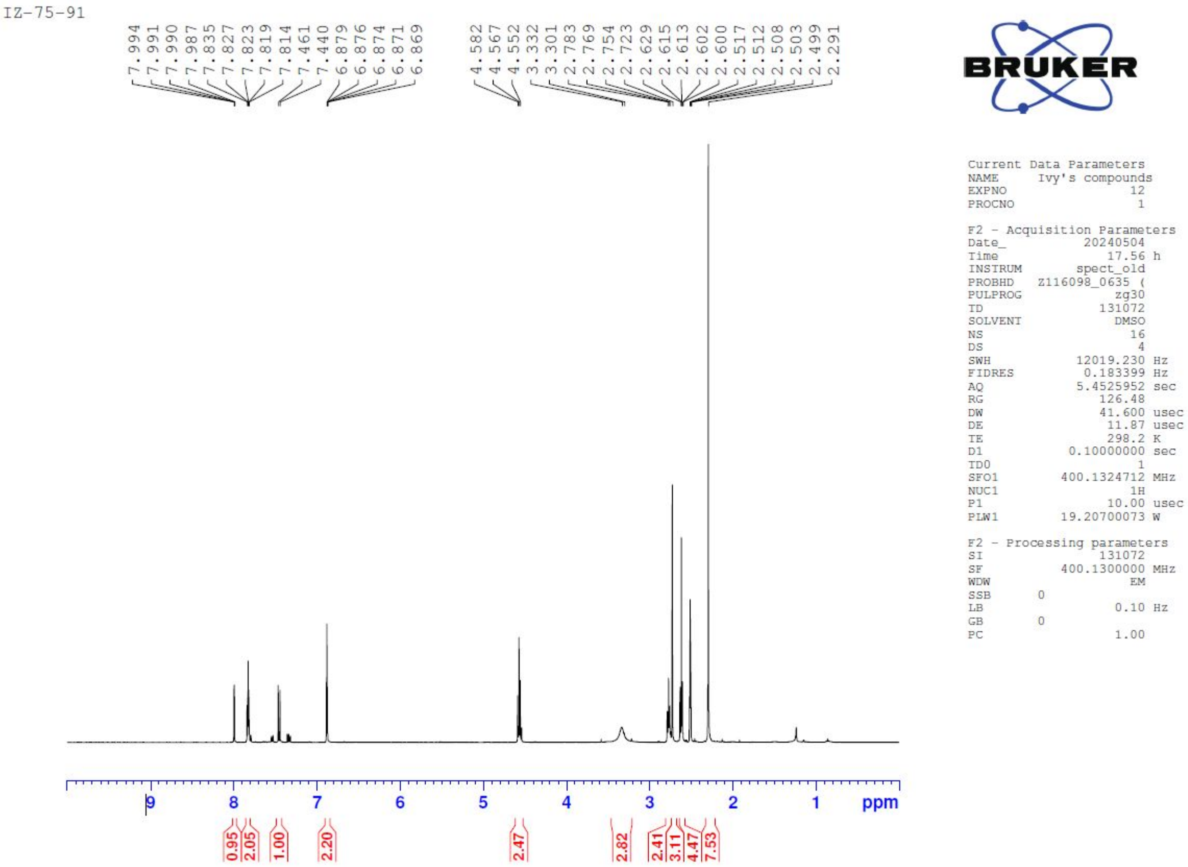

^{1}H-NMR of compound 3.4

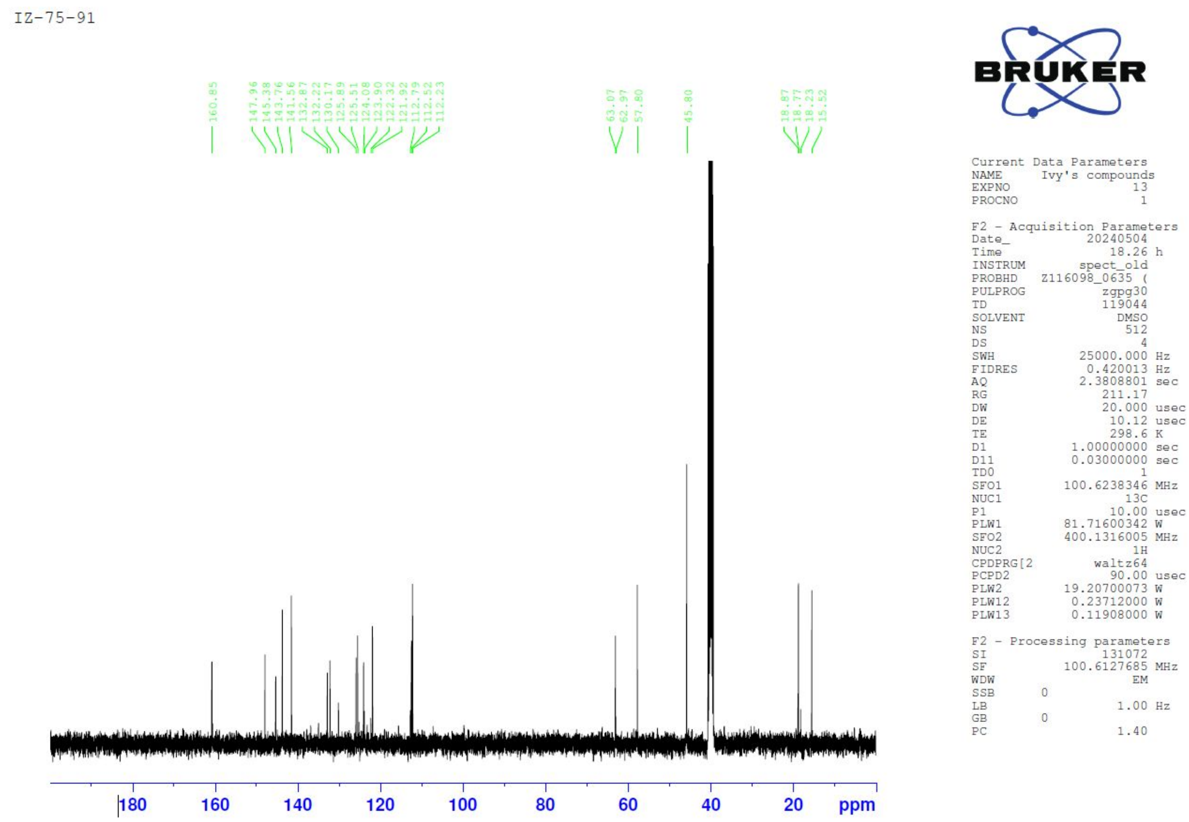

^{13}C-NMR of compound 3.4

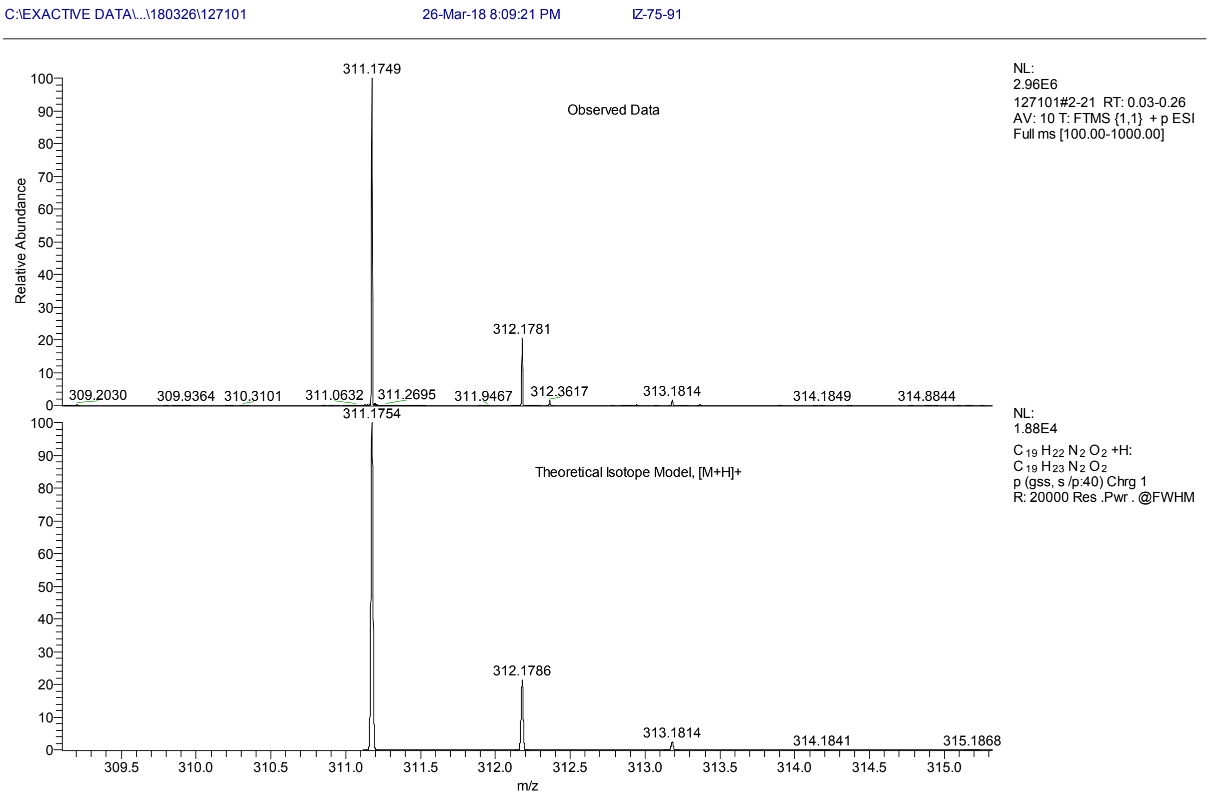

HR-MS of compound 3.4

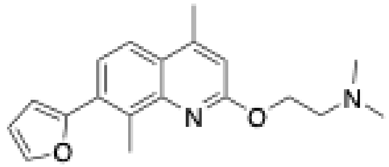

3.5

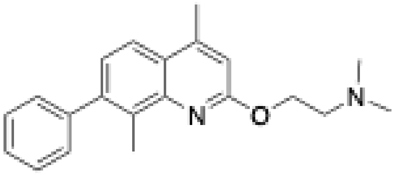

3.6

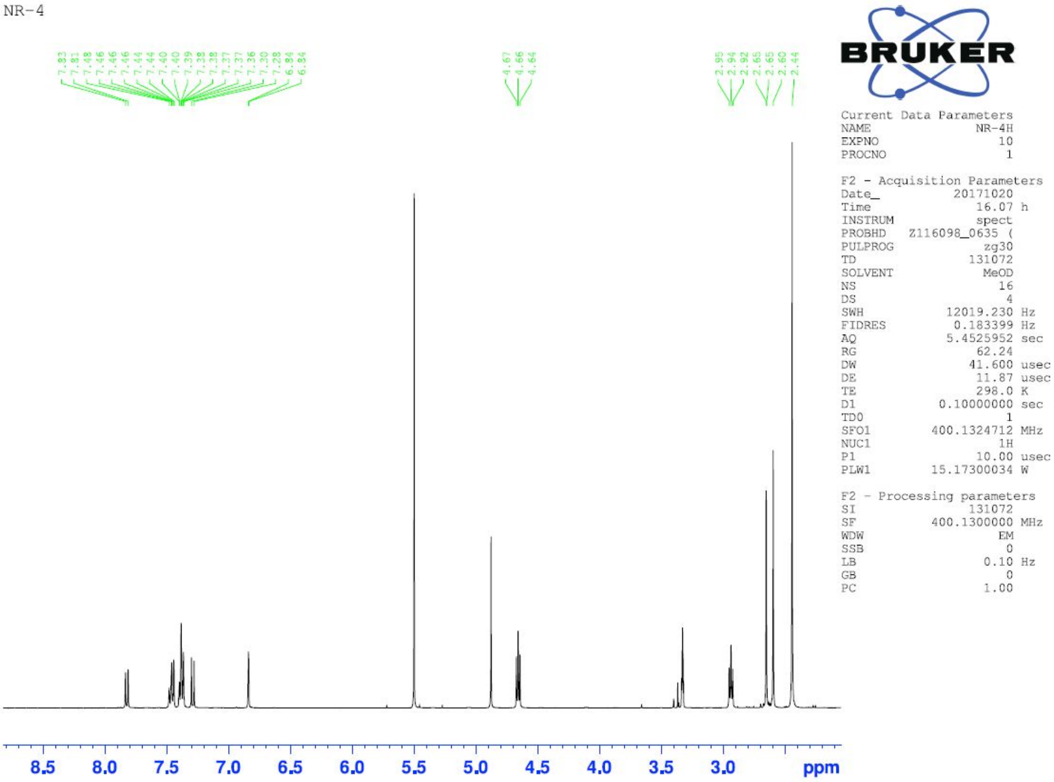

^{1}H-NMR of compound 3.6

3.7

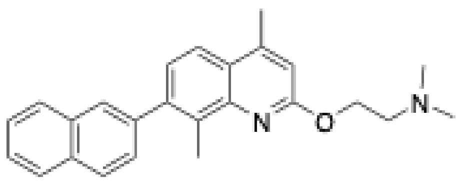

3.7

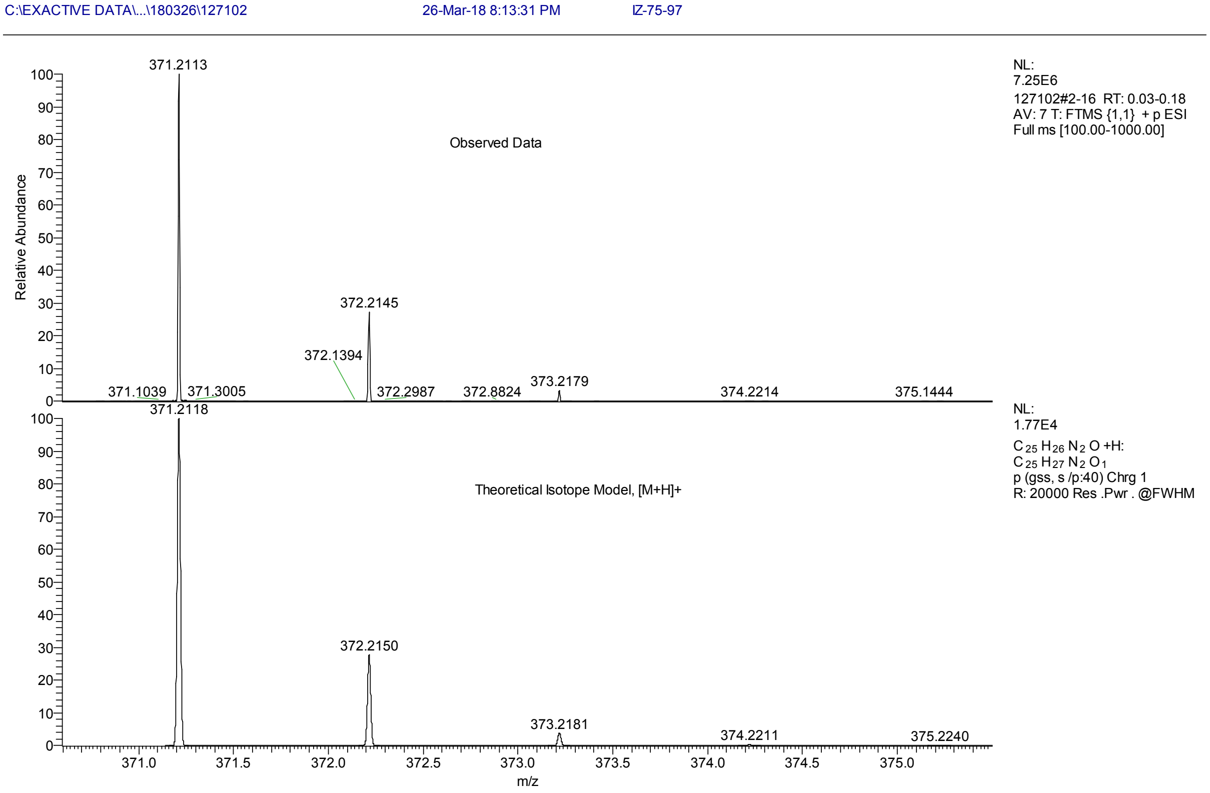

HR-MS of compound 3.4

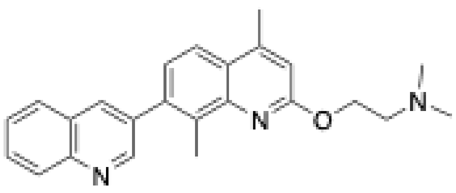

3.8

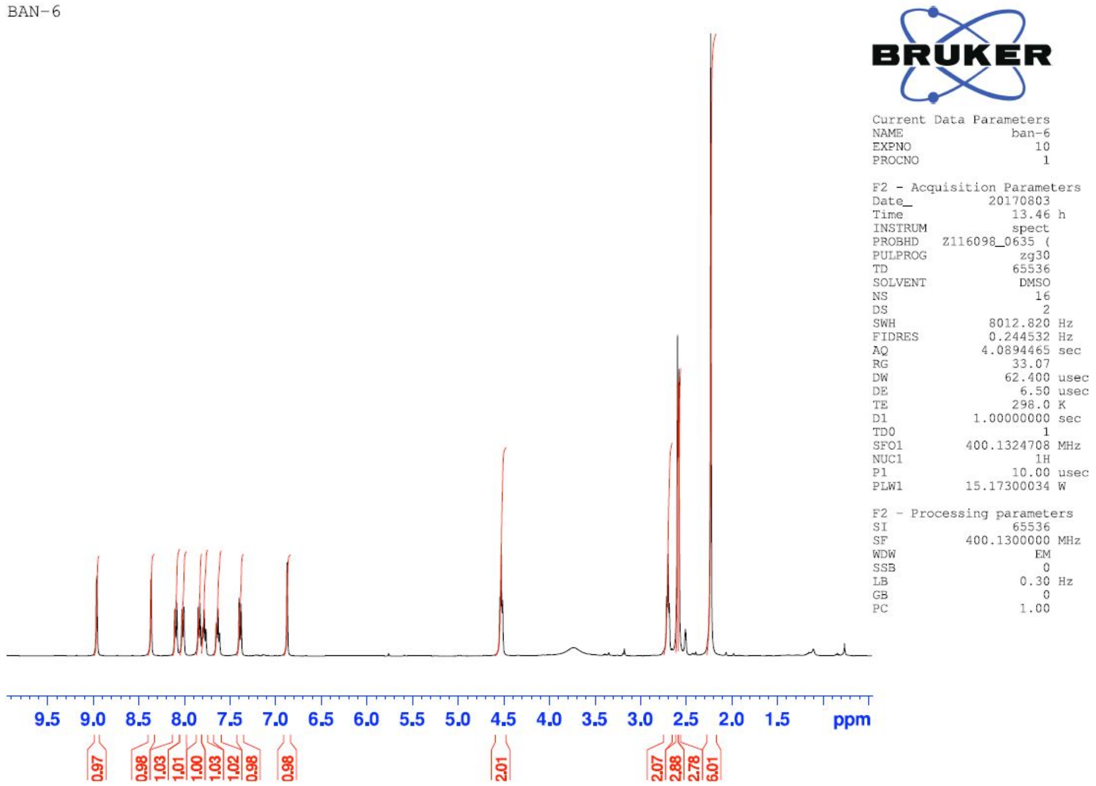

^{1}H-NMR of compound 3.8

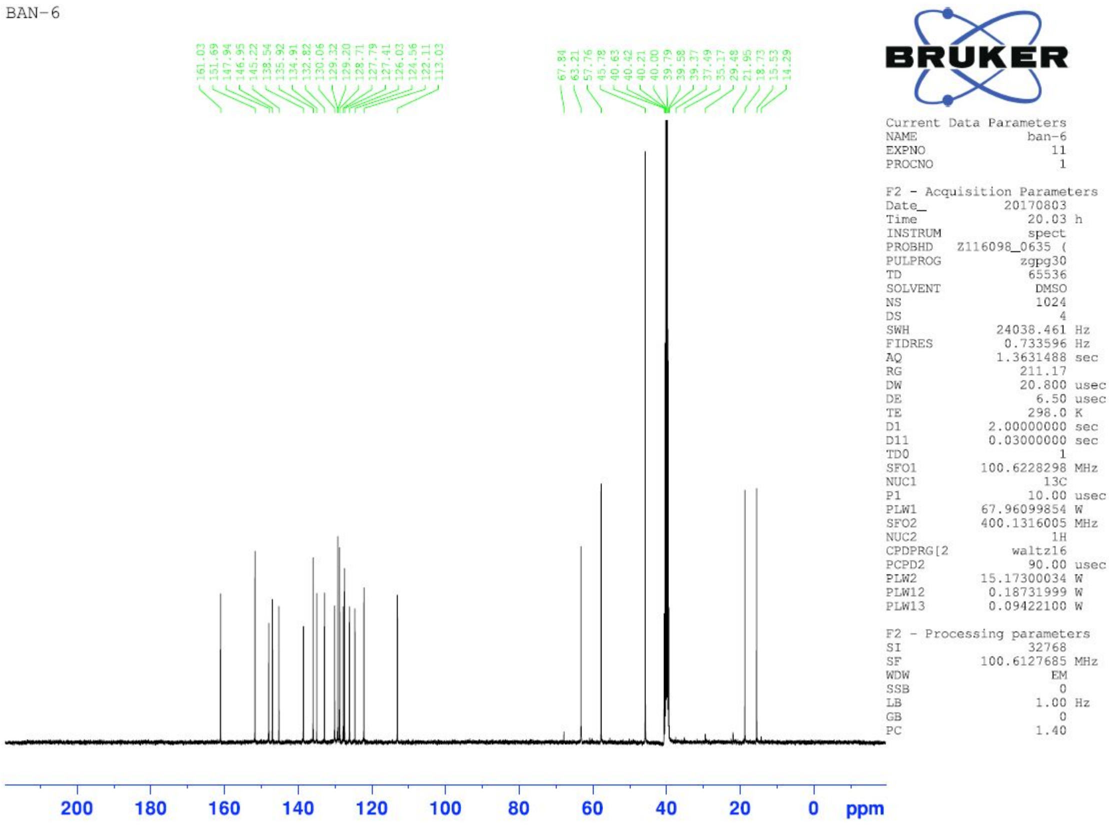

^{13}C-NMR of compound 3.8

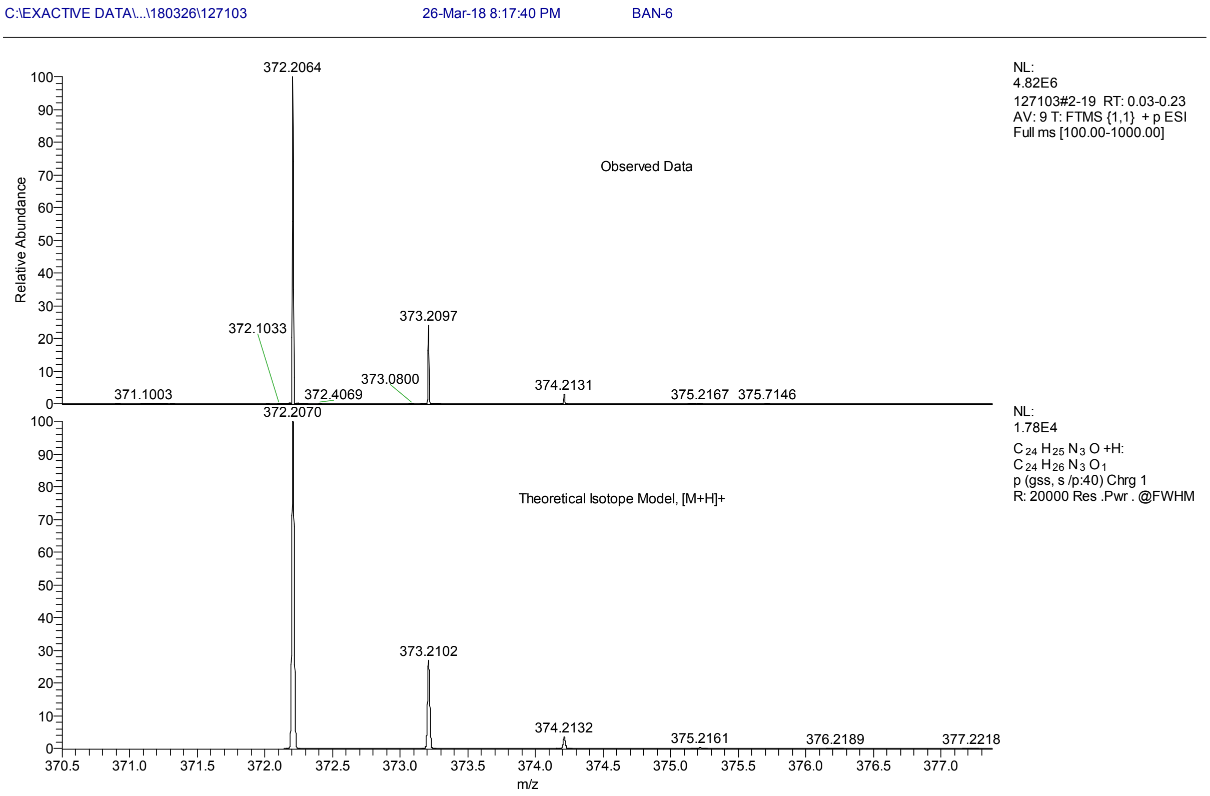

HR-MS of compound 3.8

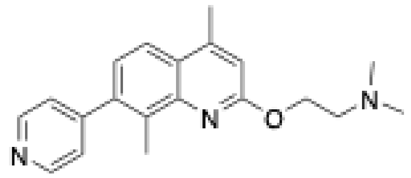

3.9

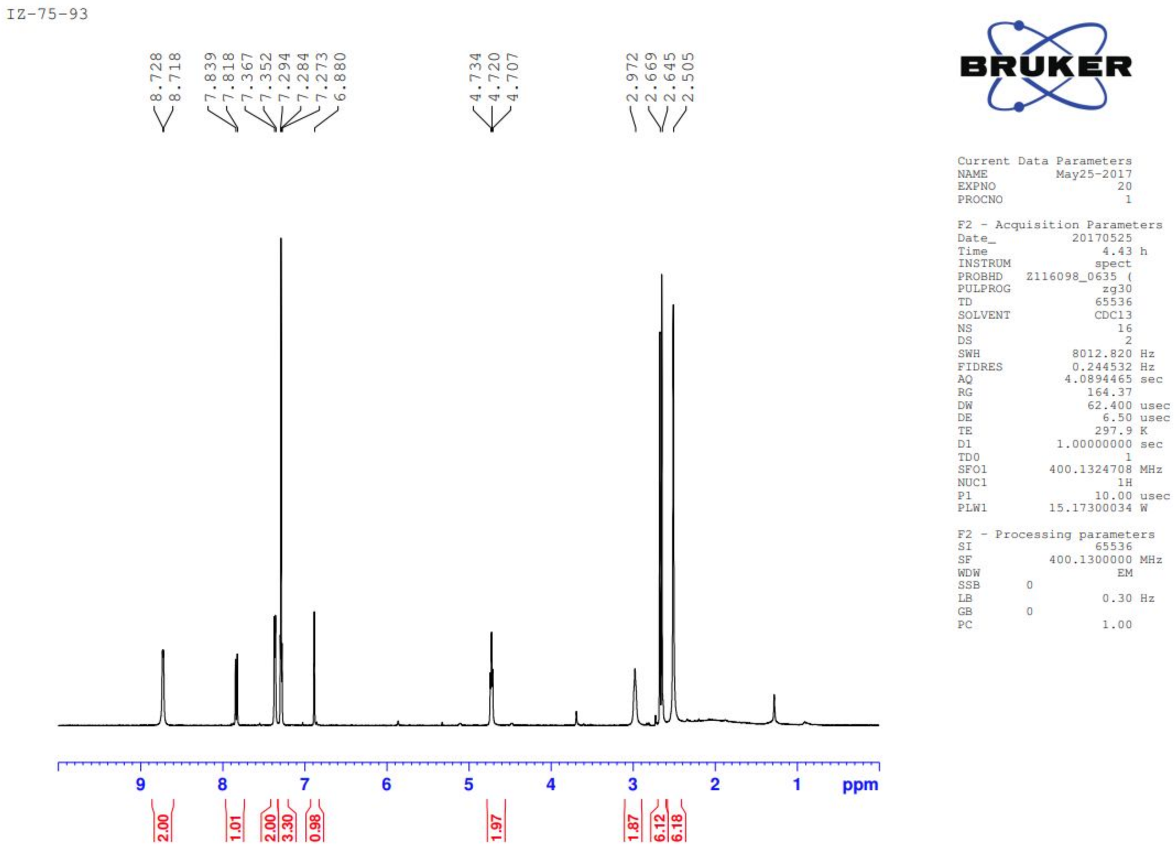

^{1}H-NMR of compound 3.9

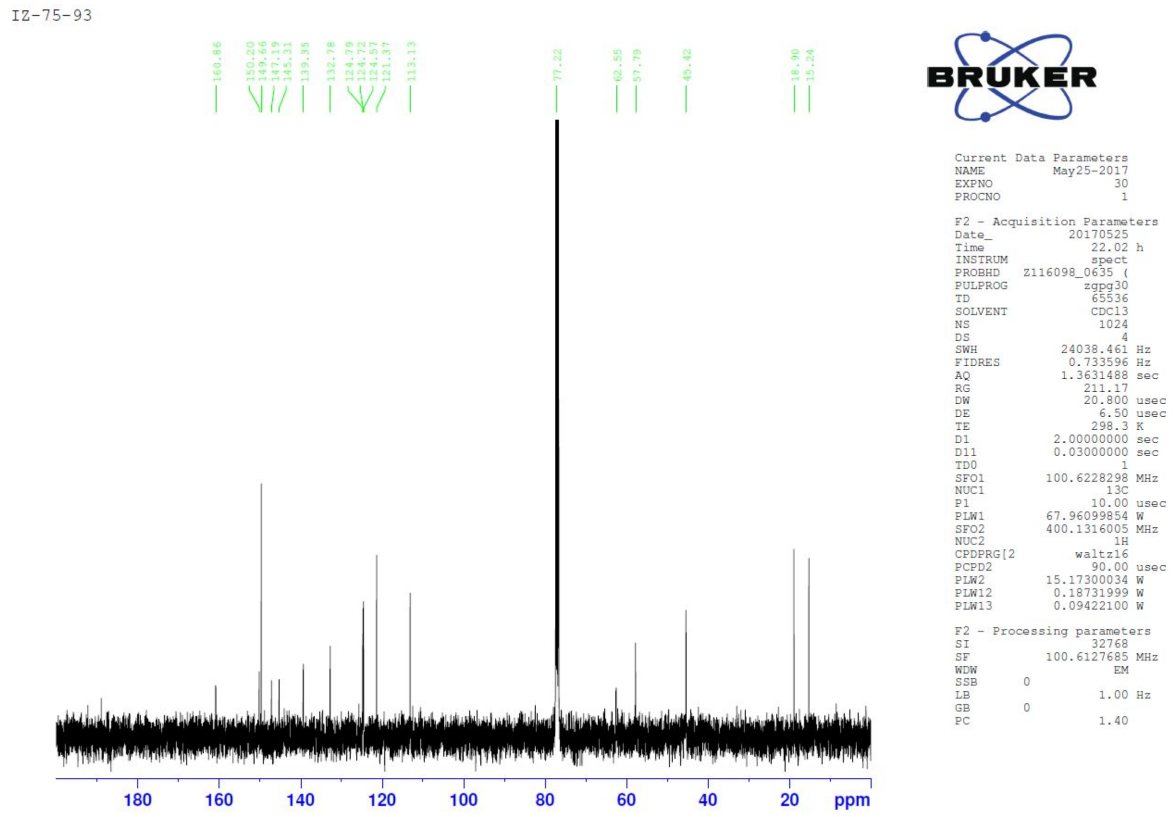

^{13}C-NMR of compound 3.9

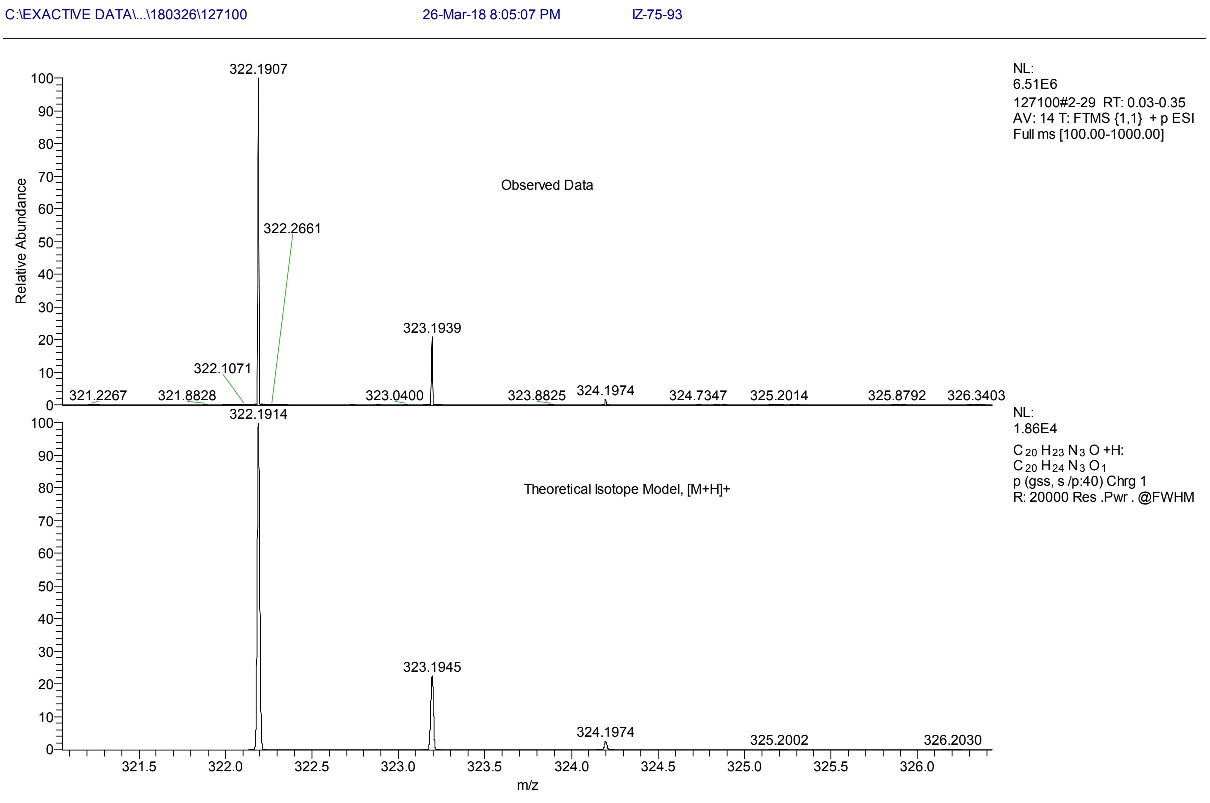

HR-MS of compound 3.9

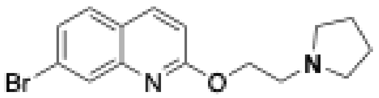

1.10

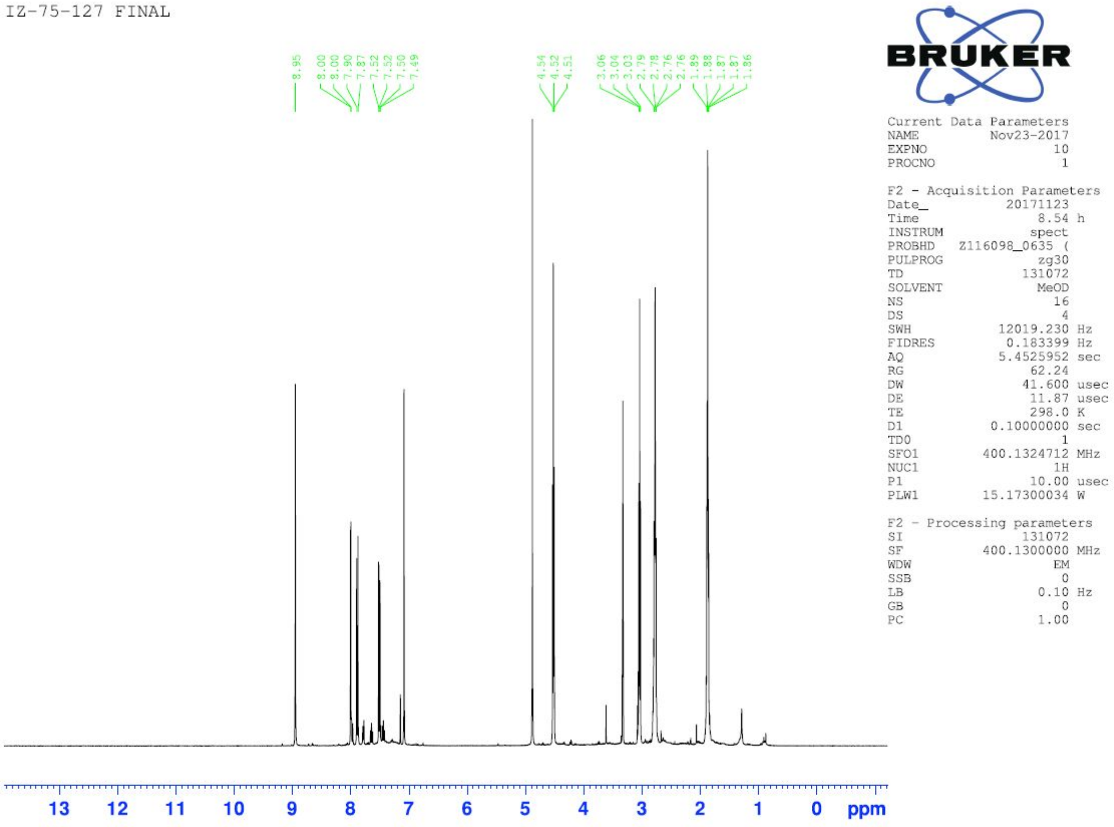

^{1}H-NMR of compound 1.10

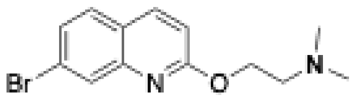

3.10

IZ-75-129 12'-24'

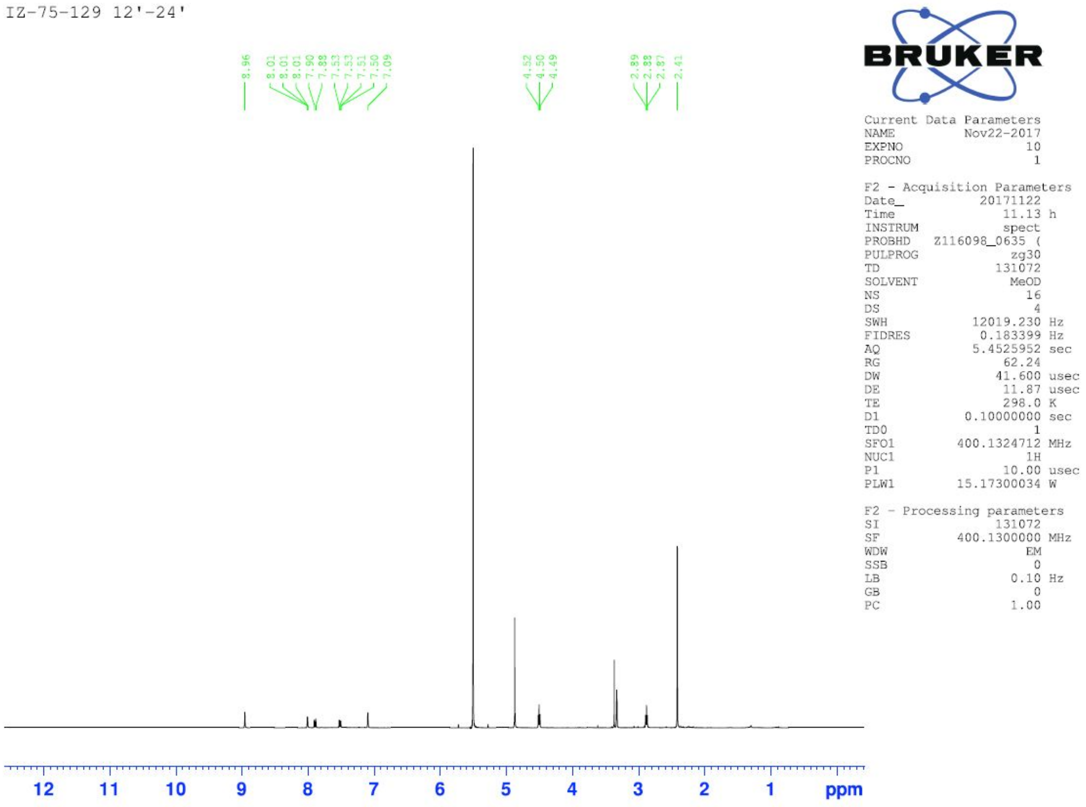

^{1}H-NMR of compound 3.10

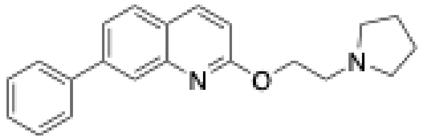

1.11

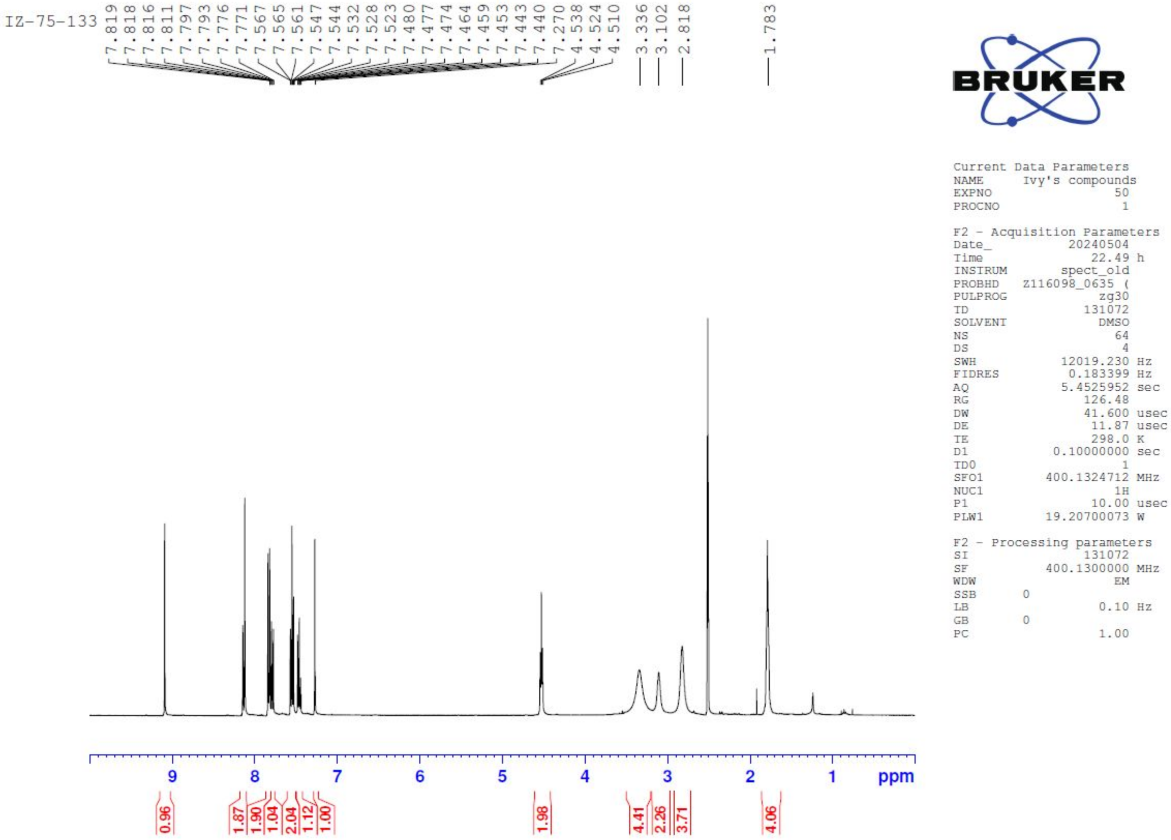

^{1}H-NMR of compound 1.11

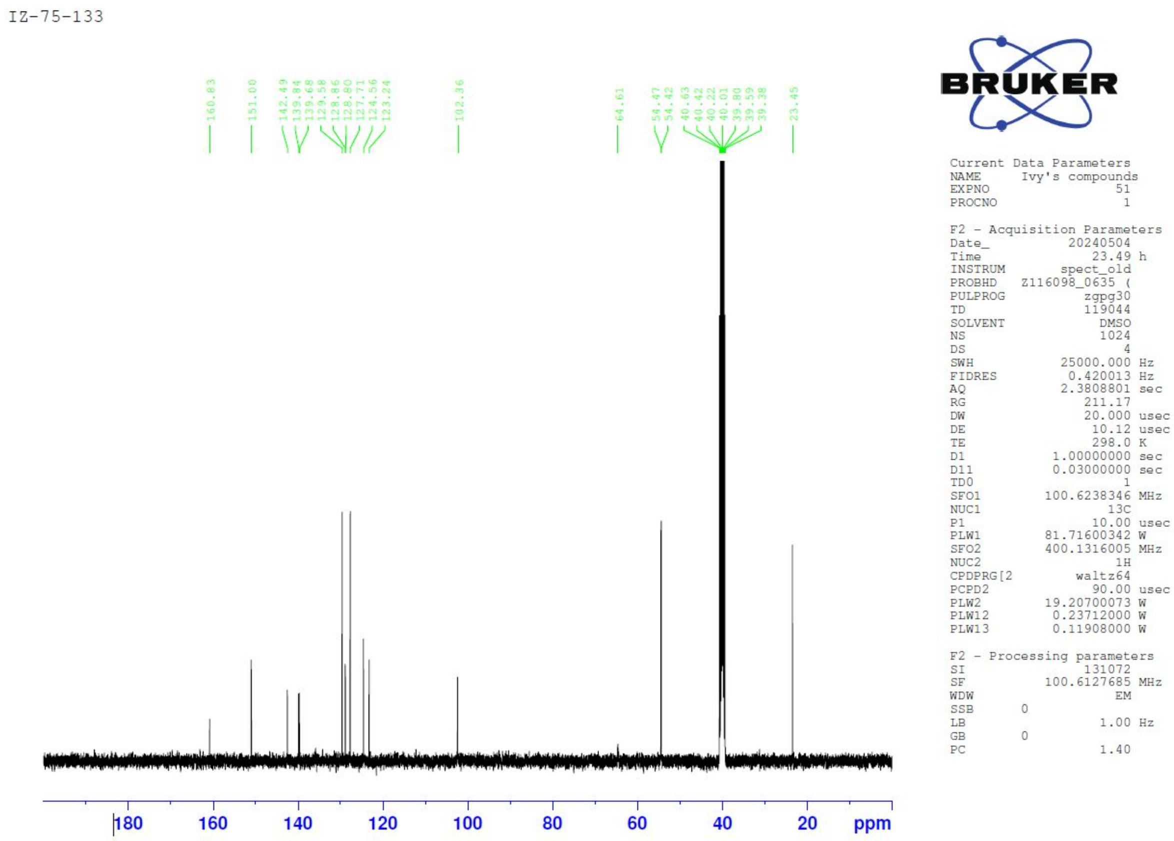

^{13}C-NMR of compound 1.11

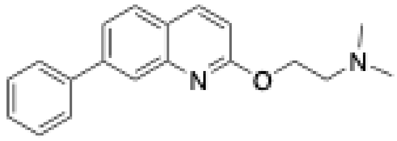

3.11

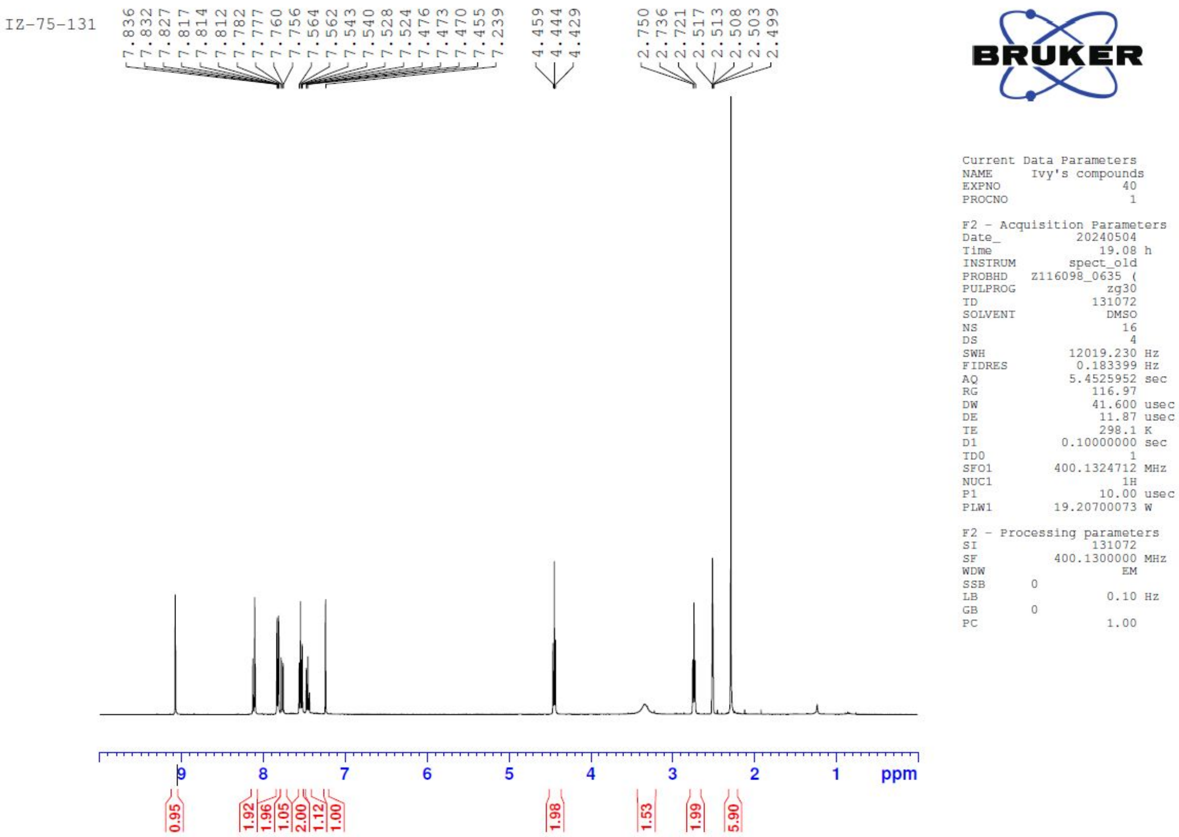

^{1}H-NMR of compound 3.11

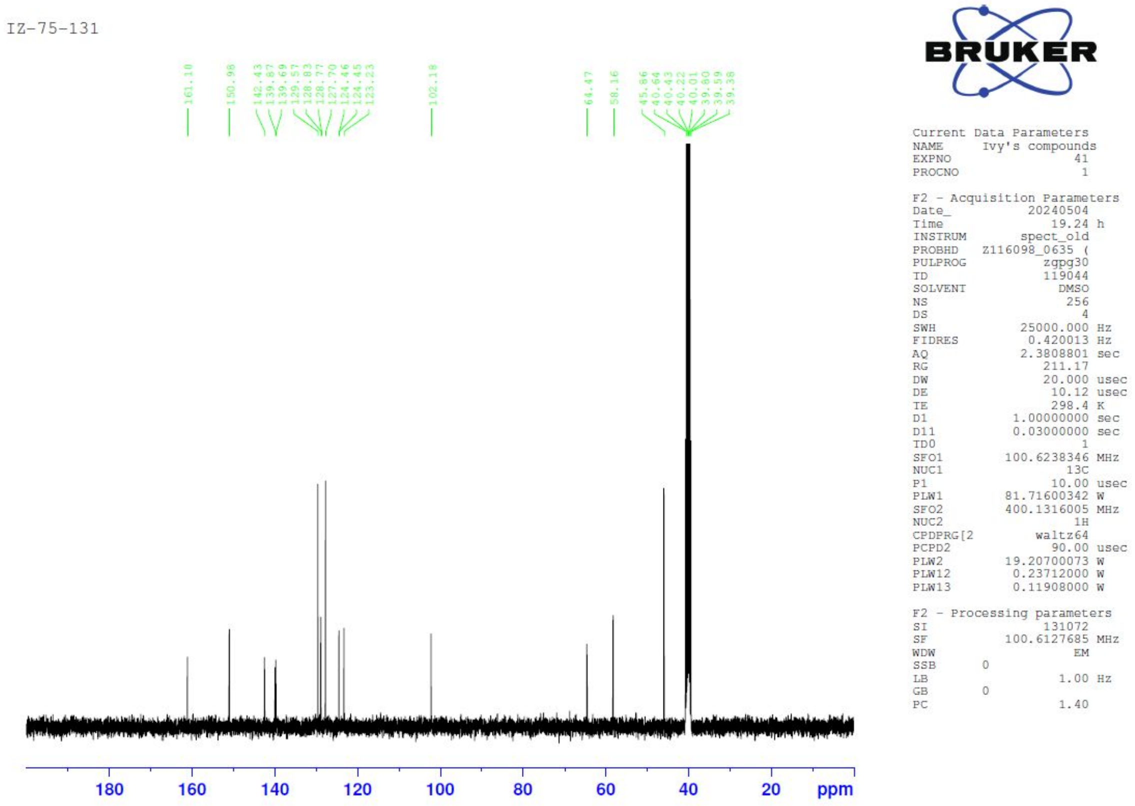

^{13}C-NMR of compound 3.11

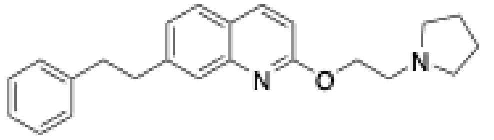

1.12

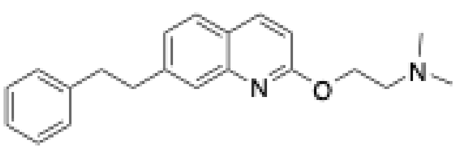

3.12

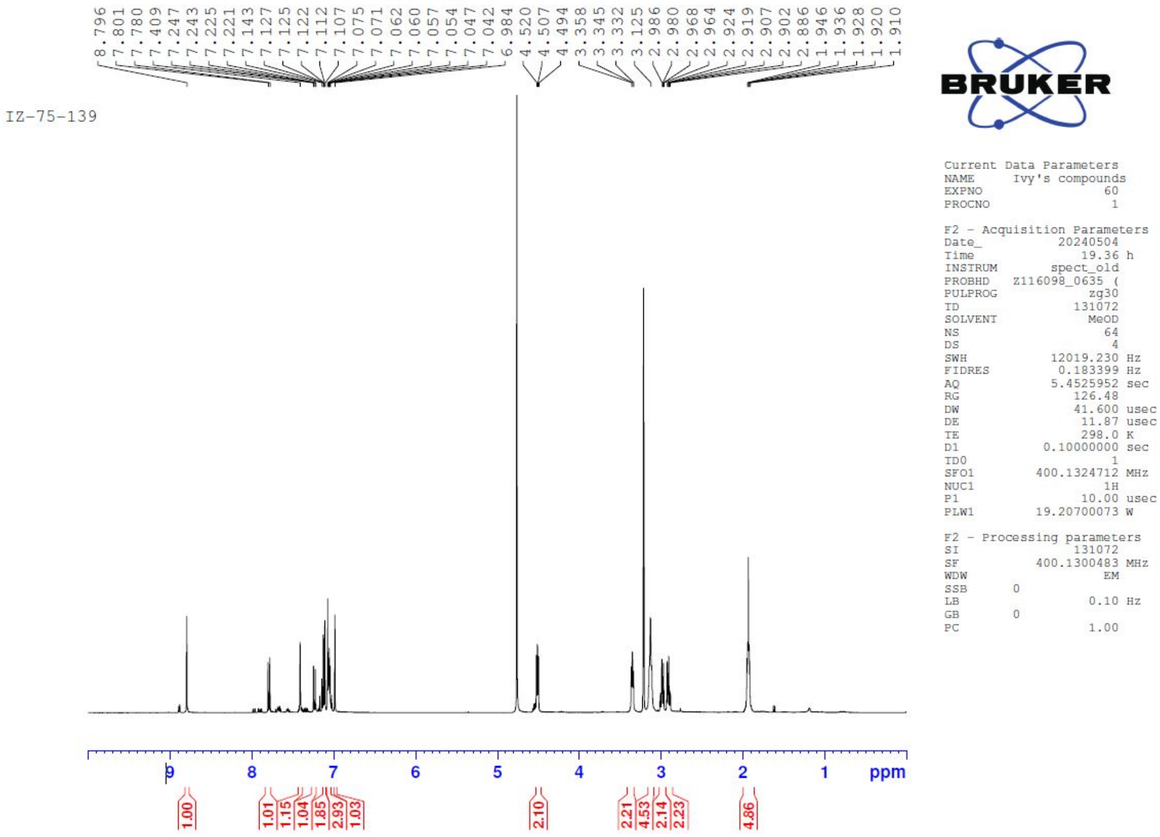

^{1}H-NMR of compound 3.12

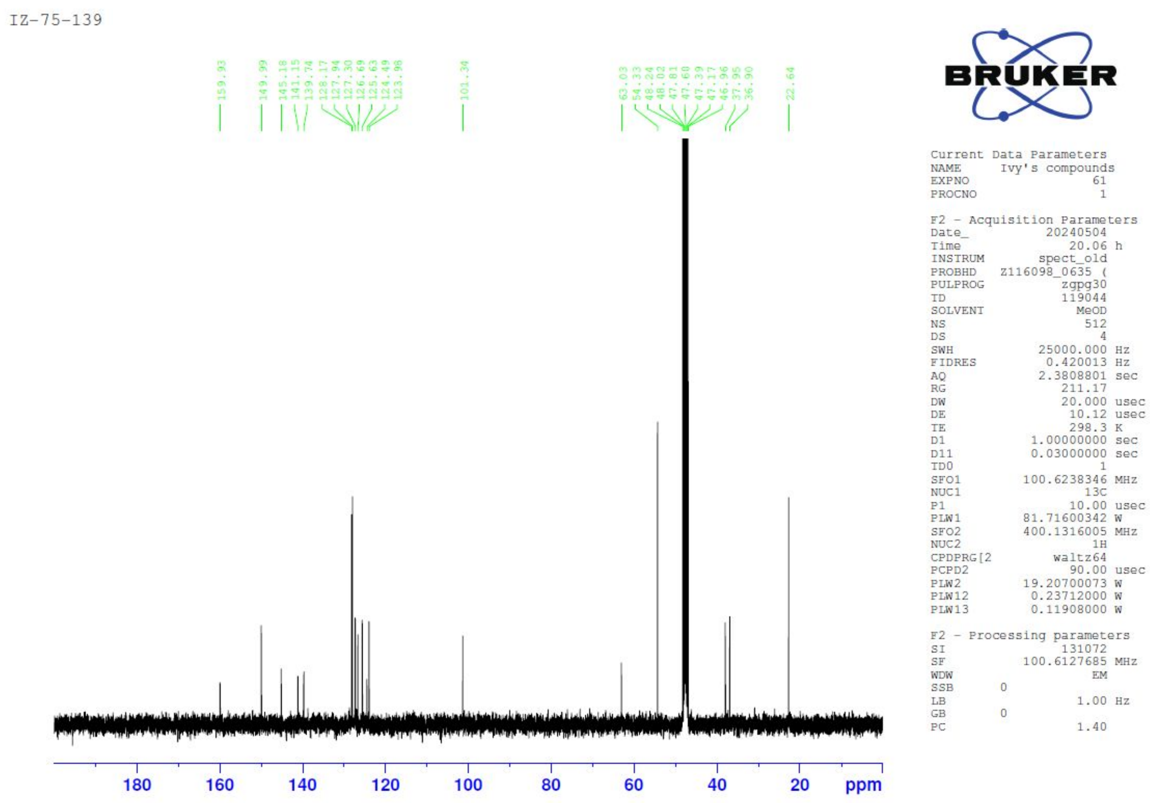

^{13}C-NMR of compound 3.12
